# The V223I substitution in hemagglutinin reduces the binding affinity to human-type receptors while enhancing the thermal stability of the H3N2 canine influenza virus

**DOI:** 10.3389/fmicb.2024.1442163

**Published:** 2024-07-22

**Authors:** Liling Liu, Fujun Wang, Ying Wu, Weiyong Mi, Yaping Zhang, Lei Chen, Dongxue Wang, Guohua Deng, Jianzhong Shi, Hualan Chen, Huihui Kong

**Affiliations:** ^1^State Key Laboratory for Animal Disease Control and Prevention, Harbin Veterinary Research Institute, CAAS, Harbin, China; ^2^Department of Biotechnology, Heilongjiang Vocational College for Nationalities, Harbin, China; ^3^Harbin Fuai Pet Hospital, Harbin, China

**Keywords:** canine influenza virus, H3N2, hemagglutinin, evolution, human-type receptor, thermal stability

## Abstract

Given the intimate relationship between humans and dogs, the H3N2 canine influenza viruses (CIVs) pose a threat to public health. In our study, we isolated four H3N2 CIVs from 3,758 dog nasal swabs in China between 2018 and 2020, followed by genetic and biological analysis. Phylogenetic analysis revealed 15 genotypes among all available H3N2 CIVs, with genotype 15 prevailing among dogs since around 2017, indicating the establishment of a stable virus lineage in dogs. Molecular characterization identified many mammalian adaptive substitutions, including HA-G146S, HA-N188D, PB2-I292T, PB2-G590S, PB2-S714I, PB1-D154G, and NP-R293K, present across the four isolates. Notably, analysis of HA sequences uncovered a newly emerged adaptive mutation, HA-V223I, which is predominantly found in human and swine H3N2 viruses, suggesting its role in mammalian adaptation. Receptor-binding analysis revealed that the four H3N2 viruses bind both avian and human-type receptors. However, HA-V223I decreases the H3N2 virus’s affinity for human-type receptors but enhances its thermal stability. Furthermore, attachment analysis confirmed the H3N2 virus binding to human tracheal tissues, albeit with reduced affinity when the virus carries HA-V223I. Antigenic analysis indicated that the current human H3N2 vaccines do not confer protection against H3N2 CIVs. Collectively, these findings underscore that the potential threat posed by H3N2 CIVs to human health still exists, emphasizing the necessity of close surveillance and monitoring of H3N2 CIVs in dogs.

## 1 Introduction

Influenza A viruses pose a significant threat to both avian species and certain mammals, including humans. These viruses are subtyped according to the genetic and antigenic characteristics of two surface glycoproteins, hemagglutinin (HA) and neuraminidase (NA). Wild birds, particularly waterfowl, serve as the primary natural reservoirs for influenza A viruses, harboring 16 HA and 9 NA subtypes (Dowdle et al., 1975; Wille and Holmes, 2020). Recent discoveries have expanded our understanding with the detection of novel subtypes, such as H17N10 and H18N11, in bats (Tong et al., 2012, 2013). In history, four pandemics have been caused by influenza A viruses, resulting in millions of human deaths, including the 1918 Pandemic (H1N1 virus), the 1957–1958 Pandemic (H2N2 virus), the 1968 Pandemic (H3N2 virus), and the 2009 H1N1 Pandemic (H1N1 pdm09 virus) (Subbarao and Katz, 2000). Most recently, the emergence of H7N9 viruses led to an endemic in 2013, with over 1,500 human infections and 600 fatalities (Gao et al., 2013; Zhang et al., 2013b; Shi et al., 2023). Thus, the ongoing risk of cross-species transmission of influenza A viruses from animal reservoirs to humans remains a significant public health concern.

Among influenza A virus subtypes, H3N2 variants have been documented to infect various mammalian species alongside waterfowl. In the last century, a recombinant form of H3N2 viruses containing two genes from a duck virus (H3 and PB1) in a background of the human H2N2 strain was found to infect humans in 1968, and a recombinant form of H3N2 strains from avian and humans sources was first found in in the United States swine population in 1998 (Zhou et al., 1999; Jester et al., 2020). However, at the beginning of 21st century, avian-origin H3N2 viruses were reported to infect canines, mink, and feline, with a stable lineage established only in canines (Gagnon et al., 2009; Jeoung et al., 2013; Wasik et al., 2021). The first H3N2 canine influenza virus (CIV), genetically closest to the H3N2 avian influenza virus (AIV) circulating in aquatic ducks in South Korea, was isolated in 2006 in Guangdong, China (Li et al., 2010). Since 2008, H3N2 CIVs have been frequently isolated in South Korea, Thailand, and China (Song et al., 2008; Li et al., 2010; Bunpapong et al., 2014). In 2015, a golden retriever in Chicago was infected with an H3N2 CIV genetically similar to those circulating in Asia (Voorhees et al., 2017), leading to the virus spreading to other states across the United States, causing respiratory disease in thousands of dogs. According to released sequence data, H3N2 CIVs are now predominantly circulating among dogs in China and the United States (Oduoye et al., 2023; Chen et al., 2024), where they are considered endemic.

Dogs, as one of the closest companion animals to humans, may easily transmit CIVs to humans through direct contact or aerosol transmission. Generally, CIVs are considered to pose a low threat to human beings. Notably, Chen et al. demonstrated that recent H3N2 CIVs have become capable of recognizing α2,6-linked sialic acids (human-type receptors) and transmitting efficiently via respiratory droplets in the ferret model (Chen et al., 2023), highlighting their potential cross-species transmission threat. Additionally, dogs may serve as mixing vessels for the adaptation of avian influenza viruses to humans. Reassortment ranks among the top factors in generating a virus with pandemic potential, as evidenced by four historical human pandemics and the emergence of H7N9 viruses in 2013. Serological data have revealed that canines can be infected with human influenza viruses, such as the human H1N1pdm09 virus and seasonal H3N3 virus (Sun et al., 2014), which may facilitate reassortment between H3N2 CIVs and human viruses. Moreover, evidence has shown that dogs are also susceptible to avian H9N2 viruses (Zhang K. et al., 2013), which provide internal gene cassettes as donors for human-infecting avian influenza viruses, such as H7N9 (Zhang Q. et al., 2013), H10N8 (Deng et al., 2015), and H3N8 viruses (Cui et al., 2023). Notably, an H3N1 CIV emerged via reassortment between a human H1N1 and a canine H3N2 virus in 2012 (Song et al., 2012), and an H3N2 CIV acquired the polymerase acidic (PA) gene from an H9N2 virus in 2015 (Lee et al., 2016). Thus, controlling H3N2 CIVs in dogs is critical to prevent the emergence of CIVs with human-infecting potential.

The increasing isolation rate of H3N2 CIVs in recent years highlights the persistent threat of cross-species transmission (Martinez-Sobrido et al., 2020; Li et al., 2021; Ou et al., 2022; Chen et al., 2023, 2024). To deepen our understanding of the potential risks associated with H3N2 CIVs, we isolated four strains from 3,758 canine nasal swabs collected between 2018 and 2020 in multiple provinces of China. Subsequently, we conducted comprehensive investigations into the genetic characteristics, receptor-binding properties, and antigenicity of these H3N2 CIV strains. Our findings provide crucial insights for assessing the zoonotic transmission potential of H3N2 CIVs.

## 2 Materials and methods

### 2.1 Virus isolation

Between 2018 and 2020, a total of 3,758 nasal swabs were collected from dogs in pet shelters and hospitals across five provinces and one autonomous region in China, including Heilongjiang, Jiangsu, Zhejiang, Hebei, Yunnan Province, and Ningxia Hui Autonomous Region. The nasal swabs were preserved in phosphate-buffered saline (PBS) supplemented with penicillin (2,000 U/mL) and streptomycin (2,000 μg/mL). Virus isolation was conducted by centrifuging the samples at 6,000 r/min for 3 min to remove impurities, followed by inoculation of the supernatant into 9- to 11-day-old embryonated chicken eggs. After incubation at 37°C for 48 h, allantoic fluid from positive samples underwent hemagglutinin inhibition (HI) assay using homemade H3 subtype-specific antisera to confirm the H3 subtype, while the NA subtype was determined through Sanger sequencing and analyzed using the Genetic Analyzer (Applied Biosystems™, United States). All isolated viruses underwent biological cloning three times via limiting dilution in embryonated specific-pathogen-free (SPF) eggs before being collected and stored at −80°C for further analysis.

### 2.2 Phylogenetic analysis

The genomes of four CIVs, which were deposited in the GISAID database,^1^ were sequenced using Sanger sequencing. To sequence the gene segments, viral RNA was extracted using the TIANamp Virus RNA Kit (TIANGEN, Beijing, China), and was reverse transcribed with a mixture of universal 12 bp primers (AGCRAAAGCAGG). Subsequently, PCR amplification was performed with indicated primers. The raw data generated by the genetic analyzer were assembled using the DNASTAR package (DNAstar v7.0). The primer sequences used are available upon request. To construct the phylogenetic tree of each gene segment, sequences were downloaded from databases such as GenBank^2^ or GISAID,^3^ aligned using the MAFFT algorithm, and cleaned with the trimAI module in the PhyloSuite (PhyloSuite v1.2.3) software package. The phylogenetic trees of the genes were analyzed using the Maximum Likelihood (ML) method with a bootstrap value of 20,000. A ≥ 98% cutoff was applied to categorize the gene segments into different groups. The tree was edited and presented using FigTree (FigTree v1.4.4).

### 2.3 Receptor-binding preference analysis

The receptor-binding properties of H3N2 CIVs were assessed using a solid-phase binding assay with two synthesized glycopolymers: α2,3-sialylglycopolymer (Neu5Aca2–3Galb1-4GlcNAcb1-pAP-alpha-polyglutamic acid (α-PGA)) and α2,6-sialylglycopolymer (Neu5Aca2–6Galb1-4GlcNAcb1-pAP-alpha-polyglutamic acid (α-PGA)), which mimic avian- and human-type receptors, respectively (Neumann et al., 2014). To purify the virus, the allantoic fluid was centrifuged at 28,000 rpm/min for 2 h on a 30% sucrose layer. For the analysis, purified viruses with a series of twofold dilutions were incubated on a plate coated with the indicated glycopolymers at 4°C overnight. Subsequently, the washed plate was fixed with 4% formalin and washed again five times with PBST (PBS containing 0.1% Tween 20). After incubation with the primary antibody (lab-made chicken antiserum) and matched secondary antibody (Sigma–Aldrich, St. Louis, MO, United States) alternately, the plate was subjected to color development with O-phenylenediamine (Sigma–Aldrich, St. Louis, MO, United States). Absorbance was measured at 490 nm.

### 2.4 Reverse genetics

To rescue a virus, we used a method previously developed in our laboratory (Kong et al., 2019). First, 293 T cells were seeded in 6-well plates 1 day prior to transfection. Following the manufacturer’s protocol, these cells were transfected with 4 μg of a mixture comprising the surface genes of the H3N2 virus and the backbone genes of A/Puerto Rico/8/1934 (H1N1, PR8) using the transfection agent Lipofectamine® 2000 (Invitrogen, Waltham, MA, United States). Six hours post-transfection, the culture medium was replaced with Opti-MEM supplemented with 0.125 μg/mL of TPCK-trypsin (Sigma, St. Louis, MO, United States). The cells were then incubated at 37°C with 5% CO_2_ for 48 h. After this incubation period, the supernatant from the transfected cells was collected and used to inoculate ten-day-old embryonated chicken eggs.

### 2.5 Biolayer interferometry

The receptor-binding specificity of influenza A virus was evaluated by using the Octet Red 96 system (FortéBio, United States). Two types of biotinylated glycans, Neu5Ac(α2-3)Gal(β1-4)GlcNAc (3SLN, avian-type receptor) and Neu5Ac(α2-6)Gal(β1-4)GlcNAc (6SLN, human-type receptor), were used in this study as previously described (Kong et al., 2019). Briefly, the glycans were immobilized on the streptavidin (SA) biosensors and then incubated with 128 HA units of the indicated viruses in HBS-EP buffer (150 mM NaCl, 10 mM HEPES (pH 7.4), 3 mM EDTA, and 0.005% surfactant P20) containing 10 mM oseltamivir. Binding was performed at 30°C for 1, 500 s.

### 2.6 Viral affinity to tissues

The binding properties of viruses to normal human tracheal tissues, purchased from Zhongke Guanghua Intelligent Biotechnology Co., Ltd.,^4^ were assessed using an indirect immunofluorescence assay, following established protocols (Zhang et al., 2012; Czudai-Matwich et al.). Tissue sections were prepared in-house and subjected to deparaffinization, rehydration, and incubation with 5% bovine serum albumin (BSA) in PBS for 2 h at room temperature. After washing three times with PBST, the sections were exposed to viral suspensions (64 HA units in PBS) at 4°C overnight. Subsequently, the sections were treated with primary antibodies and FITC-labeled secondary antibodies alternately. Following staining with DAPI, the sections were examined under a microscope for analysis.

### 2.7 Hemagglutinin inhibition assay

To evaluate the antigenicity of H3N2 CIVs, we generated chicken antiserum and ferret antisera with the indicated H3N2 CIVs and human viruses. The protocols to generate antiserum were approved by the Committee on the Ethics of Animal Experiments of Harbin Veterinary Research Institute, with approval numbers 2020–01-01JiPi for th ferret experiment and 220,518-02-GR for the chicken experiment, respectively. For HI assay, a two-fold serial dilution of serum is prepared across the rows in a V-bottom-shaped 96-well microtiter plate. Subsequently, the sera were carefully combined with 25 μL of virus solution containing 4 HA Units, and the mixture was allowed to incubate for 30 min at ambient temperature. Following this incubation period, 50 μL of 0.5% chicken red blood cells (CRBC) suspension was added to the serum-virus mixtures, and the mixtures were further incubated for an additional 45 min at room temperature. The HI titer was determined as the highest serum dilution that effectively inhibits CRBC agglutination.

## 3 Results

### 3.1 Phylogenetic analysis

Between 2018 and 2020, a total of 3,758 nasal swabs from dogs were collected at pet shelters and hospitals across China, leading to the successful isolation of four H3N2 canine influenza virus (CIV) strains: A/canine/Heilongjiang/1/2018 (CN/HLJ/1/18), A/canine/Heilongjiang/1/2019 (CN/HLJ/1/19), A/canine/Heilongjiang/2/2019 (CN/HLJ/2/19), and A/canine/Heilongjiang/3/2019 (CN/HLJ/3/19). The eight gene segments of each isolated CIV were subjected to genetic analysis. Analysis of nucleotide sequence identities between each pair of H3N2 CIVs isolated in this study revealed remarkable similarity, with identities exceeding 99.0% across all eight genes: HA (99.0–100.0%), NA (99.6–100.0%), Polymerase Basic Protein 2 (PB2) (99.6–100.0%), Polymerase Basic Protein 1 (PB1) (99.5–100.0%), Polymerase Acidic Protein (PA) (99.3–100.0%), Nucleoprotein (NP) (99.5–100.0%), Matrix (M) (99.5–100.0%), and Non-structural Protein (NS) (99.5–100.0%). These findings indicate that the four CIVs share high genetic similarity and likely originated from the same ancestor.

**Figure 1:**
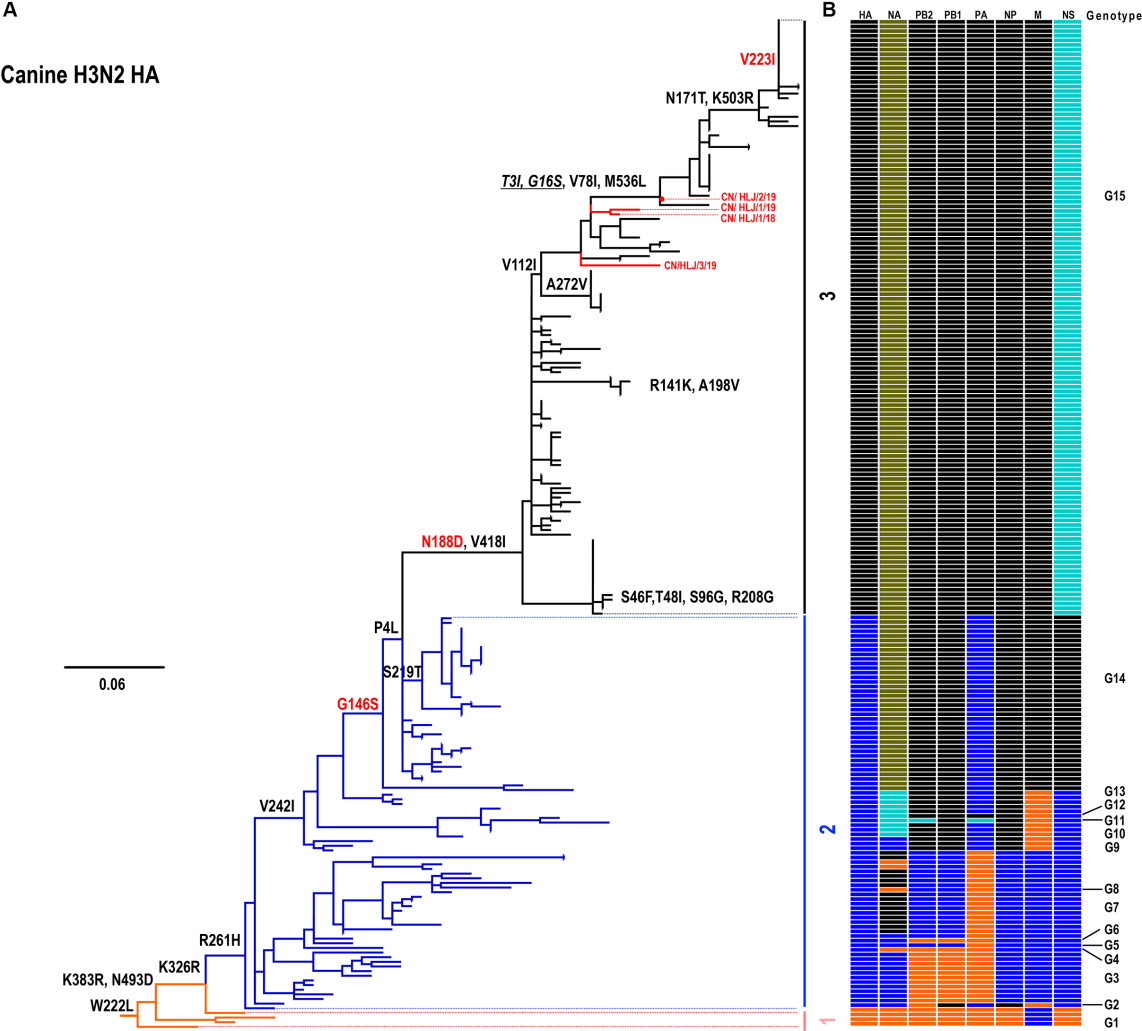
Phylogenetic analysis of H3N2 CIVs. **(A)** Phylogenetic tree of the HA gene. The tree was constructed using the Maximum Likelihood method and rooted to the A/canine/Guangdong/1/2006 (H3N2) virus. Sequences with a nucleotide identity greater than 98% were grouped together. **(B)** Genotype analysis of canine H3N2 viruses. The eight gene segments are indicated at the top of each bar. The genotype of all H3N2 viruses was determined based on the combination of different groups observed in the trees presented in Figure 1 and Supplementary Figure S1.

To understand the evolutionary patterns of H3N2 canine influenza viruses (CIVs), the genomes of four isolated H3N2 CIVs were subjected to phylogenetic analysis alongside all available H3N2 CIVs downloaded from databases. The evolutionary relationships of the H3N2 CIVs were presented in a tree constructed using Maximum Likelihood methods (Figure 1A). Based on nucleotide similarities among genes, distinct groupings were observed: HA (96.1–100%), NA (94.6–100%), PB2 (82.4–100%), PB1 (95.4–100%), PA (87.4–100%), NP (96.3–100%), M (96.9–100%), and NS (95.2–100%) formed 3, 5, 4, 3, 4, 3, 3, and 4 groups, respectively. Notably, the NA gene exhibited the highest diversity with 5 distinct groups, while the PB2, PA, and NS genes demonstrated moderate diversity with 4 groups each. Conversely, the HA, PB1, NP, and M genes showed the lowest diversity, with 3 groups each (Supplementary Figure S1). Based on this nucleotide diversity, the H3N2 CIVs were categorized into 15 genotypes (Figure 1B; Supplementary Table S1). As illustrated in Figure 1, H3N2 CIVs have diversified into 15 genotypes since 2006 (Supplementary Table S1). All four H3N2 CIVs isolated in this study were classified under Genotype (G) 15, which has emerged as the predominant genotype since approximately 2017 (Figure 1; Supplementary Table S1). Notably, H3N2 CIVs in China and the United States exhibited similar evolutionary patterns, with viruses from both countries belonging to the same groups, and no distinct Eurasian or North American lineages were observed.

### 3.2 Molecular characterization of H3N2 CIVs

Analysis of the amino acid sequences of the four H3N2 CIVs revealed a conserved monobasic amino acid (R) at the HA cleavage site, indicating their low pathogenicity. However, multiple substitutions at various sites within different proteins are critical for receptor binding, pathogenicity, and host adaptation of AIVs. As shown in Table 1, analysis of HA proteins identified that all four H3N2 isolates contain HA-G146S and HA-N188D mutations, which enhance receptor binding affinity of H3N2 CIVs toward α2,6-linked sialic acids and improve the thermostability of HA (Chen et al., 2023). One virus, CN/HLJ/2/19, harbors G16S in the signal peptide of HA, which enhances acid stability (Chen et al., 2023). No typical mammalian adaptive mutations, such as PB2-E627K and PB2-D701N, were detected in the polymerase proteins. Notably, PB2-292 T, which is common in seasonal human H1N1 and H3N2 viruses (Neumann et al., 2014; Kong et al., 2019); PB2-G590S, PB2-S714I, and PB1-D154G, which enhance polymerase activity and viral replication in mammals, were observed in all four H3N2 CIVs (Zhang et al., 2012; Czudai-Matwich et al., 2014; Chen et al., 2023). Another mutation, NP-R293K, dominant in human viruses, was found in all four viruses (Chen and Shih, 2009). In other proteins, we found only one mammalian adaptive signature, NS1-42S, associated with the pathogenicity of H5 and H1 viruses in mammals (Jiao et al., 2008). In addition, drug resistance mutations, NA-H157Y, and M2-V27I (Monto et al., 2006; Musharrafieh et al., 2018), emerged in all four H3N2 CIVs. Collectively, these data suggest that H3N2 CIVs have evolved to be more mammalian adaptive.

**Table 1:** Key molecular markers of H3 viruses in this study.

No.	Virus name	Amino acid^&^													
		HA				PB2			PB1	NP		NS1	NA	M2	
		16*	146	188	223	292	590	714	154	109	293	42	157	27	31
1	A/canine/Guangdong/1/2006^#^	G	G	N	V	I	G	S	G	V	R	S	H	V	S
2	CN/HLJ/1/18	G	S	D	V	T	S	I	D	V	K	S	Y	I	S
3	CN/HLJ/1/19	G	S	D	V	T	S	I	D	V	K	S	Y	I	S
4	CN/HLJ/2/19	S	S	D	V	T	S	I	D	V	K	S	Y	I	S
5	CN/HLJ/3/19	G	S	D	V	T	S	I	D	V	K	S	Y	I	S

^#^The first H3N2 CIV isolated. ^&^Only the amino acids that confer mammalian adaptation or drug resistance are shown. ^*^Position in the HA signal peptide.

### 3.3 Genetic analysis of HA-V223I in recent H3N2 CIVs

**Figure 2:**
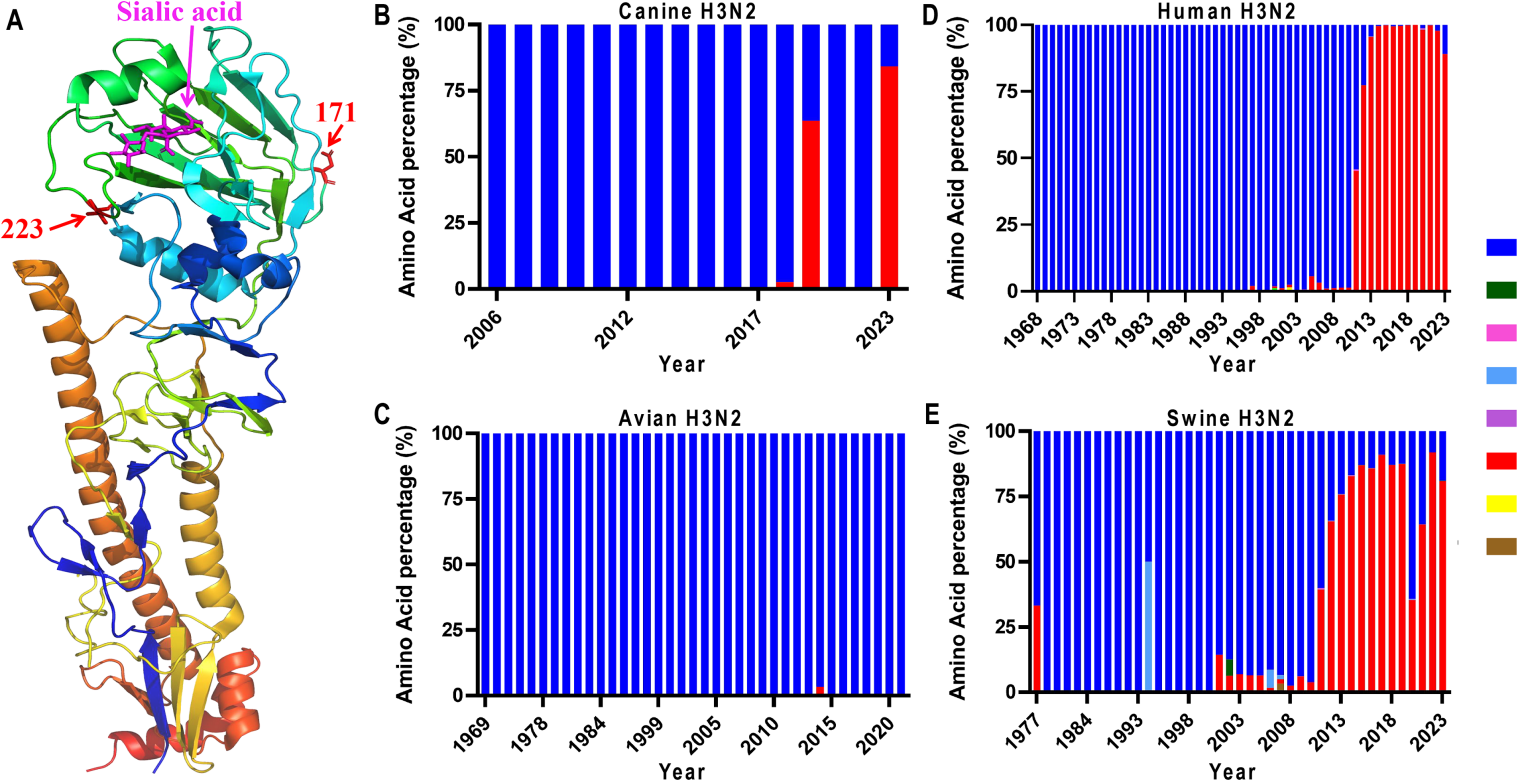
Amino acid position 223 in the 3D structure of HA (PDB: 6aot) **(A)**, and mutation dynamics in HAs from canine **(B)**, avian **(C)**, human **(D)**, and swine **(E)** H3N2 viruses. The HA sequences of these H3N2 viruses were downloaded from the National Center for Biotechnology Information (GenBank).

In addition to the observed adaptive mutations identified in our four isolates, the comprehensive phylogenetic analysis of H3N2 HA proteins also identified two mutations, HA-N171T and HA-V223I, which are dominant in 2023 H3N2 isolates (Figure 2; Supplementary Table S2). According to the structural analysis (Figure 2A), we noticed that only position 223 is located close to the receptor-binding domain of the globular head of HA, suggesting its role in host adaptation. Therefore, we analyzed the amino acid polymorphism of this position. As shown in Figure 2B, HA-V223I appeared in canine H3N2 in 2018 and became dominant in 2023 (Figure 2B). In avian H3N2, position 223 is highly conserved (Figure 2C), with only 2 viruses harboring HA-223I among all viruses analyzed (data not shown). Interestingly, in human and swine H3N2 viruses, we found that HA-V223I surged in 2011 and is now dominant in naturally circulating viruses (Figures 2D,E). These results suggest that HA-V223I may play an important role in host adaptation.

### 3.4 Receptor binding properties

**Figure 3:**
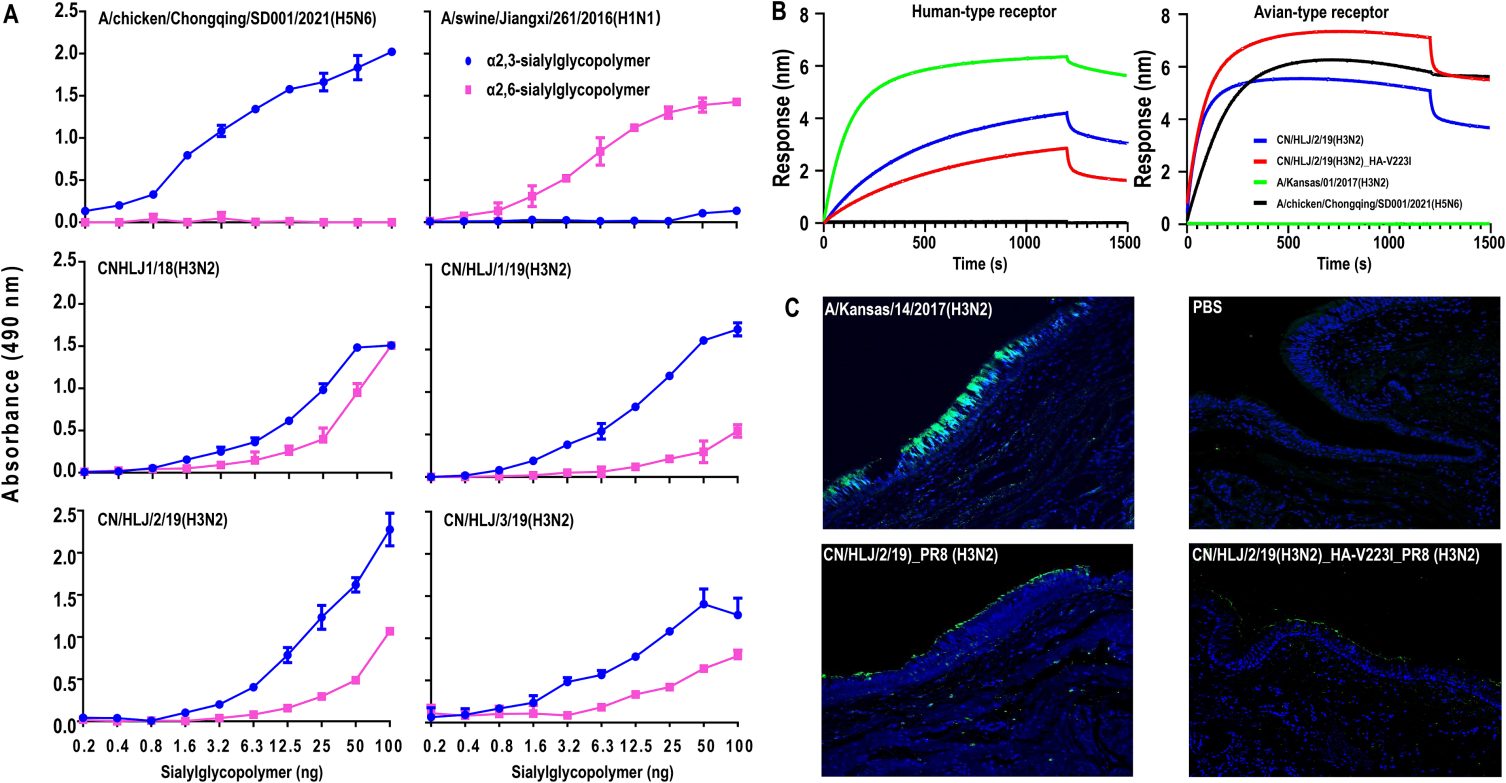
Receptor-Binding Analysis of H3N2 CIVs by using solid-phase binding assay **(A)**, Biolayer interferometry **(B)**, and bindind affnity to human treaches section **(C)**. In the solid-phase binding assay, preferences for avian-type and human-type receptors were assessed using α2,3-sialylglycopolymer (blue) and α2,6-sialylglycopolymer (pink), respectively, conducted in triplicate with avian and swine influenza viruses as controls. The biolayer interferometry assay utilized the Octet Red 96 system to analyze receptor interaction with two biotinylated glycans, 3SLN (avian-type) and 6SLN (human-type), the indicated viruses were analyzed at 30°C for 1, 500 s. For the human tracheal tissue staining, sections were treated with the viruses followed by relevant polyclonal and FITC-labeled secondary antibodies and DAPI dye (blue), where green staining indicated successful virus binding. These methodologies collectively provided detailed insights into the receptor-binding dynamics of H3N2 CIVs.

The H3N2 CIVs have gradually acquired critical mutations in HA that may alter their receptor-binding preference. Therefore, we evaluated the receptor binding affinity of the four wild-type H3N2 viruses using a solid-phase binding assay. As depicted in Figure 3A, the control viruses, A/chicken/Chongqing/SD001/2021 (H5N6) and A/swine/Jiangxi/261/2016 (H1N1), demonstrated a preference for α2,3-sialylglycopolymer (avian-type receptors) and α2,6-sialylglycopolymer (human-type receptors), respectively. All the tested viruses not only bind avian-type receptors but also exhibit an affinity for human-type receptors. These findings suggest that the four H3N2 CIVs possess the potential to infect humans.

The HA-V223I, which is dominant in human and swine H3N2 viruses but highly absent in avian H3N2 viruses, may play an important role in canine adaptation of H3N2 CIVs. To get insights into HA-V223I on the receptor-binding preference in more detail, we selected one representative H3N2 virus, CN/HLJ/2/19, and rescued two H3N2 viruses with or without HA-V223I, CN/HLJ/2/19_PR8 and CN/HLJ/2/19_HA-V223I_PR8, with the surface genes from CN/HLJ/2/19 and the backbone of A/Puerto Rico/8/34 (H1N1; PR8). Then, the receptor-binding preference of the indicated viruses was analyzed by using biolayer interferometry with two synthesized receptor analogs. Two viruses, A/Kansas/14/2017(H3N2) and A/chicken/Chongqing/SD001/2021(H5N6), which preferentially bind to human-type receptors and avian-type receptors, respectively, were set as controls. As shown in Figure 3B, CN/HLJ/2/19_PR8 showed a higher binding affinity toward human-type receptors than CN/HLJ/2/19_HA-V223I_PR8, while CN/ HLJ/2/19_HA-V223I_PR8 exhibited a lower binding affinity toward avian-type receptors than CN/ HLJ/2/19_PR8. These data demonstrate that HA-V223I enhances the binding affinity of H3N2 CIVs to avian-type receptors rather than to human-type receptors.

To further investigate whether the isolated H3N2 CIVs with or without HA-V223I could bind to human-type receptors, sections of normal human tracheal tissues, which exclusively express α2,3-linked sialic acids (Kumlin et al., 2008), were used to analyze attachment patterns. A high affinity was observed for the human H3N2 control virus, A/Kansas/01/2017, whereas no signal was detected for the negative control. Consistent with the results from the solid-phase binding assay, CN/HLJ/2/19_PR8 (H3N2) demonstrated attachment to human tracheal tissues. Similarly, the CN/HLJ/2/19_HA-V223I_PR8 (H3N2) virus also exhibited binding to human tracheal tissues, albeit with a lower affinity compared to CN/HLJ/2/19_PR8 (H3N2) (Figure 3C). These findings strongly suggest that recent H3N2 CIVs have the capacity to bind to human-type receptors, thus posing a potential infection risk to humans.

### 3.5 Role of HA-V223I on thermal stability of H3N2 CIV

**Figure 4:**
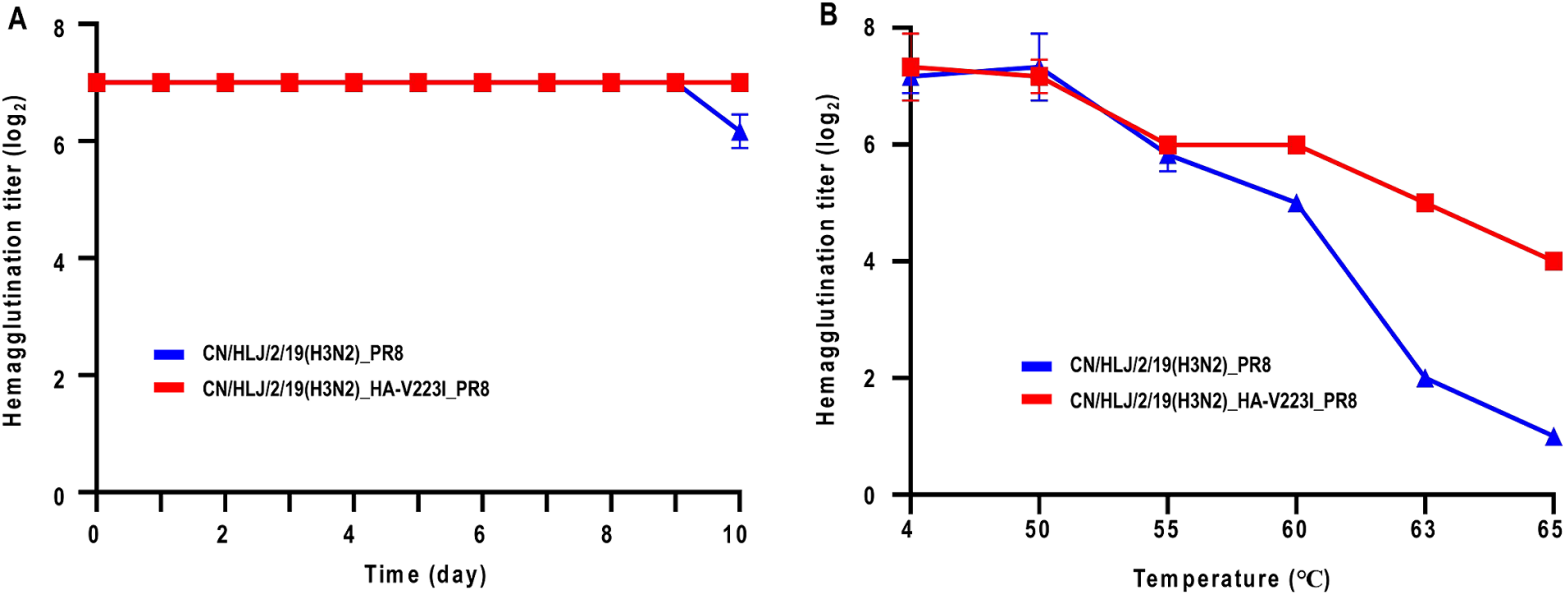
Effect of Heat Treatment on Hemagglutination Activity of H3N2 CIVs at different temperatures. **(A)** Thermal stability of H3N2 CIVs at room temperature for 10 days, or **(B)** at different temperatures for 30 min. Aliquots of viruses containing 128 HA units were incubated at the indicated temperature for the indicated time periods, and the hemagglutination titers of the heat-treated samples were then assessed using a hemagglutination assay with 0.5% chicken red blood cells. Each data point represents the mean ± standard deviation from three replicate experiments.

Besides the receptor-binding preference, thermal stability is another critical factor influencing the transmission of influenza A viruses (Imai et al., 2012; Linster et al., 2014). First, we analyzed the thermal stability at room temperature over a period of 10 days. As shown in Figure 4A, the stability of H3N2 CIV with or without the HA-V223I mutation were relatively stable during the 10-day observation period, with CN/HLJ/2/19(H3N2)_HA-V223I_PR8 being more stable than CN/HLJ/2/19(H3N2)_PR8 on day 10. To confirm the difference in thermal stability, we next assessed the stability of H3N2 CIV with or without the HA-V223I mutation under heat treatment for 30 min at various temperatures (Figure 4B). As depicted in Figure 4, CN/HLJ/2/19(H3N2)_HA-V223I_PR8 exhibited higher thermal stability than CN/HLJ/2/19(H3N2)_PR8 when subjected to heat treatment. These findings suggest that H3N2 CIVs that carry the HA-V223I mutation may be better able to survive under adverse environmental conditions.

### 3.6 Antigenicity analysis

Immunity against H3N2 CIVs is pivotal in preventing cross-species transmission from dogs to humans. To comprehend the antigenic properties of H3N2 CIVs, Hemagglutination Inhibition (HI) assays were conducted using antisera generated against viruses isolated from humans and canines. Antisera against three human viruses and four canine viruses were generated in ferrets and chickens, respectively. As presented in Table 2, antisera against H3N2 human viruses exhibited robust cross-reactivity with the tested human viruses, albeit with HI titers against heterologous viruses 2–4-fold lower than those against their homologous viruses. Intriguingly, none of the antisera generated against human viruses exhibited reactivity with CIVs, with all HI titers below the test limit. Interestingly, antisera generated with H3N2 CIVs not only exhibited strong cross-reactivity with CIVs but also demonstrated significant cross-reactivity with human H3N2 viruses. However, HI titers against heterologous viruses were 2–8-fold lower than those against their homologous viruses. In addition, since human H3N2 viruses preferentially bind human-type receptors and the HI assay is usually analyzed with guinea pig red blood cells according to the Worldwide Influenza Center lab, we also analyzed the antigenicity with guinea pig red blood cells. The data indicated that the reaction pattern was similar to that analyzed with chicken red blood cells (Supplementary Table S3). Taken together, these data indicate that individuals immunized with human H3N2 vaccines may not be fully protected against the cross-transmission of canine H3N2 viruses from dogs to humans.

**Table 2:** Antigenic analysis of H3N2 viruses.

Viruses	Cross-reactive HI antibody titers of antiserum against different H3N2 influenza viruses^a^						
	A/Texas /50/2012^b^	A/Switzerland /9715293/2013	A/Kansas/14/2017	CN/HLJ/1/18^c^	CN/HLJ/1/19	CN/HLJ/2/19	CN/HLJ/3/19
A/Texas /50/2012	**10,240**	2,560	2,560	512	1,024	1,024	1,024
A/Switzerland /9715293/2013	2,560	**5,120**	1,280	256	512	1,024	1,024
A/Kansas/14/2017	2,560	2,560	**1,280**	128	128	128	256
CN/HLJ/1/18	<20	20	<20	**1,024**	512	512	512
CN/HLJ/1/19	<20	<20	<20	1,024	**1,024**	512	1,024
CN/HLJ/2/19	20	20	<20	512	1,024	**1,024**	1,024
CN/HLJ/3/19	20	20	<20	256	512	1,024	**1,024**

^a^Homologous titers are highlighted in bold. ^b^Antisera against H3N2 human and avian viruses were generated in ferrets. ^c^Antisera against H3N2 canine viruses were generated in chickens.

## 4 Discussion

H3N2 CIV has been circulating in China for over a decade and is frequently isolated from dogs (Wasik et al., 2021; Chen et al., 2024), highlighting its adaptation to the most important companion animal. This raises concerns about dogs serving as potential intermediate hosts for the cross-species transmission of H3N2 CIVs from dogs to humans. In this study, we conducted a comprehensive analysis of the genetic and biological characteristics of four H3N2 CIVs isolated in China between 2018 and 2020. Our findings revealed that H3N2 CIVs have undergone substantial evolutionary changes, forming 15 distinct genotypes over time, with G15 emerging as the dominant genotype worldwide, indicating the establishment of a stable lineage in dogs. Molecular characterization unveiled the acquisition of numerous mammalian adaptive mutations, including HA-W222L, HA-G146S, HA-N188D, PB2-I292T, PB2-G590S, PB2-S714I, PB1-D154G, and NP-R293K. Biological experiments demonstrated that the four H3N2 CIVs have evolved to exhibit increased affinity toward α2,6-linked sialic acids and have gained the ability to attach to human tracheal tissues, thus highlighting their potential threat to human health. Interestingly, analysis of recent H3N2 CIVs identified a new host adaptive mutation HA-V223I. Although HA-V223I decreases the receptor-binding preference to human-type receptors, it increases the thermal stability of H3N2 viruses, indicating its easier survival in harsh environments. Therefore, the accumulation of HA-V223I did not downgrade the threat of H3N2 CIVs to human health.

The acquisition of human-type receptor binding affinity is a critical indicator of the potential for influenza viruses to cause pandemics in humans. For instance, the 2013 H7N9 outbreak, which resulted in over 1,500 human infections, was characterized by the virus’s ability to bind to both avian-type and human-type receptors (Shi et al., 2023). Research by Xiong et al. identified two mutations, G186V and G226L, that enhanced the H7N9 virus’s affinity for human receptors (Xiong et al., 2013). Similarly, the HA-G226S mutation has been linked to increase binding affinity in H3N8 avian influenza viruses (AIVs), which were associated with human infections in 2022 and 2023 (Cheng et al., 2022; Cui et al., 2023). In this study, analysis of HA proteins revealed that all H3N2 CIVs in Genotypes 14 and 15 have acquired the HA-G146S mutation (Figure 1). This mutation leads to a shift from recognition solely of avian-type receptors to binding to both avian-type and human-type receptors (Chen et al., 2023). Our solid-phase binding assay, along with attachment analysis, confirmed their binding affinity toward human-type receptors.

The changes in receptor binding affinities to avian-type receptors and human-type receptors are frequently observed in multiple influenza subtypes, such as H7N9 and H10N3. For H7N9 viruses, they initially bind to both avian and human-type receptors. However, after circulating in chickens for 5 years, they exhibit a gradual loss in their ability to bind to human-type receptors, while simultaneously developing a higher affinity for avian-type receptors (Yin et al., 2021), which is caused by the L226Q mutation in HA. In the case of H10N3 viruses, different mutations influence receptor affinity; the HA-G228S mutation increases affinity for human-type receptors, whereas HA-Q222R reduces it (Zhang et al., 2023). In our study, the genetic analysis of canine H3N2 HAs from various species identified HA-V223I as a mammalian adaptive mutation (Figure 2). However, there was no increase in affinity for human-type receptors. Instead, HA-V223I provided greater thermal stability, potentially enhancing the virus’s survivability in environmental conditions. Notably, Song et al. (2008) reported that the canine upper and lower respiratory tract epithelium mainly displays α-2,3 sialic acid receptors. It is reasonable that H3N2 viruses may gradually evolve to increase binding affinity to avian-type receptors (Song et al., 2008). Besides receptor binding preference, thermal stability is a crucial factor influencing virus transmission. For example, Imai et al. reported that the HA-N158D mutation increases the thermal stability of an H5N1 virus, facilitating respiratory transmission among ferrets (Imai et al., 2012). Similarly, Herfst et al. (2012) found that the HA-H110Y mutation, present in a ferret-transmissible virus, enhances the virus’s thermal stability (Linster et al., 2014). These observations collectively suggest that higher thermal stability may allow viruses to survive longer under harsh environmental conditions, thereby potentially improving their ability to transmit. This correlation between thermal stability and transmission highlights the complex dynamics of virus evolution and adaptation in different hosts and environments.

In addition to receptor-binding preference, mutations in other proteins and reassortment play critical roles in the mammalian adaptation of influenza viruses. Two well-known mutations, PB2-E627K and PB2-D701N, frequently identified in mammalian-adapted H5N1 and H7N9 isolates (Hatta et al., 2001; Li et al., 2005), were not found in H3N2 CIVs. The gradual mammalian adaptation occurs through mutations in other proteins. PB2-I292V has been reported to enhance the polymerase activity of the H7N9 virus (Kong et al., 2019), while PB2-I292T has been suggested to facilitate the adaptation of human H1N1 and H3N2 viruses (Miotto et al., 2008). Additionally, PB1-D154G has been demonstrated to increase the polymerase activity of CIVs. Currently, both PB2-I292T and PB1-D154G are prevalent in H3N2 CIVs. Reassortment is another factor that can significantly impact the polymerase activity of influenza viruses in human cells. Bhat et al. found that acquiring the PA gene from an H9N2 virus markedly increased the polymerase activity of an H7N9 virus in human cells. Similarly, Hao et al. demonstrated that the PA gene from an H9N2 virus could enhance the polymerase activity and pathogenicity of an H5N1 virus in mice (Hao et al., 2016). Considering a previous finding that reassortment between H3N2 CIV and H9N2 AIV did occur (Lee et al., 2016), the possibility of a canine H3N2 reassortant with the potential for cross-species transmission is conceivable.

In this study, we conducted a comprehensive analysis to understand the potential threat posed by recent isolates of H3N2 CIVs. Our data indicate that these recent H3N2 CIVs have acquired multiple mammalian adaptive mutations and have gained affinity for human-type receptors, suggesting a potential threat to human health. Therefore, close surveillance and monitoring of H3N2 CIVs in dogs should be of the highest priority.

## Data availability statement

The original contributions presented in the study are included in the article/Supplementary material, further inquiries can be directed to the corresponding author/s.

## Author contributions

LL: Conceptualization, Formal analysis, Investigation, Supervision, Visualization, Writing – original draft, Writing – review & editing. FW: Data curation, Validation, Visualization, Writing – original draft, Conceptualization, Resources, Supervision, Writing – review & editing. YW: Data curation, Formal analysis, Investigation, Methodology, Validation, Visualization, Writing – original draft. WM: Data curation, Formal analysis, Investigation, Methodology, Validation, Visualization, Writing – original draft. YZ: Data curation, Formal analysis, Investigation, Validation, Visualization, Writing – original draft, Methodology. LC: Data curation, Formal analysis, Investigation, Validation, Visualization, Writing – original draft. DW: Data curation, Formal analysis, Methodology, Validation, Visualization, Writing – original draft, Investigation. GD: Data curation, Formal analysis, Methodology, Supervision, Validation, Visualization, Writing – original draft. JS: Investigation, Methodology, Supervision, Validation, Visualization, Writing – original draft, Data curation, Formal analysis, Funding acquisition. HC: Conceptualization, Supervision, Validation, Visualization, Writing – original draft, Writing – review & editing, Investigation, Methodology. HK: Conceptualization, Data curation, Investigation, Methodology, Visualization, Writing – original draft, Writing – review & editing.

## Footnotes

^1^https://platform.epicov.org/, IDs: EPI_ISL_18961353–55, and EPI_ISL_18961022.

^2^ https://www.ncbi.nlm.nih.gov/genbank/

^3^ https://platform.epicov.org/epi3/start

^4^ https://www.bioaitech.com/
