# Supplementary material for: The V223I substitution in hemagglutinin reduces the binding affinity to human-type receptors while enhancing the thermal stability of the H3N2 canine influenza virus
Source: Front Microbiol. 2024 Jul 22;15:1442163. doi: 10.3389/fmicb.2024.1442163 (PMC11299061; doi:10.3389/fmicb.2024.1442163)
Supplement: Supplementary file 4 [file Data_Sheet_1.PDF]

A

1

2

3

4

5

EPI_ISL_17693234_A_canine_Minnesota_012684_2023_HA
EPI_ISL_18750853_A_canine_Texas_CVM_06880321_2023_HA
EPI_ISL_18751256_A_canine_Texas_CVM_0933631_2023_HA
EPI_ISL_18751275_A_canine_Texas_CVM_0204171_2023_HA
EPI_ISL_18751258_A_canine_Texas_CVM_09336441_2023_HA
EPI_ISL_18751252_A_canine_Texas_CVM_0688041_2023_HA
EPI_ISL_18751257_A_canine_Texas_CVM_0933641_2023_HA
EPI_ISL_18751255_A_canine_Texas_CVM_09336221_2023_HA
EPI_ISL_18751254_A_canine_Texas_CVM_09336121_2023_HA
EPI_ISL_18751276_A_canine_Texas_CVM_0204151_2023_HA
EPI_ISL_18751253_A_canine_Texas_CVM_0688071_2023_HA
EPI_ISL_18752209_A_canine_Texas_CVM_042018_3_2023_HA
EPI_ISL_18750852_A_canine_Texas_CVM_04914311_2023_HA
EPI_ISL_17693235_A_canine_Oklahoma_011242_2023_HA
EPI_ISL_18751259_A_canine_Texas_CVM_09336521_2023_HA
EPI_ISL_18752206_A_canine_Pennsylvania_CVM_037756_1_2023_HA
EPI_ISL_18751277_A_canine_Texas_CVM_0420182_2023_HA
EPI_ISL_18750851_A_canine_Texas_CVM_04914241_2023_HA
EPI_ISL_18750735_A_canine_Texas_CVM_3578541_2022_HA
EPI_ISL_18752207_A_canine_Tennessee_CVM_231361_1_2022_HA
EPI_ISL_18752205_A_canine_Alabama_CVM_194456_1_2022_HA
EPI_ISL_18750734_A_canine_Pennsylvania_CVM_985419_2023_HA
EPI_ISL_18751260_A_canine_Virginia_CVM_0029121_2023_HA
EPI_ISL_18751261_A_canine_West_Virginia_CVM_120508_1_2023_HA
EPI_ISL_4061898_A_canine_Illinois_21_015197_005_2021_HA
EPI_ISL_18690797_A_canine_Beijing_CAU_501_2001_HA
EPI_ISL_18690798_A_canine_Beijing_CAU_510_2010_HA
EPI_ISL_18690800_A_canine_Beijing_CAU_JC2538_2010_HA
EPI_ISL_18690801_A_canine_Beijing_CAU_JC2550_2010_HA
EPI_ISL_18750646_A_canine_California_CVM_32151101_2021_HA
EPI_ISL_18750730_A_canine_Florida_CVM_B1_2021_HA
EPI_ISL_18750726_A_canine_California_CVM_32918141_2021_HA
EPI_ISL_18750723_A_canine_California_CVM_3235751_2021_HA
EPI_ISL_18750729_A_canine_California_CVM_3466381_2021_HA
EPI_ISL_18750727_A_canine_California_CVM_3299021_2021_HA
EPI_ISL_18750728_A_canine_California_CVM_33954291_2021_HA
EPI_ISL_18750724_A_canine_California_CVM_3275921_2021_HA
EPI_ISL_18750647_A_canine_California_CVM_3215141_2021_HA
EPI_ISL_4061925_A_canine_Texas_21_011409_001_2021_HA
CN_HLJ_2_19_H3N2__HA
EPI_ISL_4031576_A_canine_Guangdong_DY1_2019_HA
CN_HLJ_1_19_H3N2__HA
CN_HLJ_1_18_H3N2__HA
EPI_ISL_17277265_A_canine_China_6068_2018_HA
EPI_ISL_17277266_A_canine_China_15001_2019_HA
EPI_ISL_17277337_A_canine_China_15068_2019_HA
EPI_ISL_17277336_A_canine_China_15066_2019_HA
EPI_ISL_17277335_A_canine_China_15027_2019_HA
EPI_ISL_18750107_A_canine_California_CVM_17384101_2019_HA
EPI_ISL_18750108_A_canine_California_CVM_1738411_2019_HA
EPI_ISL_366884_A_canine_China_Shanghai_0315_2019_2019_HA
EPI_ISL_499084_A_canine_Guangdong_1_2018_HA
EPI_ISL_499085_A_canine_Guangdong_2_2018_HA
CN_HLJ_3_19_H3N2__HA
EPI_ISL_502293_A_canine_Ontario_NCFAD_2018_070_10_2018_HA
EPI_ISL_502304_A_canine_Ontario_NCFAD_2018_070_7_2018_HA
EPI_ISL_502305_A_canine_Ontario_NCFAD_2018_070_8_2018_HA
EPI_ISL_502306_A_canine_Ontario_NCFAD_2018_070_9_2018_HA
EPI_ISL_502294_A_canine_Ontario_NCFAD_2018_070_11_2018_HA
EPI_ISL_502295_A_canine_Ontario_NCFAD_2018_070_12_2018_HA
EPI_ISL_502298_A_canine_Ontario_NCFAD_2018_070_15_2018_HA
EPI_ISL_502297_A_canine_Ontario_NCFAD_2018_070_14_2018_HA
EPI_ISL_502299_A_canine_Ontario_NCFAD_2018_070_16_2018_HA
EPI_ISL_502296_A_canine_Ontario_NCFAD_2018_070_13_2018_HA
EPI_ISL_372279_A_Canis_lupus_familiaris_USA_000915_2018_HA
EPI_ISL_372281_A_Canis_lupus_familiaris_USA_007780_2018_HA
EPI_ISL_379495_A_canine_Shanghai_20170721_36_2017_HA
EPI_ISL_379497_A_canine_Shanghai_20170622_7_2017_HA
EPI_ISL_379496_A_canine_Shanghai_20170713_18_2017_HA
EPI_ISL_379513_A_canine_Beijing_20170720_30_2017_HA
EPI_ISL_379518_A_canine_Beijing_20170616_202_2017_HA
EPI_ISL_379529_A_canine_Beijing_20170528_147_2017_HA
EPI_ISL_379530_A_canine_Beijing_20170527_146_2017_HA
EPI_ISL_379536_A_canine_Beijing_20170510_118_2017_HA
EPI_ISL_379514_A_canine_Beijing_20170712_18_2017_HA
EPI_ISL_379531_A_canine_Beijing_20170512_137_2017_HA
EPI_ISL_379532_A_canine_Beijing_20170512_133_2017_HA
EPI_ISL_502287_A_canine_Ontario_NCFAD_2018_064_1_2018_HA
EPI_ISL_502288_A_canine_Ontario_NCFAD_2018_069_1_2018_HA
EPI_ISL_502289_A_canine_Ontario_NCFAD_2018_069_10_2018_HA
EPI_ISL_502291_A_canine_Ontario_NCFAD_2018_069_9_2018_HA
EPI_ISL_502290_A_canine_Ontario_NCFAD_2018_069_6_2018_HA
EPI_ISL_329151_A_canine_Ohio_A82_2017_HA
EPI_ISL_372288_A_Canis_lupus_familiaris_USA_188203_2017_HA
EPI_ISL_372289_A_Canis_lupus_familiaris_USA_188226_2017_HA
EPI_ISL_372287_A_Canis_lupus_familiaris_USA_118191_2017_HA
EPI_ISL_329172_A_canine_Illinois_A37_2017_HA
EPI_ISL_329152_A_canine_Ohio_A91_2017_HA
EPI_ISL_372290_A_Canis_lupus_familiaris_USA_188297_2017_HA
EPI_ISL_379517_A_canine_Beijing_20170618_206_2017_HA
EPI_ISL_379523_A_canine_Beijing_20170610_174_2017_HA
EPI_ISL_379519_A_canine_Beijing_20170616_200_2017_HA
EPI_ISL_379525_A_canine_Beijing_20170610_172_2017_HA
EPI_ISL_379524_A_canine_Beijing_20170610_173_2017_HA
EPI_ISL_379520_A_canine_Beijing_20170615_195_2017_HA
EPI_ISL_379540_A_canine_Beijing_20170417_81_2017_HA
EPI_ISL_379521_A_canine_Beijing_20170614_185_2017_HA
EPI_ISL_329154_A_canine_South_Carolina_132956_2017_HA
EPI_ISL_379493_A_canine_Xi_an_20170601_159_2017_HA
EPI_ISL_379494_A_canine_Xi_an_20170601_157_2017_HA
EPI_ISL_379526_A_canine_Beijing_20170604_164_2017_HA
EPI_ISL_379498_A_canine_Nanjing_20170704_53_2017_HA
EPI_ISL_379499_A_canine_Nanjing_20170620_47_2017_HA
EPI_ISL_379502_A_canine_Nanjing_20170419_12_2017_HA
EPI_ISL_379500_A_canine_Nanjing_20170607_40_2017_HA
EPI_ISL_379501_A_canine_Nanjing_20170515_25_2017_HA
EPI_ISL_379503_A_canine_Nanjing_20170328_7_2017_HA
EPI_ISL_379505_A_canine_Beijing_20161228_9_2016_HA
EPI_ISL_379506_A_canine_Beijing_20161216_8_2016_HA
EPI_ISL_379515_A_canine_Beijing_20170711_16_2017_HA
EPI_ISL_379535_A_canine_Beijing_20170512_120_2017_HA
EPI_ISL_6785379_A_canine_Henan_L03_2018_HA
EPI_ISL_372280_A_Canis_lupus_familiaris_USA_006974_2018_HA
EPI_ISL_502302_A_canine_Ontario_NCFAD_2018_070_4_2018_HA
EPI_ISL_502292_A_canine_Ontario_NCFAD_2018_070_1_2018_HA
EPI_ISL_372293_A_canine_California_BRW002_2018_HA
EPI_ISL_372292_A_Canis_lupus_familiaris_USA_218592_2017_HA
EPI_ISL_502301_A_canine_Ontario_NCFAD_2018_070_3_2018_HA
EPI_ISL_372295_A_canine_Nevada_BRW015_2018_HA
EPI_ISL_502300_A_canine_Ontario_NCFAD_2018_070_2_2018_HA
EPI_ISL_502303_A_canine_Ontario_NCFAD_2018_070_5_2018_HA
EPI_ISL_502286_A_canine_Ontario_NCFAD_2018_001_4_2018_HA
EPI_ISL_372294_A_canine_California_BRW003_2018_HA
EPI_ISL_372282_A_Canis_lupus_familiaris_USA_007781_2018_HA
EPI_ISL_372284_A_Canis_lupus_familiaris_USA_150620_2018_HA
EPI_ISL_372285_A_Canis_lupus_familiaris_USA_152386_2018_HA
EPI_ISL_372297_A_canine_New_Jersey_CRP036_2018_HA
EPI_ISL_372298_A_canine_New_York_CRP034_2018_HA
EPI_ISL_372296_A_canine_Nevada_BRW018_2018_HA
EPI_ISL_17805983_A_dog_Indiana_M16_09378_32_1_2016_HA
EPI_ISL_308898_A_canine_Illinois_20170110_1G4_2016_HA
EPI_ISL_308873_A_canine_Illinois_M16_25374_14_1_2016_HA
EPI_ISL_308888_A_canine_Illinois_M16_16391_2_1_2016_HA
EPI_ISL_329169_A_canine_Illinois_145135_16_3_1_2016_HA
EPI_ISL_308880_A_canine_Illinois_M16_30480_3_1_2016_HA
EPI_ISL_308883_A_canine_Illinois_M17_05782_8_1_2017_HA
EPI_ISL_308889_A_canine_Illinois_M17_05782_1_1_2017_HA
EPI_ISL_308890_A_canine_Illinois_M17_05782_4_1_2017_HA
EPI_ISL_308896_A_canine_Illinois_M17_05782_7_1_2017_HA
EPI_ISL_308887_A_canine_Illinois_M17_05782_11_1_2017_HA
EPI_ISL_308885_A_canine_Pennsylvania_20170411_5_2017_HA
EPI_ISL_329176_A_canine_Indiana_1177_17_1_2017_HA
EPI_ISL_308886_A_canine_Illinois_20170110_1G12_2016_HA
EPI_ISL_329173_A_canine_Indiana_132276_16_1_2_2016_HA
EPI_ISL_329178_A_canine_Indiana_17031_16_2_2016_HA
EPI_ISL_329167_A_canine_Illinois_20170110_1A4_2016_HA
EPI_ISL_308882_A_canine_Illinois_187427_16_7_2016_HA
EPI_ISL_308895_A_canine_Illinois_20170110_2G1_2016_HA
EPI_ISL_308897_A_canine_Illinois_20170110_2F1_2016_HA
EPI_ISL_329175_A_canine_Indiana_093165_16_1_1_2016_HA
EPI_ISL_329183_A_canine_Illinois_091375_16_1_1_2016_HA
EPI_ISL_329174_A_canine_Illinois_10182389_2015_HA
EPI_ISL_190463_A_canine_Illinois_12191_2015_HA
EPI_ISL_329177_A_canine_Illinois_41915_2015_HA
EPI_ISL_234608_A_canine_Illinois_1619144_2015_HA
EPI_ISL_234610_A_canine_Georgia_95391_2015_HA
EPI_ISL_234604_A_canine_Wisconsin_19137_2016_HA
EPI_ISL_284696_A_canine_Illinois_077753_2016_HA
EPI_ISL_329168_A_canine_Illinois_077752_2016_HA
EPI_ISL_308891_A_canine_Illinois_M16_25374_19_1_2016_HA
EPI_ISL_284694_A_canine_Indiana_003018_2016_HA
EPI_ISL_284698_A_canine_Illinois_328292_2015_HA
EPI_ISL_234669_A_canine_Texas_343907_2015_HA
EPI_ISL_284697_A_canine_Illinois_283066_2015_HA
EPI_ISL_329164_A_canine_Illinois_278597_2016_HA
EPI_ISL_234606_A_canine_North_Carolina_109904_2015_HA
EPI_ISL_234609_A_canine_Indiana_96198_2015_HA
EPI_ISL_309184_A_canine_Korea_AS_05_2012_HA
EPI_ISL_309186_A_canine_Korea_AS_08_2012_HA
EPI_ISL_309187_A_canine_Korea_AS_09_2012_HA
EPI_ISL_309182_A_canine_Korea_AS_03_2012_HA
EPI_ISL_309183_A_canine_Korea_AS_04_2012_HA
EPI_ISL_309188_A_canine_Korea_AS_11_2013_HA
KT446475_1_A_canine_Korea_S3001_2015_H3N2__HA
EPI_ISL_309189_A_canine_Korea_AS_14_2013_HA
EPI_ISL_309190_A_canine_Korea_AS_15_2013_HA
EPI_ISL_309185_A_canine_Korea_AS_06_2012_HA
EPI_ISL_129987_A_canine_Korea_KRIBB01_2011_HA
EPI_ISL_138997_A_canine_Korea_CY009_2010_HA
EPI_ISL_279526_A_canine_Korea_CY053_2014_HA
EPI_ISL_308872_A_canine_California_20170411_20_2017_HA
EPI_ISL_328525_A_canine_California_043670_2017_HA
EPI_ISL_379543_A_canine_Beijing_20150122_284_2015_HA
EPI_ISL_379544_A_canine_Beijing_20150120_265_2015_HA
EPI_ISL_285502_A_canine_China_JLM1_2015_HA
EPI_ISL_285503_A_canine_China_JLM2_2015_HA
EPI_ISL_297933_A_canine_China_LG1_2015_HA
EPI_ISL_297934_A_canine_China_LG2_2015_HA
EPI_ISL_379545_A_canine_Beijing_20150118_256_2015_HA
EPI_ISL_379508_A_canine_Beijing_20131207_365_2013_HA
EPI_ISL_379546_A_canine_Beijing_20140110_2_2014_HA
EPI_ISL_379547_A_canine_Beijing_20140103_90_2014_HA
EPI_ISL_379512_A_canine_Beijing_20131112_279_2013_HA
EPI_ISL_379510_A_canine_Beijing_20131203_342_2013_HA
EPI_ISL_379511_A_canine_Beijing_20131203_341_2013_HA
EPI_ISL_379509_A_canine_Beijing_20131204_349_2013_HA
EPI_ISL_379507_A_canine_Beijing_20121215_34_2012_HA
EPI_ISL_379504_A_canine_Beijing_20121230_62_2012_HA
EPI_ISL_162440_A_canine_Heilongjiang_L1_2013_HA
EPI_ISL_138948_A_canine_Guangdong_05_2011_HA
EPI_ISL_151145_A_canine_Guangdong_12_2012_HA
EPI_ISL_142881_A_canine_Guangdong_3_2011_HA
EPI_ISL_329148_A_canine_Guangdong_01_2014_HA
EPI_ISL_329149_A_canine_Guangdong_02_2014_HA
EPI_ISL_329147_A_canine_Guangdong_04_2013_HA
EPI_ISL_329146_A_canine_Guangdong_03_2013_HA
EPI_ISL_128938_A_canine_Guangdong_2_2011_HA
EPI_ISL_93813_A_canine_Jiangsu_05_2010_HA
EPI_ISL_93814_A_canine_Jiangsu_04_2010_HA
EPI_ISL_93815_A_canine_Jiangsu_03_2010_HA
EPI_ISL_93817_A_canine_Jiangsu_01_2009_HA
EPI_ISL_93816_A_canine_Jiangsu_02_2010_HA
EPI_ISL_93812_A_canine_Jiangsu_06_2010_HA
EPI_ISL_309181_A_canine_Korea_AS_01_2009_HA
EPI_ISL_127502_A_canine_Korea_01_2007_HA
EPI_ISL_80506_A_canine_Guangdong_2_2006_HA
EPI_ISL_80508_A_canine_Guangdong_1_2007_HA
EPI_ISL_80505_A_canine_Guangdong_1_2006_HA

0.006

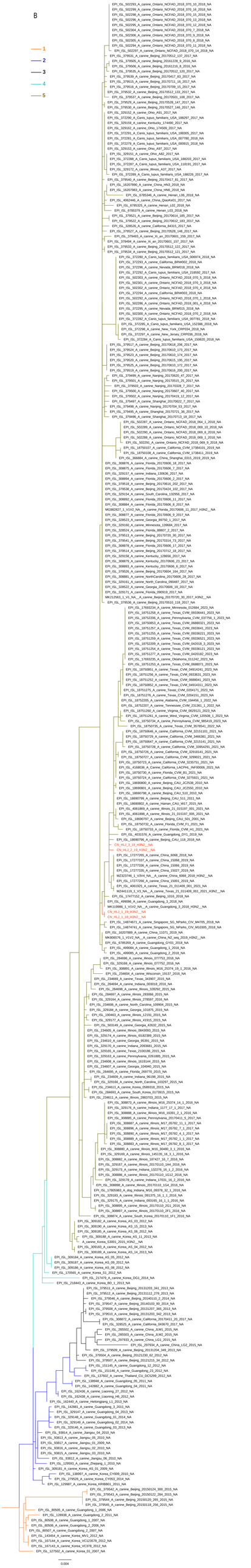
B
1
2
3
4
5
EPI_ISL_502293_A_canine_Ontario_NCFAD_2018_070_1d_2018_NA
EPI_ISL_502299_A_canine_Ontario_NCFAD_2018_070_1d_2018_NA
EPI_ISL_502298_A_canine_Ontario_NCFAD_2018_070_1d_2018_NA
EPI_ISL_502296_A_canine_Ontario_NCFAD_2018_070_1d_2018_NA
EPI_ISL_502304_A_canine_Ontario_NCFAD_2018_070_7_2018_NA
EPI_ISL_502305_A_canine_Ontario_NCFAD_2018_070_8_2018_NA
EPI_ISL_502294_A_canine_Ontario_NCFAD_2018_070_1d_2018_NA
EPI_ISL_502297_A_canine_Ontario_NCFAD_2018_070_1d_2018_NA
EPI_ISL_379531_A_canine_Illinois_20170512_137_2017_NA
EPI_ISL_379505_A_canine_Beijing_20161228_9_2016_NA
EPI_ISL_379506_A_canine_Beijing_20161216_8_2016_NA
EPI_ISL_379535_A_canine_Beijing_20170512_120_2017_NA
EPI_ISL_379539_A_canine_Beijing_20170417_83_2017_NA
EPI_ISL_379515_A_canine_Beijing_20170711_16_2017_NA
EPI_ISL_379516_A_canine_Beijing_20170709_15_2017_NA
EPI_ISL_379522_A_canine_Beijing_20170523_146_2017_NA
EPI_ISL_379537_A_canine_Beijing_20170501_108_2017_NA
EPI_ISL_379529_A_canine_Beijing_20170528_147_2017_NA
EPI_ISL_379530_A_canine_Beijing_20170527_146_2017_NA
EPI_ISL_329152_A_canine_Ohio_A81_2017_NA
EPI_ISL_372290_A_Canis_lupus_familiaris_USA_188297_2017_NA
EPI_ISL_329159_A_canine_Kentucky_174490_2017_NA
EPI_ISL_329162_A_canine_Ohio_174509_2017_NA
EPI_ISL_372291_A_Canis_lupus_familiaris_USA_188305_2017_NA
EPI_ISL_372281_A_Canis_lupus_familiaris_USA_007780_2018_NA
EPI_ISL_372279_A_Canis_lupus_familiaris_USA_000915_2018_NA
EPI_ISL_329153_A_canine_Ohio_A97_2017_NA
EPI_ISL_329151_A_canine_Ohio_A92_2017_NA
EPI_ISL_372288_A_Canis_lupus_familiaris_USA_188203_2017_NA
EPI_ISL_372287_A_Canis_lupus_familiaris_USA_118191_2017_NA
EPI_ISL_329172_A_canine_Illinois_A97_2017_NA
EPI_ISL_328526_A_canine_California_84315_2017_NA
EPI_ISL_379527_A_canine_Beijing_20170526_148_2017_NA
EPI_ISL_379483_A_canine_Xi_an_20170501_159_2017_NA
EPI_ISL_379494_A_canine_Xi_an_20170601_157_2017_NA
EPI_ISL_379533_A_canine_Beijing_20170512_122_2017_NA
EPI_ISL_379534_A_canine_Beijing_20170512_121_2017_NA
EPI_ISL_372280_A_Canis_lupus_familiaris_USA_006974_2018_NA
EPI_ISL_372293_A_canine_California_BRW002_2018_NA
EPI_ISL_372296_A_canine_Nevada_BRW018_2018_NA
EPI_ISL_372282_A_Canis_lupus_familiaris_USA_218560_2017_NA
EPI_ISL_502303_A_canine_Ontario_NCFAD_2018_070_5_2018_NA
EPI_ISL_502301_A_canine_Ontario_NCFAD_2018_070_3_2018_NA
EPI_ISL_502302_A_canine_Ontario_NCFAD_2018_070_4_2018_NA
EPI_ISL_372294_A_canine_California_BRW003_2018_NA
EPI_ISL_502292_A_canine_Ontario_NCFAD_2018_070_1_2018_NA
EPI_ISL_502298_A_canine_Ontario_NCFAD_2018_001_6_2018_NA
EPI_ISL_372295_A_canine_Nevada_BRW015_2018_NA
EPI_ISL_502300_A_canine_Ontario_NCFAD_2018_070_2_2018_NA
EPI_ISL_372282_A_Canis_lupus_familiaris_USA_007781_2018_NA
EPI_ISL_372285_A_Canis_lupus_familiaris_USA_152386_2018_NA
EPI_ISL_372286_A_canine_New_York_CIIP034_2018_NA
EPI_ISL_372297_A_canine_New_Jersey_CRP036_2018_NA
EPI_ISL_372284_A_Canis_lupus_familiaris_USA_150928_2018_NA
EPI_ISL_379517_A_canine_Beijing_20170618_206_2017_NA
EPI_ISL_379524_A_canine_Beijing_20170610_173_2017_NA
EPI_ISL_379523_A_canine_Beijing_20170610_174_2017_NA
EPI_ISL_379520_A_canine_Beijing_20170615_195_2017_NA
EPI_ISL_379525_A_canine_Beijing_20170610_172_2017_NA
EPI_ISL_379519_A_canine_Beijing_20170616_200_2017_NA
EPI_ISL_379499_A_canine_Nanjing_20170620_47_2017_NA
EPI_ISL_379501_A_canine_Nanjing_20170618_25_2017_NA
EPI_ISL_379503_A_canine_Nanjing_20170328_7_2017_NA
EPI_ISL_379498_A_canine_Nanjing_20170704_53_2017_NA
EPI_ISL_379495_A_canine_Shanghai_20170721_36_2017_NA
EPI_ISL_379496_A_canine_Shanghai_20170713_18_2017_NA
EPI_ISL_502287_A_canine_Ontario_NCFAD_2018_064_1_2018_NA
EPI_ISL_502289_A_canine_Ontario_NCFAD_2018_069_10_2018_NA
EPI_ISL_502290_A_canine_Ontario_NCFAD_2018_069_8_2018_NA
EPI_ISL_502288_A_canine_Ontario_NCFAD_2018_069_1_2018_NA
EPI_ISL_502291_A_canine_Ontario_NCFAD_2018_069_9_2018_NA
EPI_ISL_18750107_A_canine_California_CVM_173841011_2019_NA
EPI_ISL_18750108_A_canine_California_CVM_173841011_2019_NA
EPI_ISL_368864_A_canine_China_Shanghai_0315_2019_2019_NA
EPI_ISL_308876_A_canine_Florida_20170608_18_2017_NA
EPI_ISL_308875_A_canine_Florida_20170606_2_2017_NA
EPI_ISL_329157_A_canine_Indiana_130636_2017_NA
EPI_ISL_308894_A_canine_Florida_20170609_2_2017_NA
EPI_ISL_379518_A_canine_Beijing_20170624_101_2017_NA
EPI_ISL_379538_A_canine_Beijing_20170624_102_2017_NA
EPI_ISL_329154_A_canine_South_Carolina_132956_2017_NA
EPI_ISL_308892_A_canine_Florida_20170608_11_2017_NA
EPI_ISL_308884_A_canine_Florida_20170608_8_2017_NA
MG982087.1_V1V2_NA_A_canine_Florida_20170606_11_2017_H3N2_NA
EPI_ISL_308877_A_canine_Florida_20170608_9_2017_NA
EPI_ISL_328523_A_canine_Georgia_89750_1_2017_NA
EPI_ISL_329160_A_canine_Minnesota_139584_2017_NA
EPI_ISL_328524_A_canine_Florida_88807_2_2017_NA
EPI_ISL_379513_A_canine_Beijing_20170703_30_2017_NA
EPI_ISL_379541_A_canine_Beijing_20170314_73_2017_NA
EPI_ISL_308878_A_canine_Florida_20170608_17_2017_NA
EPI_ISL_379514_A_canine_Beijing_20170712_31_2017_NA
EPI_ISL_329158_A_canine_Kentucky_128656_2017_NA
EPI_ISL_308879_A_canine_Kentucky_20170606_23_2017_NA
EPI_ISL_308893_A_canine_Kentucky_20170606_6_2017_NA
EPI_ISL_379526_A_canine_Beijing_20170604_164_2017_NA
EPI_ISL_308881_A_canine_NorthCarolina_20170608_28_2017_NA
EPI_ISL_329161_A_canine_North_Carolina_090487_2017_NA
EPI_ISL_328522_A_canine_Georgia_20170606_30_2017_NA
EPI_ISL_329171_A_canine_Florida_090919_2017_NA
MK212563.1_V1_NA_A_canine_Beijing_20170720_30_2017_H3N2_NA
EPI_ISL_379528_A_canine_Beijing_20170710_118_2017_NA
EPI_ISL_17693234_A_canine_Minnesota_012684_2023_NA
EPI_ISL_18751258_A_canine_Texas_CVM_20180402_2023_NA
EPI_ISL_18752206_A_canine_Pennsylvania_CVM_037756_1_2023_NA
EPI_ISL_18750553_A_canine_Texas_CVM_06880321_2023_NA
EPI_ISL_18751257_A_canine_Texas_CVM_09353641_2023_NA
EPI_ISL_18751255_A_canine_Texas_CVM_09336221_2023_NA
EPI_ISL_18751259_A_canine_Texas_CVM_09336521_2023_NA
EPI_ISL_18752209_A_canine_Texas_CVM_042018_3_2023_NA
EPI_ISL_18751254_A_canine_Texas_CVM_09336121_2023_NA
EPI_ISL_18751277_A_canine_Texas_CVM_04202182_2023_NA
EPI_ISL_17693235_A_canine_Oklahoma_011242_2023_NA
EPI_ISL_18751253_A_canine_Texas_CVM_09888721_2023_NA
EPI_ISL_18750851_A_canine_Texas_CVM_04914241_2023_NA
EPI_ISL_18751256_A_canine_Texas_CVM_09336321_2023_NA
EPI_ISL_18751252_A_canine_Texas_CVM_06888041_2023_NA
EPI_ISL_18750852_A_canine_Texas_CVM_04914311_2023_NA
EPI_ISL_18751275_A_canine_Texas_CVM_02041721_2023_NA
EPI_ISL_18751279_A_canine_Texas_CVM_02041511_2023_NA
EPI_ISL_18752205_A_canine_Alabama_CVM_194456_1_2022_NA
EPI_ISL_18752207_A_canine_Tennessee_CVM_231361_1_2022_NA
EPI_ISL_18751290_A_canine_Virginia_CVM_00291221_2023_NA
EPI_ISL_18751261_A_canine_West_Virginia_CVM_120508_1_2023_NA
EPI_ISL_18750713_A_canine_Pennsylvania_CVM_985413_2023_NA
EPI_ISL_18750735_A_canine_Texas_CVM_3578541_2022_NA
EPI_ISL_18750646_A_canine_California_CVM_32151101_2023_NA
EPI_ISL_18750729_A_canine_California_CVM_34663801_2023_NA
EPI_ISL_18750647_A_canine_California_CVM_32151411_2023_NA
EPI_ISL_18750728_A_canine_California_CVM_32914141_2023_NA
EPI_ISL_18750727_A_canine_California_CVM_32908021_2023_NA
EPI_ISL_18750723_A_canine_California_CVM_32357051_2023_NA
EPI_ISL_41608036_A_canine_California_LACPHL_INF00006_2021_NA
EPI_ISL_18750730_A_canine_California_CVM_81_2021_NA
EPI_ISL_18750724_A_canine_California_CVM_3275921_2021_NA
EPI_ISL_18690600_A_canine_Beijing_CAU_CA2538_2010_NA
EPI_ISL_18690601_A_canine_Beijing_CAU_CA2550_2010_NA
EPI_ISL_18690708_A_canine_Beijing_CAU_510_2011_NA
EPI_ISL_18690799_A_canine_Beijing_CAU_511_2011_NA
EPI_ISL_18690802_A_canine_Hainan_CAU_W17_2015_NA
EPI_ISL_4861869_A_canine_Illinois_21_015197_001_2021_NA
EPI_ISL_4861898_A_canine_Illinois_21_015197_005_2021_NA
EPI_ISL_18690797_A_canine_Beijing_CAU_501_2001_NA
EPI_ISL_18750732_A_canine_Florida_CVM_FL1_2021_NA
EPI_ISL_18750733_A_canine_Florida_CVM_H1_2021_NA
EPI_ISL_4031576_A_canine_Guangdong_DY1_2018_NA
EPI_ISL_18690796_A_canine_Beijing_CAU_118_2018_NA
CN_HL2_3_19_H3N2__NA
CN_HL2_2_19_H3N2__NA
EPI_ISL_18750725_A_canine_California_CVM_32918141_2023_NA
EPI_ISL_18750731_A_canine_Texas_21_011409_001_2021_NA
MZ441119.1_V2_NA_A_canine_Texas_21_011409_001_2021_H3N2_NA
EPI_ISL_17477152_A_canine_Beijing_1016_2018_NA
EPI_ISL_499088_A_canine_Guangdong_1_2018_NA
MK119986.1_V1V2_NA_A_canine_Guangdong_3_2018_H3N2_NA
CN_HL2_1_19_H3N2__NA
CN_HL2_1_18_H3N2__NA
EPI_ISL_18749671_A_canine_Singapore_SG_NParks_CV_M4705_2018_NA
EPI_ISL_18749741_A_canine_Singapore_SG_NParks_CV_M10365_2018_NA
EPI_ISL_18207889_A_canine_China_11071_2019_NA
MK806576.1_V1V2_NA_A_canine_China_NZ_two_2018_H3N2__NA
EPI_ISL_6785319_A_canine_Guangdong_DY2_2018_NA
EPI_ISL_499084_A_canine_Guangdong_1_2018_NA
EPI_ISL_499085_A_canine_Guangdong_2_2018_NA
EPI_ISL_284696_A_canine_Illinois_077753_2016_NA
EPI_ISL_329168_A_canine_Illinois_077752_2016_NA
EPI_ISL_308881_A_canine_Illinois_M13_08724_19_1_2016_NA
EPI_ISL_234604_A_canine_Wisconsin_19137_2016_NA
EPI_ISL_234693_A_canine_Texas_3439067_2016_NA
EPI_ISL_284694_A_canine_Indiana_0030818_2016_NA
EPI_ISL_284638_A_canine_Illinois_328292_2015_NA
EPI_ISL_284807_A_canine_Illinois_283066_2015_NA
EPI_ISL_329164_A_canine_Illinois_278597_2016_NA
EPI_ISL_234606_A_canine_North_Carolina_130215_2015_NA
EPI_ISL_329184_A_canine_Georgia_101875_2015_NA
EPI_ISL_190463_A_canine_Illinois_12191_2015_NA
EPI_ISL_329177_A_canine_Illinois_41915_2015_NA
EPI_ISL_503149_A_canine_Georgia_42632_2015_NA
EPI_ISL_234605_A_canine_Illinois_084300I_2015_NA
EPI_ISL_329174_A_canine_Illinois_91823B9_2015_NA
EPI_ISL_234610_A_canine_Georgia_95391_2015_NA
EPI_ISL_329170_A_canine_Indiana_2090881_2015_NA
EPI_ISL_329165_A_canine_Texas_2100186_2015_NA
EPI_ISL_329163_A_canine_Pennsylvania_2091885_2015_NA
EPI_ISL_234608_A_canine_Illinois_16191444_2015_NA
EPI_ISL_234607_A_canine_Georgia_104940_2015_NA
EPI_ISL_284695_A_canine_Florida_269770_2015_NA
EPI_ISL_234609_A_canine_Indiana_96168_2015_NA
EPI_ISL_329166_A_canine_North_Carolina_105207_2015_NA
EPI_ISL_234613_A_canine_Korea_05B9318_2015_NA
EPI_ISL_284693_A_canine_South_Korea_0173915_2015_NA
EPI_ISL_234611_A_canine_Illinois_2883703_2015_NA
EPI_ISL_308873_A_canine_Illinois_M16_25374_14_1_2016_NA
EPI_ISL_329176_A_canine_Indiana_1177_17_1_2017_NA
EPI_ISL_308898_A_canine_Illinois_M16_16924_2_1_2016_NA
EPI_ISL_308885_A_canine_Pennsylvania_20170411_5_2017_NA
EPI_ISL_308887_A_canine_Illinois_M17_05782_11_1_2017_NA
EPI_ISL_308896_A_canine_Illinois_M17_05782_7_1_2017_NA
EPI_ISL_308890_A_canine_Illinois_M17_05782_4_1_2017_NA
EPI_ISL_308889_A_canine_Illinois_M17_05782_1_1_2017_NA
EPI_ISL_308883_A_canine_Illinois_M17_05782_8_1_2017_NA
EPI_ISL_308880_A_canine_Illinois_M16_30480_1_2016_NA
EPI_ISL_329169_A_canine_Illinois_145135_16_3_1_2016_NA
EPI_ISL_308882_A_canine_Illinois_187427_16_7_2016_NA
EPI_ISL_329167_A_canine_Illinois_20170110_1d4_2016_NA
EPI_ISL_329173_A_canine_Indiana_132276_16_1_2_2016_NA
EPI_ISL_308888_A_canine_Illinois_20170110_1G12_2016_NA
EPI_ISL_329178_A_canine_Indiana_17031_16_2_2016_NA
EPI_ISL_308898_A_canine_Illinois_20170110_1G4_2016_NA
EPI_ISL_17605903_A_dog_Indiana_M16_09378_32_1_2016_NA
EPI_ISL_329183_A_canine_Illinois_0913751_16_1_1_2016_NA
EPI_ISL_329175_A_canine_Indiana_093165_16_1_1_2016_NA
EPI_ISL_308895_A_canine_Illinois_20170110_2G1_2016_NA
EPI_ISL_308897_A_canine_Illinois_20170110_2F1_2016_NA
EPI_ISL_308874_A_canine_South_Korea_20170110_1F1_2016_NA
EPI_ISL_309182_A_canine_Korea_AS_03_2012_NA
EPI_ISL_309190_A_canine_Korea_AS_11_2012_NA
EPI_ISL_309185_A_canine_Korea_AS_06_2012_NA
EPI_ISL_309188_A_canine_Korea_AS_11_2013_NA
A_canine_Korea_S3001_2015_H3N2__NA
EPI_ISL_309183_A_canine_Korea_AS_04_2012_NA
EPI_ISL_309189_A_canine_Korea_AS_14_2013_NA
EPI_ISL_309184_A_canine_Korea_AS_05_2012_NA
EPI_ISL_309187_A_canine_Korea_AS_09_2012_NA
EPI_ISL_309186_A_canine_Korea_AS_08_2012_NA
EPI_ISL_170565_A_canine_Korea_S1_2012_NA
EPI_ISL_217470_A_canine_Korea_DG1_2014_NA
EPI_ISL_218443_A_canine_Korea_B0_1_2013_NA
EPI_ISL_379511_A_canine_Beijing_20131203_341_2013_NA
EPI_ISL_379512_A_canine_Beijing_20131211_279_2013_NA
EPI_ISL_379546_A_canine_Beijing_20140110_2_2014_NA
EPI_ISL_379547_A_canine_Beijing_20140103_90_2014_NA
EPI_ISL_379508_A_canine_Beijing_20131203_342_2013_NA
EPI_ISL_379510_A_canine_Beijing_20131203_342_2013_NA
EPI_ISL_328524_A_canine_California_043670_2017_NA
EPI_ISL_325522_A_canine_China_JLM1_2015_NA
EPI_ISL_285503_A_canine_China_JLM2_2015_NA
EPI_ISL_297933_A_canine_China_LG1_2015_NA
EPI_ISL_297934_A_canine_China_LG2_2015_NA
EPI_ISL_379509_A_canine_Beijing_20131204_349_2013_NA
EPI_ISL_379504_A_canine_Beijing_20212230_62_2012_NA
EPI_ISL_379507_A_canine_Beijing_20131203_34_2012_NA
EPI_ISL_151165_A_canine_Guangdong_12_2012_NA
EPI_ISL_151164_A_canine_Guangdong_23_2012_NA
EPI_ISL_170602_A_canine_Thailand_CU_DC5299_2012_NA
EPI_ISL_139948_A_canine_Guangdong_05_2011_NA
EPI_ISL_142882_A_canine_Guangdong_04_2011_NA
EPI_ISL_142436_A_canine_Liaoning_27_2012_NA
EPI_ISL_162438_A_canine_Liaoning_HE_2012_NA
EPI_ISL_162440_A_canine_Heilongjiang_L1_2013_NA
EPI_ISL_142881_A_canine_Guangdong_3_2011_NA
EPI_ISL_329147_A_canine_Guangdong_04_2012_NA
EPI_ISL_329148_A_canine_Guangdong_01_2014_NA
EPI_ISL_329149_A_canine_Guangdong_02_2014_NA
EPI_ISL_329146_A_canine_Guangdong_03_2013_NA
EPI_ISL_93814_A_canine_Jiangsu_04_2010_NA
EPI_ISL_93813_A_canine_Jiangsu_05_2010_NA
EPI_ISL_93817_A_canine_Jiangsu_01_2009_NA
EPI_ISL_93816_A_canine_Jiangsu_02_2010_NA
EPI_ISL_93815_A_canine_Jiangsu_03_2010_NA
EPI_ISL_93812_A_canine_Jiangsu_06_2010_NA
EPI_ISL_129393_A_canine_Zhejiang_1_2010_NA
EPI_ISL_309181_A_canine_Korea_AS_01_2009_NA
EPI_ISL_139997_A_canine_Korea_CY009_2010_NA
EPI_ISL_279526_A_canine_Korea_CY053_2014_NA
EPI_ISL_129987_A_canine_Korea_KRIB801_2011_NA
EPI_ISL_379542_A_canine_Beijing_20150124_300_2015_NA
EPI_ISL_379543_A_canine_Beijing_20150122_284_2015_NA
EPI_ISL_379544_A_canine_Beijing_20150120_265_2015_NA
EPI_ISL_379545_A_canine_Beijing_20150118_256_2015_NA
EPI_ISL_80505_A_canine_Guangdong_1_2006_NA
EPI_ISL_139938_A_canine_Guangdong_06_2011_NA
EPI_ISL_80508_A_canine_Guangdong_1_2007_NA
EPI_ISL_80506_A_canine_Guangdong_2_2006_NA
EPI_ISL_80507_A_canine_Guangdong_2_2007_NA
EPI_ISL_143454_A_canine_Korea_MV1_2012_NA
EPI_ISL_167144_A_canine_KC123978_2012_NA
EPI_ISL_167143_A_canine_Korea_VC378_2012_NA
EPI_ISL_127502_A_canine_Korea_01_2007_NA
0.004

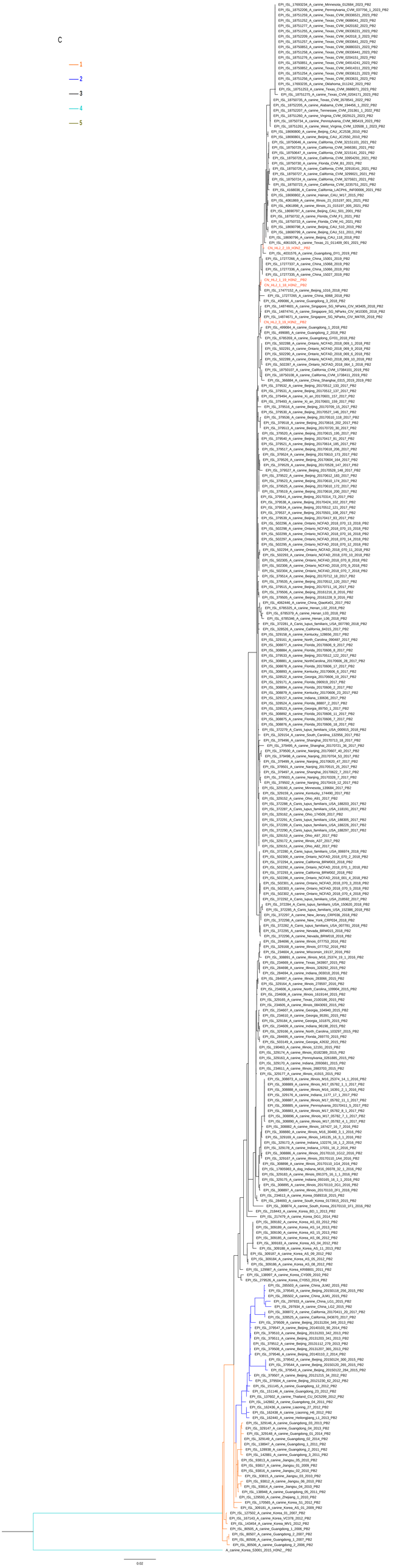
C
1
2
3
4
5
EPI_ISL_17693234_A_canine_Minnesota_012684_2023_PB2
EPI_ISL_18752206_A_canine_Pennsylvania_CVM_037756_1_2023_PB2
EPI_ISL_18751256_A_canine_Texas_CVM_09336521_2023_PB2
EPI_ISL_18751252_A_canine_Texas_CVM_0688041_2023_PB2
EPI_ISL_18751277_A_canine_Texas_CVM_0420182_2023_PB2
EPI_ISL_18751255_A_canine_Texas_CVM_09336223_2023_PB2
EPI_ISL_18752209_A_canine_Texas_CVM_042018_3_2023_PB2
EPI_ISL_18751257_A_canine_Texas_CVM_0933641_2023_PB2
EPI_ISL_18750853_A_canine_Texas_CVM_06880321_2023_PB2
EPI_ISL_18751258_A_canine_Texas_CVM_09336441_2023_PB2
EPI_ISL_18751276_A_canine_Texas_CVM_0204151_2023_PB2
EPI_ISL_18750851_A_canine_Texas_CVM_04914241_2023_PB2
EPI_ISL_18750852_A_canine_Texas_CVM_04914151_2023_PB2
EPI_ISL_18751254_A_canine_Texas_CVM_09336121_2023_PB2
EPI_ISL_18751256_A_canine_Texas_CVM_0933631_2023_PB2
EPI_ISL_17693235_A_canine_Oklahoma_011242_2023_PB2
EPI_ISL_18751253_A_canine_Texas_CVM_0688071_2023_PB2
EPI_ISL_18751275_A_canine_Texas_CVM_0204171_2023_PB2
EPI_ISL_18750735_A_canine_Texas_CVM_3078041_2022_PB2
EPI_ISL_18752205_A_canine_Alabama_CVM_194456_1_2022_PB2
EPI_ISL_18752207_A_canine_Tennessee_CVM_233381_1_2022_PB2
EPI_ISL_18751260_A_canine_Virginia_CVM_0029123_2023_PB2
EPI_ISL_18750734_A_canine_Pennsylvania_CVM_985419_2023_PB2
EPI_ISL_18751261_A_canine_West_Virginia_CVM_1_03508_1_2023_PB2
EPI_ISL_18690800_A_canine_Beijing_CAU_JC2538_2010_PB2
EPI_ISL_18690801_A_canine_Beijing_CAU_JC2550_2010_PB2
EPI_ISL_18750646_A_canine_California_CVM_31151101_2021_PB2
EPI_ISL_18750729_A_canine_California_CVM_3466381_2021_PB2
EPI_ISL_18750647_A_canine_California_CVM_32115141_2021_PB2
EPI_ISL_18750728_A_canine_California_CVM_33954281_2021_PB2
EPI_ISL_18750730_A_canine_Florida_CVM_B1_2021_PB2
EPI_ISL_18750726_A_canine_California_CVM_32918141_2021_PB2
EPI_ISL_18750727_A_canine_California_CVM_32990221_2021_PB2
EPI_ISL_18750724_A_canine_California_CVM_3275921_2021_PB2
EPI_ISL_18750723_A_canine_California_CVM_3235753_2021_PB2
EPI_ISL_4168036_A_Canine_California_LACPHL_INF00006_2021_PB2
EPI_ISL_18690802_A_canine_Hainan_CAU_W37_2015_PB2
EPI_ISL_4061869_A_canine_Illinois_21_015197_001_2021_PB2
EPI_ISL_4061898_A_canine_Illinois_21_015197_005_2021_PB2
EPI_ISL_18690797_A_canine_Beijing_CAU_S01_2001_PB2
EPI_ISL_18750732_A_canine_Florida_CVM_F1_2021_PB2
EPI_ISL_18750733_A_canine_Florida_CVM_H1_2021_PB2
EPI_ISL_18690798_A_canine_Beijing_CAU_S10_2010_PB2
EPI_ISL_18690799_A_canine_Beijing_CAU_S11_2011_PB2
EPI_ISL_18690796_A_canine_Beijing_CAU_118_2018_PB2
EPI_ISL_4061925_A_canine_Texas_21_011409_001_2021_PB2
CN_HL3_2_15_H3N2__PB2
EPI_ISL_4031576_A_canine_Guangdong_DY1_2019_PB2
EPI_ISL_17272266_A_canine_China_15001_2019_PB2
EPI_ISL_17277337_A_canine_China_15068_2019_PB2
EPI_ISL_17277336_A_canine_China_15066_2019_PB2
EPI_ISL_17277335_A_canine_China_15027_2019_PB2
CN_HL3_1_19_H3N2__PB2
CN_HL3_1_18_H3N2__PB2
EPI_ISL_17477152_A_canine_Beijing_1016_2018_PB2
EPI_ISL_17277265_A_canine_China_6068_2018_PB2
EPI_ISL_499086_A_canine_Guangdong_3_2018_PB2
EPI_ISL_14874601_A_canine_Singapore_SG_NParks_CIV_M3405_2018_PB2
EPI_ISL_14874741_A_canine_Singapore_SG_NParks_CIV_M10305_2018_PB2
EPI_ISL_14874671_A_canine_Singapore_SG_NParks_CIV_M4705_2018_PB2
CN_HL3_3_19_H3N2__PB2
EPI_ISL_499084_A_canine_Guangdong_1_2018_PB2
EPI_ISL_499085_A_canine_Guangdong_2_2018_PB2
EPI_ISL_6785359_A_canine_Guangdong_GY01_2018_PB2
EPI_ISL_502288_A_canine_Ontario_NCFAD_2018_069_1_2018_PB2
EPI_ISL_502291_A_canine_Ontario_NCFAD_2018_069_3_2018_PB2
EPI_ISL_502290_A_canine_Ontario_NCFAD_2018_069_6_2018_PB2
EPI_ISL_502289_A_canine_Ontario_NCFAD_2018_069_10_2018_PB2
EPI_ISL_502287_A_canine_Ontario_NCFAD_2018_064_1_2018_PB2
EPI_ISL_18750107_A_canine_California_CVM_173841O1_2019_PB2
EPI_ISL_18750108_A_canine_California_CVM_173841I1_2019_PB2
EPI_ISL_366884_A_canine_China_Shanghai_0315_2019_2019_PB2
EPI_ISL_379532_A_canine_Beijing_20170512_133_2017_PB2
EPI_ISL_379531_A_canine_Beijing_20170512_137_2017_PB2
EPI_ISL_379494_A_canine_Xi_an_20170601_157_2017_PB2
EPI_ISL_379493_A_canine_Xi_an_20170601_159_2017_PB2
EPI_ISL_379514_A_canine_Beijing_20170709_15_2017_PB2
EPI_ISL_379530_A_canine_Beijing_20170527_146_2017_PB2
EPI_ISL_379536_A_canine_Beijing_20170510_118_2017_PB2
EPI_ISL_379518_A_canine_Beijing_20170616_202_2017_PB2
EPI_ISL_379513_A_canine_Beijing_20170720_30_2017_PB2
EPI_ISL_379520_A_canine_Beijing_20170615_195_2017_PB2
EPI_ISL_379540_A_canine_Beijing_20170417_81_2017_PB2
EPI_ISL_379521_A_canine_Beijing_20170614_185_2017_PB2
EPI_ISL_379517_A_canine_Beijing_20170618_206_2017_PB2
EPI_ISL_379524_A_canine_Beijing_20170610_173_2017_PB2
EPI_ISL_379526_A_canine_Beijing_20170604_164_2017_PB2
EPI_ISL_379529_A_canine_Beijing_20170528_147_2017_PB2
EPI_ISL_379527_A_canine_Beijing_20170528_148_2017_PB2
EPI_ISL_379522_A_canine_Beijing_20170612_183_2017_PB2
EPI_ISL_379523_A_canine_Beijing_20170610_174_2017_PB2
EPI_ISL_379525_A_canine_Beijing_20170610_172_2017_PB2
EPI_ISL_379519_A_canine_Beijing_20170616_200_2017_PB2
EPI_ISL_379541_A_canine_Beijing_20170314_73_2017_PB2
EPI_ISL_379538_A_canine_Beijing_20170424_102_2017_PB2
EPI_ISL_379534_A_canine_Beijing_20170512_121_2017_PB2
EPI_ISL_379537_A_canine_Beijing_20170501_108_2017_PB2
EPI_ISL_379539_A_canine_Beijing_20170417_81_2017_PB2
EPI_ISL_502296_A_canine_Ontario_NCFAD_2018_070_13_2018_PB2
EPI_ISL_502298_A_canine_Ontario_NCFAD_2018_070_15_2018_PB2
EPI_ISL_502299_A_canine_Ontario_NCFAD_2018_070_16_2018_PB2
EPI_ISL_502297_A_canine_Ontario_NCFAD_2018_070_14_2018_PB2
EPI_ISL_502295_A_canine_Ontario_NCFAD_2018_070_12_2018_PB2
EPI_ISL_502294_A_canine_Ontario_NCFAD_2018_070_11_2018_PB2
EPI_ISL_502293_A_canine_Ontario_NCFAD_2018_070_10_2018_PB2
EPI_ISL_502305_A_canine_Ontario_NCFAD_2018_070_8_2018_PB2
EPI_ISL_502306_A_canine_Ontario_NCFAD_2018_070_9_2018_PB2
EPI_ISL_502304_A_canine_Ontario_NCFAD_2018_070_7_2018_PB2
EPI_ISL_379514_A_canine_Beijing_20170712_18_2017_PB2
EPI_ISL_379535_A_canine_Beijing_20170512_120_2017_PB2
EPI_ISL_379515_A_canine_Beijing_20170711_16_2017_PB2
EPI_ISL_379506_A_canine_Beijing_20161216_8_2016_PB2
EPI_ISL_379505_A_canine_Beijing_20161228_9_2016_PB2
EPI_ISL_4062446_A_canine_China_QiaoKe01_2017_PB2
EPI_ISL_6785325_A_canine_Henan_L02_2018_PB2
EPI_ISL_6785379_A_canine_Henan_L03_2018_PB2
EPI_ISL_6785346_A_canine_Henan_L06_2018_PB2
EPI_ISL_372281_A_Canis_lupus_familiaris_USA_007780_2018_PB2
EPI_ISL_328526_A_canine_California_84315_2017_PB2
EPI_ISL_329158_A_canine_Kentucky_128656_2017_PB2
EPI_ISL_329161_A_canine_North_Carolina_090487_2017_PB2
EPI_ISL_308877_A_canine_Florida_20170606_3_2017_PB2
EPI_ISL_308884_A_canine_Florida_20170606_8_2017_PB2
EPI_ISL_379533_A_canine_Beijing_20170512_122_2017_PB2
EPI_ISL_308881_A_canine_NorthCarolina_20170606_28_2017_PB2
EPI_ISL_308878_A_canine_Florida_20170606_17_2017_PB2
EPI_ISL_308893_A_canine_Kentucky_20170606_6_2017_PB2
EPI_ISL_328522_A_canine_Georgia_20170606_19_2017_PB2
EPI_ISL_329171_A_canine_Florida_090919_2017_PB2
EPI_ISL_308894_A_canine_Florida_20170606_2_2017_PB2
EPI_ISL_308879_A_canine_Kentucky_20170606_23_2017_PB2
EPI_ISL_329157_A_canine_Indiana_130636_2017_PB2
EPI_ISL_328524_A_canine_Florida_88807_2_2017_PB2
EPI_ISL_328523_A_canine_Georgia_89750_1_2017_PB2
EPI_ISL_308892_A_canine_Florida_20170606_11_2017_PB2
EPI_ISL_308875_A_canine_Florida_20170606_7_2017_PB2
EPI_ISL_308876_A_canine_Florida_20170606_18_2017_PB2
EPI_ISL_372279_A_Canis_lupus_familiaris_USA_000915_2018_PB2
EPI_ISL_329154_A_canine_South_Carolina_132956_2017_PB2
EPI_ISL_379496_A_canine_Shanghai_20170713_18_2017_PB2
EPI_ISL_379495_A_canine_Shanghai_20170721_36_2017_PB2
EPI_ISL_379500_A_canine_Nanjing_20170607_40_2017_PB2
EPI_ISL_379498_A_canine_Nanjing_20170704_53_2017_PB2
EPI_ISL_379499_A_canine_Nanjing_20170620_47_2017_PB2
EPI_ISL_379501_A_canine_Nanjing_20170515_25_2017_PB2
EPI_ISL_379497_A_canine_Shanghai_20170622_7_2017_PB2
EPI_ISL_379503_A_canine_Nanjing_20170228_7_2017_PB2
EPI_ISL_379502_A_canine_Nanjing_20170419_12_2017_PB2
EPI_ISL_329160_A_canine_Minnesota_139684_2017_PB2
EPI_ISL_329159_A_canine_Kentucky_174490_2017_PB2
EPI_ISL_329152_A_canine_Ohio_A81_2017_PB2
EPI_ISL_372288_A_Canis_lupus_familiaris_USA_188203_2017_PB2
EPI_ISL_372287_A_Canis_lupus_familiaris_USA_118191_2017_PB2
EPI_ISL_329162_A_canine_Ohio_174509_2017_PB2
EPI_ISL_372291_A_Canis_lupus_familiaris_USA_188305_2017_PB2
EPI_ISL_372289_A_Canis_lupus_familiaris_USA_188226_2017_PB2
EPI_ISL_372290_A_Canis_lupus_familiaris_USA_188297_2017_PB2
EPI_ISL_329153_A_canine_Ohio_A97_2017_PB2
EPI_ISL_329172_A_canine_Illinois_A37_2017_PB2
EPI_ISL_329151_A_canine_Ohio_A82_2017_PB2
EPI_ISL_372280_A_Canis_lupus_familiaris_USA_006974_2018_PB2
EPI_ISL_502300_A_canine_Ontario_NCFAD_2018_070_2_2018_PB2
EPI_ISL_372294_A_canine_California_BRW003_2018_PB2
EPI_ISL_502292_A_canine_Ontario_NCFAD_2018_070_1_2018_PB2
EPI_ISL_372293_A_canine_California_BRW002_2018_PB2
EPI_ISL_502286_A_canine_Ontario_NCFAD_2018_001_4_2018_PB2
EPI_ISL_502301_A_canine_Ontario_NCFAD_2018_070_3_2018_PB2
EPI_ISL_502303_A_canine_Ontario_NCFAD_2018_070_5_2018_PB2
EPI_ISL_502302_A_canine_Ontario_NCFAD_2018_070_4_2018_PB2
EPI_ISL_372292_A_Canis_lupus_familiaris_USA_218592_2017_PB2
EPI_ISL_372284_A_Canis_lupus_familiaris_USA_150620_2018_PB2
EPI_ISL_372285_A_Canis_lupus_familiaris_USA_152386_2018_PB2
EPI_ISL_372297_A_canine_New_Jersey_CRP036_2018_PB2
EPI_ISL_372298_A_canine_New_York_CRP034_2018_PB2
EPI_ISL_372282_A_Canis_lupus_familiaris_USA_007781_2018_PB2
EPI_ISL_372295_A_canine_Nevada_BRW015_2018_PB2
EPI_ISL_372296_A_canine_Nevada_BRW018_2018_PB2
EPI_ISL_284696_A_canine_Illinois_077753_2016_PB2
EPI_ISL_329168_A_canine_Illinois_077752_2016_PB2
EPI_ISL_234604_A_canine_Wisconsin_19137_2016_PB2
EPI_ISL_308891_A_canine_Illinois_M16_25374_19_1_2016_PB2
EPI_ISL_284669_A_canine_Texas_343897_2015_PB2
EPI_ISL_284698_A_canine_Illinois_328292_2015_PB2
EPI_ISL_284694_A_canine_Indiana_003018_2016_PB2
EPI_ISL_284697_A_canine_Illinois_283066_2015_PB2
EPI_ISL_329164_A_canine_Illinois_278597_2016_PB2
EPI_ISL_234606_A_canine_North_Carolina_109904_2015_PB2
EPI_ISL_234608_A_canine_Illinois_1619144_2015_PB2
EPI_ISL_329165_A_canine_Texas_2100186_2015_PB2
EPI_ISL_234605_A_canine_Illinois_08430I3_2015_PB2
EPI_ISL_234607_A_canine_Georgia_104940_2015_PB2
EPI_ISL_234610_A_canine_Georgia_95391_2015_PB2
EPI_ISL_329166_A_canine_Georgia_101879_2015_PB2
EPI_ISL_234609_A_canine_Indiana_96198_2015_PB2
EPI_ISL_329166_A_canine_North_Carolina_103297_2015_PB2
EPI_ISL_284695_A_canine_Florida_269770_2015_PB2
EPI_ISL_503149_A_canine_Georgia_42632_2015_PB2
EPI_ISL_190463_A_canine_Illinois_12191_2015_PB2
EPI_ISL_329174_A_canine_Illinois_91823B9_2015_PB2
EPI_ISL_329163_A_canine_Pennsylvania_0261885_2015_PB2
EPI_ISL_329170_A_canine_Indiana_2009381_2015_PB2
EPI_ISL_234611_A_canine_Illinois_2883703_2015_PB2
EPI_ISL_329177_A_canine_Illinois_41915_2015_PB2
EPI_ISL_308873_A_canine_Illinois_M16_25374-14_1_2016_PB2
EPI_ISL_308889_A_canine_Illinois_M17_05782_1_1_2017_PB2
EPI_ISL_308888_A_canine_Illinois_M16_16391_2_1_2016_PB2
EPI_ISL_329176_A_canine_Indiana_1177_17_1_2017_PB2
EPI_ISL_308887_A_canine_Illinois_M17_05782_11_1_2017_PB2
EPI_ISL_308885_A_canine_Pennsylvania_20170411_5_2017_PB2
EPI_ISL_308883_A_canine_Illinois_M17_05782_8_1_2017_PB2
EPI_ISL_308896_A_canine_Illinois_M17_05782_7_1_2017_PB2
EPI_ISL_308890_A_canine_Illinois_M17_05782_4_1_2017_PB2
EPI_ISL_308882_A_canine_Illinois_187427_16_7_2016_PB2
EPI_ISL_308880_A_canine_Illinois_M16_30480_3_1_2016_PB2
EPI_ISL_329169_A_canine_Illinois_145135_16_3_1_2016_PB2
EPI_ISL_329173_A_canine_Indiana_132276_16_1_2_2016_PB2
EPI_ISL_329178_A_canine_Indiana_17031_16_2_2016_PB2
EPI_ISL_308886_A_canine_Illinois_20170110_1G12_2016_PB2
EPI_ISL_329167_A_canine_Illinois_20170110_144_2016_PB2
EPI_ISL_308898_A_canine_Illinois_20170110_1G4_2016_PB2
EPI_ISL_17805983_A_dog_Indiana_M16_09378_32_1_2016_PB2
EPI_ISL_329183_A_canine_Illinois_091375_16_1_1_2016_PB2
EPI_ISL_329175_A_canine_Indiana_093165_16_1_1_2016_PB2
EPI_ISL_308895_A_canine_Illinois_20170110_2G1_2016_PB2
EPI_ISL_308897_A_canine_Illinois_20170110_2F1_2016_PB2
EPI_ISL_234613_A_canine_Korea_05893I18_2015_PB2
EPI_ISL_284693_A_canine_South_Korea_0173915_2015_PB2
EPI_ISL_308874_A_canine_South_Korea_20170110_1F1_2016_PB2
EPI_ISL_218443_A_canine_Korea_BO_1_2013_PB2
EPI_ISL_217479_A_canine_Korea_DG1_2014_PB2
EPI_ISL_305182_A_canine_Korea_AS_03_2012_PB2
EPI_ISL_305189_A_canine_Korea_AS_14_2013_PB2
EPI_ISL_305190_A_canine_Korea_AS_15_2013_PB2
EPI_ISL_305185_A_canine_Korea_AS_06_2012_PB2
EPI_ISL_305183_A_canine_Korea_AS_04_2012_PB2
EPI_ISL_305188_A_canine_Korea_AS_11_2013_PB2
EPI_ISL_305187_A_canine_Korea_AS_09_2012_PB2
EPI_ISL_305184_A_canine_Korea_AS_05_2012_PB2
EPI_ISL_305186_A_canine_Korea_AS_08_2012_PB2
EPI_ISL_129967_A_canine_Korea_KR6B801_2011_PB2
EPI_ISL_138997_A_canine_Korea_CY009_2010_PB2
EPI_ISL_279526_A_canine_Korea_CY053_2014_PB2
EPI_ISL_285503_A_canine_China_JLM2_2015_PB2
EPI_ISL_379545_A_canine_Beijing_20150118_256_2015_PB2
EPI_ISL_285502_A_canine_China_JLM1_2015_PB2
EPI_ISL_297933_A_canine_China_LG1_2015_PB2
EPI_ISL_297934_A_canine_China_LG2_2015_PB2
EPI_ISL_308872_A_canine_California_20170411_20_2017_PB2
EPI_ISL_328525_A_canine_California_043670_2017_PB2
EPI_ISL_379509_A_canine_Beijing_20131204_349_2013_PB2
EPI_ISL_379547_A_canine_Beijing_20140103_90_2014_PB2
EPI_ISL_379510_A_canine_Beijing_20131203_342_2013_PB2
EPI_ISL_379511_A_canine_Beijing_20131203_341_2013_PB2
EPI_ISL_379512_A_canine_Beijing_20131112_279_2013_PB2
EPI_ISL_379508_A_canine_Beijing_20131207_365_2013_PB2
EPI_ISL_379546_A_canine_Beijing_20140110_2_2014_PB2
EPI_ISL_379542_A_canine_Beijing_20150124_300_2015_PB2
EPI_ISL_379544_A_canine_Beijing_20150120_265_2015_PB2
EPI_ISL_379543_A_canine_Beijing_20150122_284_2015_PB2
EPI_ISL_379507_A_canine_Beijing_20121215_34_2012_PB2
EPI_ISL_379504_A_canine_Beijing_20121230_82_2012_PB2
EPI_ISL_151145_A_canine_Guangdong_12_2012_PB2
EPI_ISL_151146_A_canine_Guangdong_23_2012_PB2
EPI_ISL_137002_A_canine_Thailand_CU_DC5299_2012_PB2
EPI_ISL_142882_A_canine_Guangdong_04_2013_PB2
EPI_ISL_162436_A_canine_Liaoning_27_2012_PB2
EPI_ISL_162438_A_canine_Liaoning_H6_2012_PB2
EPI_ISL_162440_A_canine_Heilongjiang_L1_2013_PB2
EPI_ISL_329146_A_canine_Guangdong_03_2013_PB2
EPI_ISL_329147_A_canine_Guangdong_04_2013_PB2
EPI_ISL_329148_A_canine_Guangdong_01_2014_PB2
EPI_ISL_329149_A_canine_Guangdong_02_2014_PB2
EPI_ISL_138947_A_canine_Guangdong_1_2011_PB2
EPI_ISL_129938_A_canine_Guangdong_2_2011_PB2
EPI_ISL_142881_A_canine_Guangdong_3_2011_PB2
EPI_ISL_93813_A_canine_Jiangsu_05_2010_PB2
EPI_ISL_93817_A_canine_Jiangsu_01_2009_PB2
EPI_ISL_93814_A_canine_Jiangsu_02_2010_PB2
EPI_ISL_93815_A_canine_Jiangsu_03_2010_PB2
EPI_ISL_93812_A_canine_Jiangsu_06_2010_PB2
EPI_ISL_93814_A_canine_Jiangsu_04_2010_PB2
EPI_ISL_138948_A_canine_Guangdong_05_2011_PB2
EPI_ISL_129593_A_canine_Zhejiang_1_2010_PB2
EPI_ISL_170505_A_canine_Korea_S1_2012_PB2
EPI_ISL_309183_A_canine_Korea_A5_01_2009_PB2
EPI_ISL_127502_A_canine_Korea_01_2007_PB2
EPI_ISL_167143_A_canine_Korea_VC378_2012_PB2
EPI_ISL_143454_A_canine_Korea_MV1_2012_PB2
EPI_ISL_80505_A_canine_Guangdong_1_2006_PB2
EPI_ISL_80507_A_canine_Guangdong_2_2007_PB2
EPI_ISL_80508_A_canine_Guangdong_1_2007_PB2
EPI_ISL_80506_A_canine_Guangdong_2_2006_PB2
A_canine_Korea_S3001_2015_H3N2__PB2
0.02

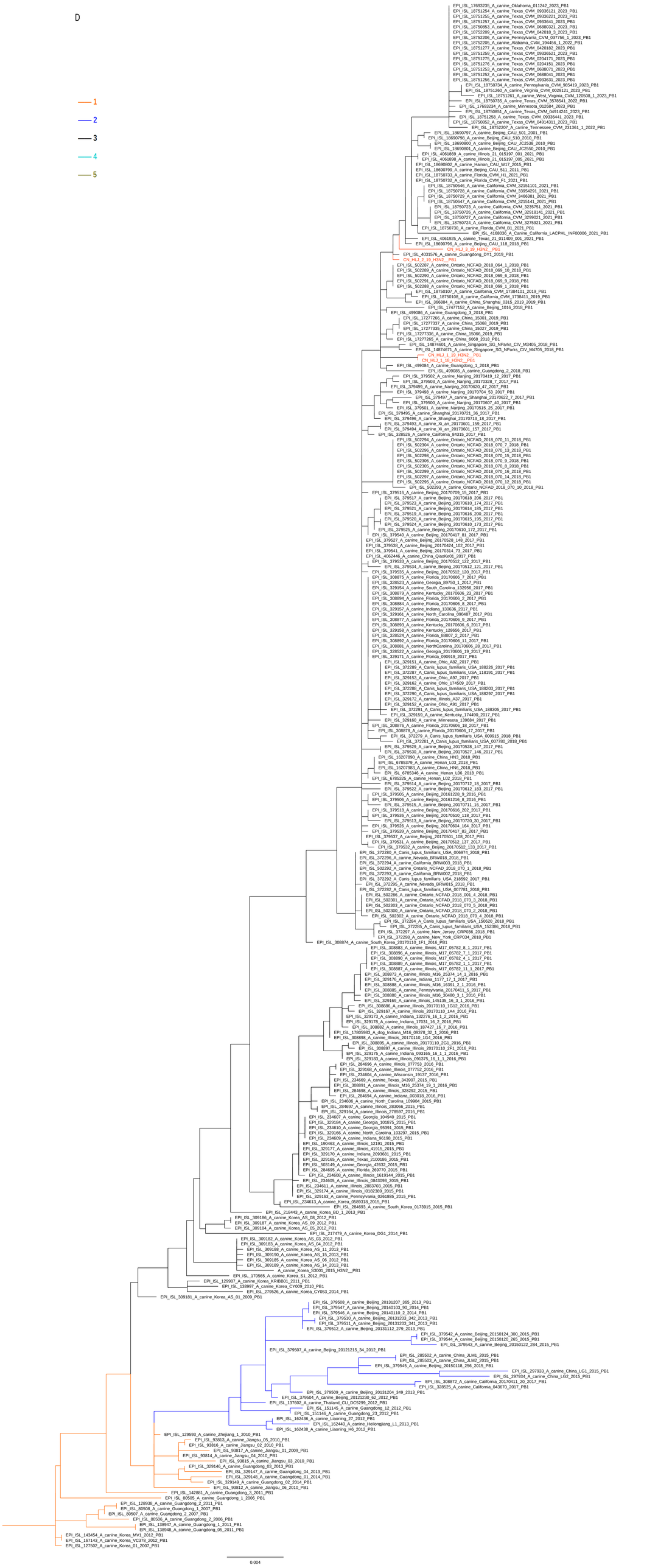
D
1
2
3
4
5
EPI_ISL_17693235_A_canine_Oklahoma_011242_2023_PB1
EPI_ISL_18751254_A_canine_Texas_CVM_09336121_2023_PB1
EPI_ISL_18751255_A_canine_Texas_CVM_09336221_2023_PB1
EPI_ISL_18751257_A_canine_Texas_CVM_0933641_2023_PB1
EPI_ISL_18750853_A_canine_Texas_CVM_06880321_2023_PB1
EPI_ISL_18752209_A_canine_Texas_CVM_042018_3_2023_PB1
EPI_ISL_18752206_A_canine_Pennsylvania_CVM_037756_1_2023_PB1
EPI_ISL_18752205_A_canine_Alabama_CVM_194456_1_2022_PB1
EPI_ISL_18751277_A_canine_Texas_CVM_0420182_2023_PB1
EPI_ISL_18751259_A_canine_Texas_CVM_09336521_2023_PB1
EPI_ISL_18751275_A_canine_Texas_CVM_0204171_2023_PB1
EPI_ISL_18751276_A_canine_Texas_CVM_0204151_2023_PB1
EPI_ISL_18751253_A_canine_Texas_CVM_0688071_2023_PB1
EPI_ISL_18751252_A_canine_Texas_CVM_0688041_2023_PB1
EPI_ISL_18751256_A_canine_Texas_CVM_0933631_2023_PB1
EPI_ISL_18750734_A_canine_Pennsylvania_CVM_985419_2023_PB1
EPI_ISL_18751260_A_canine_Virginia_CVM_00291221_2023_PB1
EPI_ISL_18751261_A_canine_West_Virginia_CVM_120508_1_2023_PB1
EPI_ISL_18750735_A_canine_Texas_CVM_3578541_2022_PB1
EPI_ISL_17693234_A_canine_Minnesota_012684_2023_PB1
EPI_ISL_18750851_A_canine_Texas_CVM_04914241_2023_PB1
EPI_ISL_18751258_A_canine_Texas_CVM_09336441_2023_PB1
EPI_ISL_18750852_A_canine_Texas_CVM_04914311_2023_PB1
EPI_ISL_18752207_A_canine_Tennessee_CVM_231361_1_2022_PB1
EPI_ISL_18690797_A_canine_Beijing_CAU_501_2001_PB1
EPI_ISL_18690798_A_canine_Beijing_CAU_510_2010_PB1
EPI_ISL_18690800_A_canine_Beijing_CAU_JC2538_2010_PB1
EPI_ISL_18690801_A_canine_Beijing_CAU_JC2550_2010_PB1
EPI_ISL_4061869_A_canine_Illinois_21_015197_001_2021_PB1
EPI_ISL_4061898_A_canine_Illinois_21_015197_005_2021_PB1
EPI_ISL_18690802_A_canine_Hainan_CAU_W17_2015_PB1
EPI_ISL_18690799_A_canine_Beijing_CAU_511_2011_PB1
EPI_ISL_18750733_A_canine_Florida_CVM_H1_2021_PB1
EPI_ISL_18750732_A_canine_Florida_CVM_F1_2021_PB1
EPI_ISL_18750646_A_canine_California_CVM_3215101_2021_PB1
EPI_ISL_18750728_A_canine_California_CVM_33954291_2021_PB1
EPI_ISL_18750729_A_canine_California_CVM_3466381_2021_PB1
EPI_ISL_18750647_A_canine_California_CVM_3215141_2021_PB1
EPI_ISL_18750723_A_canine_California_CVM_3235751_2021_PB1
EPI_ISL_18750726_A_canine_California_CVM_3291841_2021_PB1
EPI_ISL_18750727_A_canine_California_CVM_3299021_2021_PB1
EPI_ISL_18750724_A_canine_California_CVM_3275921_2021_PB1
EPI_ISL_18750730_A_canine_Florida_CVM_B1_2021_PB1
EPI_ISL_4168036_A_Canine_California_LACPHL_INF00006_2021_PB1
EPI_ISL_4061925_A_canine_Texas_21_011409_001_2021_PB1
EPI_ISL_18690796_A_canine_Beijing_CAU_118_2018_PB1
CN_HLJ_3_19_H3N2__PB1
EPI_ISL_4031576_A_canine_Guangdong_DY1_2019_PB1
CN_HLJ_2_19_H3N2__PB1
EPI_ISL_502287_A_canine_Ontario_NCFAD_2018_064_1_2018_PB1
EPI_ISL_502289_A_canine_Ontario_NCFAD_2018_069_10_2018_PB1
EPI_ISL_502290_A_canine_Ontario_NCFAD_2018_069_6_2018_PB1
EPI_ISL_502291_A_canine_Ontario_NCFAD_2018_069_9_2018_PB1
EPI_ISL_502288_A_canine_Ontario_NCFAD_2018_069_1_2018_PB1
EPI_ISL_18750107_A_canine_California_CVM_17384101_2019_PB1
EPI_ISL_18750108_A_canine_California_CVM_17384411_2019_PB1
EPI_ISL_366884_A_canine_China_Shanghai_0315_2019_2019_PB1
EPI_ISL_17477152_A_canine_Beijing_1016_2018_PB1
EPI_ISL_499086_A_canine_Guangdong_3_2018_PB1
EPI_ISL_17277266_A_canine_China_15001_2019_PB1
EPI_ISL_17277337_A_canine_China_15068_2019_PB1
EPI_ISL_17277335_A_canine_China_15027_2019_PB1
EPI_ISL_17277336_A_canine_China_15066_2019_PB1
EPI_ISL_17277265_A_canine_China_6068_2018_PB1
EPI_ISL_14874601_A_canine_Singapore_SG_NParks_CIV_M3405_2018_PB1
EPI_ISL_14874671_A_canine_Singapore_SG_NParks_CIV_M4705_2018_PB1
CN_HLJ_1_19_H3N2__PB1
CN_HLJ_1_18_H3N2__PB1
EPI_ISL_499084_A_canine_Guangdong_1_2018_PB1
EPI_ISL_499085_A_canine_Guangdong_2_2018_PB1
EPI_ISL_379502_A_canine_Nanjing_20170419_12_2017_PB1
EPI_ISL_379503_A_canine_Nanjing_20170328_7_2017_PB1
EPI_ISL_379499_A_canine_Nanjing_20170620_47_2017_PB1
EPI_ISL_379498_A_canine_Nanjing_20170704_53_2017_PB1
EPI_ISL_379497_A_canine_Shanghai_20170622_7_2017_PB1
EPI_ISL_379500_A_canine_Nanjing_20170607_40_2017_PB1
EPI_ISL_379501_A_canine_Nanjing_20170515_25_2017_PB1
EPI_ISL_379495_A_canine_Shanghai_20170721_36_2017_PB1
EPI_ISL_379496_A_canine_Shanghai_20170713_18_2017_PB1
EPI_ISL_379493_A_canine_Xi_an_20170601_159_2017_PB1
EPI_ISL_379494_A_canine_Xi_an_20170601_157_2017_PB1
EPI_ISL_328526_A_canine_California_84315_2017_PB1
EPI_ISL_502294_A_canine_Ontario_NCFAD_2018_070_11_2018_PB1
EPI_ISL_502304_A_canine_Ontario_NCFAD_2018_070_7_2018_PB1
EPI_ISL_502296_A_canine_Ontario_NCFAD_2018_070_13_2018_PB1
EPI_ISL_502298_A_canine_Ontario_NCFAD_2018_070_15_2018_PB1
EPI_ISL_502306_A_canine_Ontario_NCFAD_2018_070_9_2018_PB1
EPI_ISL_502305_A_canine_Ontario_NCFAD_2018_070_8_2018_PB1
EPI_ISL_502299_A_canine_Ontario_NCFAD_2018_070_16_2018_PB1
EPI_ISL_502297_A_canine_Ontario_NCFAD_2018_070_14_2018_PB1
EPI_ISL_502295_A_canine_Ontario_NCFAD_2018_070_12_2018_PB1
EPI_ISL_502293_A_canine_Ontario_NCFAD_2018_070_10_2018_PB1
EPI_ISL_379516_A_canine_Beijing_20170709_15_2017_PB1
EPI_ISL_379517_A_canine_Beijing_20170618_206_2017_PB1
EPI_ISL_379523_A_canine_Beijing_20170610_174_2017_PB1
EPI_ISL_379521_A_canine_Beijing_20170614_185_2017_PB1
EPI_ISL_379519_A_canine_Beijing_20170616_200_2017_PB1
EPI_ISL_379520_A_canine_Beijing_20170615_195_2017_PB1
EPI_ISL_379524_A_canine_Beijing_20170610_173_2017_PB1
EPI_ISL_379525_A_canine_Beijing_20170610_172_2017_PB1
EPI_ISL_379540_A_canine_Beijing_20170417_81_2017_PB1
EPI_ISL_379527_A_canine_Beijing_20170528_148_2017_PB1
EPI_ISL_379538_A_canine_Beijing_20170424_102_2017_PB1
EPI_ISL_379541_A_canine_Beijing_20170314_73_2017_PB1
EPI_ISL_4062446_A_canine_China_QiaoKe01_2017_PB1
EPI_ISL_379533_A_canine_Beijing_20170512_122_2017_PB1
EPI_ISL_379534_A_canine_Beijing_20170512_121_2017_PB1
EPI_ISL_379535_A_canine_Beijing_20170512_120_2017_PB1
EPI_ISL_308875_A_canine_Florida_20170606_7_2017_PB1
EPI_ISL_328523_A_canine_Georgia_89750_1_2017_PB1
EPI_ISL_329154_A_canine_South_Carolina_132996_2017_PB1
EPI_ISL_308879_A_canine_Kentucky_20170606_23_2017_PB1
EPI_ISL_308894_A_canine_Florida_20170606_2_2017_PB1
EPI_ISL_308884_A_canine_Florida_20170606_8_2017_PB1
EPI_ISL_329157_A_canine_Indiana_130636_2017_PB1
EPI_ISL_329161_A_canine_North_Carolina_090487_2017_PB1
EPI_ISL_308877_A_canine_Florida_20170606_9_2017_PB1
EPI_ISL_308893_A_canine_Kentucky_20170606_6_2017_PB1
EPI_ISL_329158_A_canine_Kentucky_128656_2017_PB1
EPI_ISL_328524_A_canine_Florida_98807_2_2017_PB1
EPI_ISL_308892_A_canine_Florida_20170606_11_2017_PB1
EPI_ISL_308881_A_canine_NorthCarolina_20170606_26_2017_PB1
EPI_ISL_328522_A_canine_Georgia_20170606_19_2017_PB1
EPI_ISL_329171_A_canine_Florida_090919_2017_PB1
EPI_ISL_329151_A_canine_Ohio_A82_2017_PB1
EPI_ISL_372289_A_Canis_lupus_familiaris_USA_188226_2017_PB1
EPI_ISL_372287_A_Canis_lupus_familiaris_USA_118191_2017_PB1
EPI_ISL_329153_A_canine_Ohio_A97_2017_PB1
EPI_ISL_329162_A_canine_Ohio_174509_2017_PB1
EPI_ISL_372288_A_Canis_lupus_familiaris_USA_188203_2017_PB1
EPI_ISL_372290_A_Canis_lupus_familiaris_USA_188297_2017_PB1
EPI_ISL_329172_A_canine_Illinois_A37_2017_PB1
EPI_ISL_329152_A_canine_Ohio_A91_2017_PB1
EPI_ISL_372291_A_Canis_lupus_familiaris_USA_188305_2017_PB1
EPI_ISL_329159_A_canine_Kentucky_174490_2017_PB1
EPI_ISL_329160_A_canine_Minnesota_139684_2017_PB1
EPI_ISL_308876_A_canine_Florida_20170606_18_2017_PB1
EPI_ISL_308878_A_canine_Florida_20170606_17_2017_PB1
EPI_ISL_372279_A_Canis_lupus_familiaris_USA_000915_2018_PB1
EPI_ISL_372281_A_Canis_lupus_familiaris_USA_007780_2018_PB1
EPI_ISL_379529_A_canine_Beijing_20170528_147_2017_PB1
EPI_ISL_379530_A_canine_Beijing_20170527_146_2017_PB1
EPI_ISL_16207890_A_canine_China_HN3_2018_PB1
EPI_ISL_6785379_A_canine_Henan_L03_2018_PB1
EPI_ISL_16207983_A_canine_China_HN6_2018_PB1
EPI_ISL_6785346_A_canine_Henan_L06_2018_PB1
EPI_ISL_6785325_A_canine_Henan_L02_2018_PB1
EPI_ISL_379514_A_canine_Beijing_20170712_18_2017_PB1
EPI_ISL_379522_A_canine_Beijing_20170612_183_2017_PB1
EPI_ISL_379505_A_canine_Beijing_20161228_9_2016_PB1
EPI_ISL_379506_A_canine_Beijing_20161216_8_2016_PB1
EPI_ISL_379515_A_canine_Beijing_20170711_16_2017_PB1
EPI_ISL_379518_A_canine_Beijing_20170616_202_2017_PB1
EPI_ISL_379536_A_canine_Beijing_20170510_118_2017_PB1
EPI_ISL_379513_A_canine_Beijing_20170720_30_2017_PB1
EPI_ISL_379526_A_canine_Beijing_20170604_164_2017_PB1
EPI_ISL_379539_A_canine_Beijing_20170417_83_2017_PB1
EPI_ISL_379537_A_canine_Beijing_20170501_108_2017_PB1
EPI_ISL_379531_A_canine_Beijing_20170512_137_2017_PB1
EPI_ISL_379532_A_canine_Beijing_20170512_133_2017_PB1
EPI_ISL_372280_A_Canis_lupus_familiaris_USA_006974_2018_PB1
EPI_ISL_372296_A_canine_Nevada_BRW018_2018_PB1
EPI_ISL_372294_A_canine_California_BRW003_2018_PB1
EPI_ISL_502292_A_canine_Ontario_NCFAD_2018_070_1_2018_PB1
EPI_ISL_372293_A_canine_California_BRW002_2018_PB1
EPI_ISL_372292_A_Canis_lupus_familiaris_USA_218592_2017_PB1
EPI_ISL_372295_A_canine_Nevada_BRW015_2018_PB1
EPI_ISL_372282_A_Canis_lupus_familiaris_USA_007781_2018_PB1
EPI_ISL_502296_A_canine_Ontario_NCFAD_2018_001_4_2018_PB1
EPI_ISL_502301_A_canine_Ontario_NCFAD_2018_070_3_2018_PB1
EPI_ISL_502303_A_canine_Ontario_NCFAD_2018_070_5_2018_PB1
EPI_ISL_502300_A_canine_Ontario_NCFAD_2018_070_2_2018_PB1
EPI_ISL_502302_A_canine_Ontario_NCFAD_2018_070_4_2018_PB1
EPI_ISL_372284_A_Canis_lupus_familiaris_USA_150620_2018_PB1
EPI_ISL_372285_A_Canis_lupus_familiaris_USA_152386_2018_PB1
EPI_ISL_372297_A_canine_New_Jersey_CRP0036_2018_PB1
EPI_ISL_372298_A_canine_New_York_CRP034_2018_PB1
EPI_ISL_308874_A_canine_South_Korea_20170110_1F1_2016_PB1
EPI_ISL_308883_A_canine_Illinois_M17_05782_8_1_2017_PB1
EPI_ISL_308896_A_canine_Illinois_M17_05782_7_1_2017_PB1
EPI_ISL_308890_A_canine_Illinois_M17_05782_4_1_2017_PB1
EPI_ISL_308889_A_canine_Illinois_M17_05782_1_1_2017_PB1
EPI_ISL_308887_A_canine_Illinois_M17_05782_11_1_2017_PB1
EPI_ISL_308873_A_canine_Illinois_M16_25374_14_1_2016_PB1
EPI_ISL_329176_A_canine_Indiana_1177_17_1_2017_PB1
EPI_ISL_308888_A_canine_Illinois_M16_16391_2_1_2016_PB1
EPI_ISL_308885_A_canine_Pennsylvania_20170411_5_2017_PB1
EPI_ISL_308880_A_canine_Illinois_M16_30480_3_1_2016_PB1
EPI_ISL_329169_A_canine_Illinois_145135_16_3_1_2016_PB1
EPI_ISL_308886_A_canine_Illinois_20170110_1G12_2016_PB1
EPI_ISL_329167_A_canine_Illinois_20170110_1a4_2016_PB1
EPI_ISL_329173_A_canine_Indiana_132276_16_1_2_2016_PB1
EPI_ISL_329178_A_canine_Indiana_17031_16_2_2016_PB1
EPI_ISL_308882_A_canine_Illinois_187427_16_7_2016_PB1
EPI_ISL_17805983_A_dog_Indiana_M16_09378_32_1_2016_PB1
EPI_ISL_308898_A_canine_Illinois_20170110_1G4_2016_PB1
EPI_ISL_308895_A_canine_Illinois_20170110_2G1_2016_PB1
EPI_ISL_308897_A_canine_Illinois_20170110_2F1_2016_PB1
EPI_ISL_329175_A_canine_Indiana_093165_16_1_1_2016_PB1
EPI_ISL_329183_A_canine_Illinois_091375_16_1_1_2016_PB1
EPI_ISL_284696_A_canine_Illinois_077753_2016_PB1
EPI_ISL_329168_A_canine_Illinois_077752_2016_PB1
EPI_ISL_234604_A_canine_Wisconsin_19137_2016_PB1
EPI_ISL_234669_A_canine_Texas_343907_2015_PB1
EPI_ISL_308891_A_canine_Illinois_M16_25374_19_1_2016_PB1
EPI_ISL_284698_A_canine_Illinois_328292_2015_PB1
EPI_ISL_284694_A_canine_Indiana_003018_2016_PB1
EPI_ISL_234606_A_canine_North_Carolina_109904_2015_PB1
EPI_ISL_284697_A_canine_Illinois_283066_2015_PB1
EPI_ISL_329164_A_canine_Illinois_278597_2016_PB1
EPI_ISL_234607_A_canine_Georgia_104940_2015_PB1
EPI_ISL_329184_A_canine_Georgia_101875_2015_PB1
EPI_ISL_234610_A_canine_Georgia_95391_2015_PB1
EPI_ISL_329166_A_canine_North_Carolina_103297_2015_PB1
EPI_ISL_234609_A_canine_Indiana_96198_2015_PB1
EPI_ISL_190463_A_canine_Illinois_12191_2015_PB1
EPI_ISL_329177_A_canine_Illinois_41915_2015_PB1
EPI_ISL_329170_A_canine_Indiana_2093681_2015_PB1
EPI_ISL_329165_A_canine_Texas_2100186_2015_PB1
EPI_ISL_503149_A_canine_Georgia_42632_2015_PB1
EPI_ISL_284695_A_canine_Florida_269770_2015_PB1
EPI_ISL_234608_A_canine_Illinois_1619144_2015_PB1
EPI_ISL_234605_A_canine_Illinois_0843093_2015_PB1
EPI_ISL_234611_A_canine_Illinois_2883703_2015_PB1
EPI_ISL_329174_A_canine_Illinois_10182389_2015_PB1
EPI_ISL_329163_A_canine_Pennsylvania_0261885_2015_PB1
EPI_ISL_234613_A_canine_Korea_0589318_2015_PB1
EPI_ISL_284693_A_canine_South_Korea_0173915_2015_PB1
EPI_ISL_218443_A_canine_Korea_BD_1_2013_PB1
EPI_ISL_309186_A_canine_Korea_AS_08_2012_PB1
EPI_ISL_309187_A_canine_Korea_AS_09_2012_PB1
EPI_ISL_309184_A_canine_Korea_AS_05_2012_PB1
EPI_ISL_217479_A_canine_Korea_DG1_2014_PB1
EPI_ISL_309182_A_canine_Korea_AS_03_2012_PB1
EPI_ISL_309183_A_canine_Korea_AS_04_2012_PB1
EPI_ISL_309188_A_canine_Korea_AS_11_2013_PB1
EPI_ISL_309190_A_canine_Korea_AS_15_2013_PB1
EPI_ISL_309185_A_canine_Korea_AS_06_2012_PB1
EPI_ISL_309189_A_canine_Korea_AS_14_2013_PB1
A_canine_Korea_S3001_2015_H3N2__PB1
EPI_ISL_170565_A_canine_Korea_S1_2012_PB1
EPI_ISL_129987_A_canine_Korea_KRIBB01_2011_PB1
EPI_ISL_138997_A_canine_Korea_CY009_2010_PB1
EPI_ISL_379526_A_canine_Korea_CY053_2014_PB1
EPI_ISL_309181_A_canine_Korea_AS_01_2009_PB1
EPI_ISL_379508_A_canine_Beijing_20131207_365_2013_PB1
EPI_ISL_379547_A_canine_Beijing_20140103_90_2014_PB1
EPI_ISL_379546_A_canine_Beijing_20140110_2_2014_PB1
EPI_ISL_379510_A_canine_Beijing_20131203_342_2013_PB1
EPI_ISL_379511_A_canine_Beijing_20131203_341_2013_PB1
EPI_ISL_379512_A_canine_Beijing_20131112_279_2013_PB1
EPI_ISL_379542_A_canine_Beijing_20150124_300_2015_PB1
EPI_ISL_379544_A_canine_Beijing_20150120_265_2015_PB1
EPI_ISL_379543_A_canine_Beijing_20150122_284_2015_PB1
EPI_ISL_379507_A_canine_Beijing_20121215_34_2012_PB1
EPI_ISL_285502_A_canine_China_JLM1_2015_PB1
EPI_ISL_285503_A_canine_China_JLM2_2015_PB1
EPI_ISL_379545_A_canine_Beijing_20150118_256_2015_PB1
EPI_ISL_297933_A_canine_China_LG1_2015_PB1
EPI_ISL_297934_A_canine_China_LG2_2015_PB1
EPI_ISL_308872_A_canine_California_20170411_20_2017_PB1
EPI_ISL_328525_A_canine_California_043670_2017_PB1
EPI_ISL_379509_A_canine_Beijing_20131204_349_2013_PB1
EPI_ISL_379504_A_canine_Beijing_20121230_62_2012_PB1
EPI_ISL_137602_A_canine_Thailand_CU_DC5299_2012_PB1
EPI_ISL_151145_A_canine_Guangdong_12_2012_PB1
EPI_ISL_151146_A_canine_Guangdong_23_2012_PB1
EPI_ISL_162436_A_canine_Liaoning_27_2012_PB1
EPI_ISL_162440_A_canine_Heilongjiang_L1_2013_PB1
EPI_ISL_162438_A_canine_Liaoning_H6_2012_PB1
EPI_ISL_129593_A_canine_Zhejiang_1_2010_PB1
EPI_ISL_93813_A_canine_Jiangsu_05_2010_PB1
EPI_ISL_93816_A_canine_Jiangsu_02_2010_PB1
EPI_ISL_93817_A_canine_Jiangsu_01_2009_PB1
EPI_ISL_93814_A_canine_Jiangsu_04_2010_PB1
EPI_ISL_93815_A_canine_Jiangsu_03_2010_PB1
EPI_ISL_329146_A_canine_Guangdong_03_2013_PB1
EPI_ISL_329147_A_canine_Guangdong_04_2013_PB1
EPI_ISL_329148_A_canine_Guangdong_01_2014_PB1
EPI_ISL_329149_A_canine_Guangdong_02_2014_PB1
EPI_ISL_93812_A_canine_Jiangsu_06_2010_PB1
EPI_ISL_142881_A_canine_Guangdong_3_2011_PB1
EPI_ISL_80505_A_canine_Guangdong_1_2006_PB1
EPI_ISL_128938_A_canine_Guangdong_2_2011_PB1
EPI_ISL_80508_A_canine_Guangdong_1_2007_PB1
EPI_ISL_80507_A_canine_Guangdong_2_2007_PB1
EPI_ISL_80506_A_canine_Guangdong_2_2006_PB1
EPI_ISL_138947_A_canine_Guangdong_1_2011_PB1
EPI_ISL_138948_A_canine_Guangdong_05_2011_PB1
EPI_ISL_143454_A_canine_Korea_MV1_2012_PB1
EPI_ISL_167143_A_canine_Korea_VC378_2012_PB1
EPI_ISL_127502_A_canine_Korea_01_2007_PB1
0.004

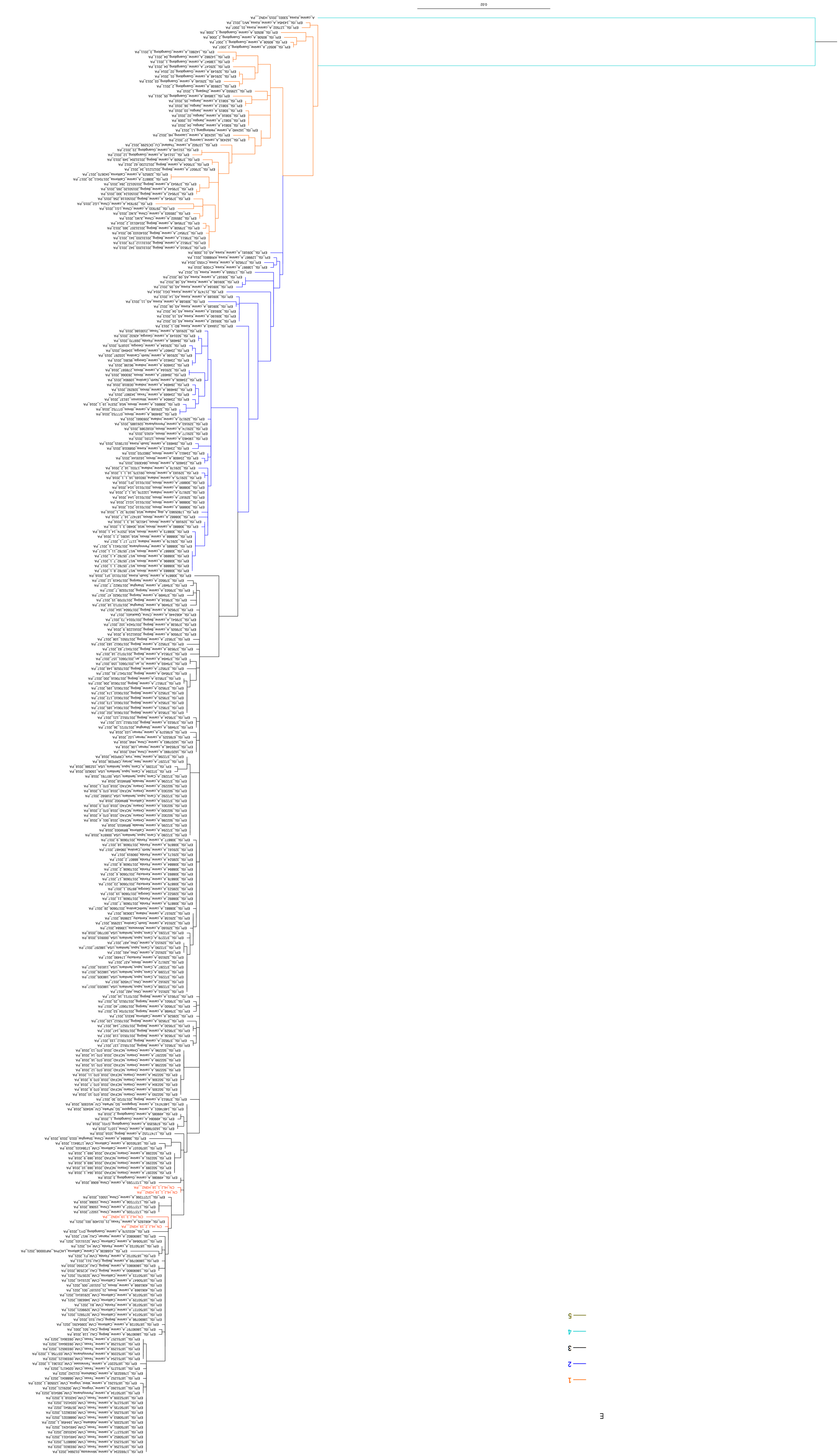
0.02
A_canine_Korea_S3001_2015_H3N2__PA
EPI_ISL_143454_A_canine_Korea_MV1_2012_PA
EPI_ISL_127502_A_canine_Korea_01_2007_PA
EPI_ISL_80505_A_canine_Guangdong_1_2006_PA
EPI_ISL_80506_A_canine_Guangdong_2_2006_PA
EPI_ISL_80508_A_canine_Guangdong_1_2007_PA
EPI_ISL_80507_A_canine_Guangdong_2_2007_PA
EPI_ISL_142681_A_canine_Guangdong_3_2011_PA
EPI_ISL_142882_A_canine_Guangdong_04_2011_PA
EPI_ISL_138947_A_canine_Guangdong_1_2011_PA
EPI_ISL_329147_A_canine_Guangdong_04_2013_PA
EPI_ISL_329149_A_canine_Guangdong_02_2014_PA
EPI_ISL_329148_A_canine_Guangdong_01_2014_PA
EPI_ISL_329146_A_canine_Guangdong_03_2013_PA
EPI_ISL_128938_A_canine_Guangdong_2_2011_PA
EPI_ISL_129593_A_canine_Zhejiang_1_2010_PA
EPI_ISL_138948_A_canine_Guangdong_05_2011_PA
EPI_ISL_183813_A_canine_Jiangsu_05_2010_PA
EPI_ISL_183812_A_canine_Jiangsu_06_2010_PA
EPI_ISL_183815_A_canine_Jiangsu_03_2010_PA
EPI_ISL_183816_A_canine_Jiangsu_02_2010_PA
EPI_ISL_183817_A_canine_Jiangsu_01_2009_PA
EPI_ISL_183814_A_canine_Jiangsu_04_2010_PA
EPI_ISL_162440_A_canine_Heilongjiang_L1_2013_PA
EPI_ISL_162438_A_canine_Liaoning_H6_2012_PA
EPI_ISL_162436_A_canine_Liaoning_27_2012_PA
EPI_ISL_137602_A_canine_Thailand_CU_DCS299_2012_PA
EPI_ISL_151145_A_canine_Guangdong_23_2012_PA
EPI_ISL_151146_A_canine_Guangdong_12_2012_PA
EPI_ISL_379509_A_canine_Beijing_20131204_349_2013_PA
EPI_ISL_379504_A_canine_Beijing_20121230_62_2012_PA
EPI_ISL_379507_A_canine_Beijing_20121215_34_2012_PA
EPI_ISL_328525_A_canine_California_043670_2017_PA
EPI_ISL_308872_A_canine_California_20170411_20_2017_PA
EPI_ISL_379543_A_canine_Beijing_20150122_284_2015_PA
EPI_ISL_379544_A_canine_Beijing_20150120_265_2015_PA
EPI_ISL_379542_A_canine_Beijing_20150124_300_2015_PA
EPI_ISL_379545_A_canine_Beijing_20150118_256_2015_PA
EPI_ISL_297934_A_canine_China_LG2_2015_PA
EPI_ISL_297933_A_canine_China_LG1_2015_PA
EPI_ISL_285503_A_canine_China_JLM2_2015_PA
EPI_ISL_285502_A_canine_China_JLM1_2015_PA
EPI_ISL_379548_A_canine_Beijing_20140110_2_2014_PA
EPI_ISL_379547_A_canine_Beijing_20140103_90_2014_PA
EPI_ISL_379508_A_canine_Beijing_20131207_365_2013_PA
EPI_ISL_379511_A_canine_Beijing_20131203_341_2013_PA
EPI_ISL_379512_A_canine_Beijing_20131112_279_2013_PA
EPI_ISL_379510_A_canine_Beijing_20131203_342_2013_PA
EPI_ISL_309181_A_canine_Korea_AS_01_2009_PA
EPI_ISL_279526_A_canine_Korea_CY053_2014_PA
EPI_ISL_129987_A_canine_Korea_KRIB801_2011_PA
EPI_ISL_138997_A_canine_Korea_CY009_2010_PA
EPI_ISL_170565_A_canine_Korea_S1_2012_PA
EPI_ISL_309187_A_canine_Korea_AS_09_2012_PA
EPI_ISL_309186_A_canine_Korea_AS_08_2012_PA
EPI_ISL_309184_A_canine_Korea_AS_05_2012_PA
EPI_ISL_217479_A_canine_Korea_DG1_2014_PA
EPI_ISL_309189_A_canine_Korea_AS_14_2013_PA
EPI_ISL_309188_A_canine_Korea_AS_11_2013_PA
EPI_ISL_309185_A_canine_Korea_AS_06_2012_PA
EPI_ISL_309183_A_canine_Korea_AS_04_2012_PA
EPI_ISL_309190_A_canine_Korea_AS_15_2013_PA
EPI_ISL_309182_A_canine_Korea_AS_03_2012_PA
EPI_ISL_218443_A_canine_Korea_BD_1_2013_PA
EPI_ISL_329165_A_canine_Texas_2100186_2015_PA
EPI_ISL_503149_A_canine_Georgia_42632_2015_PA
EPI_ISL_284695_A_canine_Florida_269770_2015_PA
EPI_ISL_329184_A_canine_Georgia_101875_2015_PA
EPI_ISL_234607_A_canine_Georgia_10940_2015_PA
EPI_ISL_329166_A_canine_North_Carolina_103297_2015_PA
EPI_ISL_234610_A_canine_Georgia_95391_2015_PA
EPI_ISL_234609_A_canine_Indiana_96198_2015_PA
EPI_ISL_329164_A_canine_Illinois_278597_2016_PA
EPI_ISL_284697_A_canine_Illinois_283066_2015_PA
EPI_ISL_234606_A_canine_North_Carolina_109904_2015_PA
EPI_ISL_284694_A_canine_Indiana_003018_2016_PA
EPI_ISL_284698_A_canine_Illinois_328292_2015_PA
EPI_ISL_234669_A_canine_Texas_343907_2015_PA
EPI_ISL_234604_A_canine_Wisconsin_19137_2016_PA
EPI_ISL_308891_A_canine_Illinois_25374_19_1_2016_PA
EPI_ISL_329168_A_canine_Illinois_077752_2016_PA
EPI_ISL_284696_A_canine_Illinois_077753_2016_PA
EPI_ISL_329170_A_canine_Indiana_203681_2015_PA
EPI_ISL_329163_A_canine_Pennsylvania_026188S_2015_PA
EPI_ISL_329174_A_canine_Illinois_01823B9_2015_PA
EPI_ISL_329177_A_canine_Illinois_41915_2015_PA
EPI_ISL_190463_A_canine_Illinois_12191_2015_PA
EPI_ISL_284693_A_canine_South_Korea_017391S_2015_PA
EPI_ISL_234613_A_canine_Korea_058931B_2015_PA
EPI_ISL_234611_A_canine_Illinois_288370J_2015_PA
EPI_ISL_234608_A_canine_Illinois_161914A_2015_PA
EPI_ISL_234605_A_canine_Illinois_084093_2015_PA
EPI_ISL_329179_A_canine_Indiana_17031_16_2016_PA
EPI_ISL_329183_A_canine_Illinois_09137S_16_1_2016_PA
EPI_ISL_329175_A_canine_Indiana_09316S_16_1_2016_PA
EPI_ISL_308897_A_canine_Illinois_201701I0_2F1_2016_PA
EPI_ISL_308898_A_canine_Illinois_201701I0_1G4_2016_PA
EPI_ISL_329173_A_canine_Indiana_13227G_16_1_2_2016_PA
EPI_ISL_329167_A_canine_Illinois_201701I0_1H4_2016_PA
EPI_ISL_308895_A_canine_Illinois_201701I0_2G1_2016_PA
EPI_ISL_308886_A_canine_Illinois_201701I0_1G12_2016_PA
EPI_ISL_308882_A_canine_Illinois_18742J_16_7_2016_PA
EPI_ISL_178059B3_A_dog_Indiana_M16_09J78_32_1_2016_PA
EPI_ISL_329169_A_canine_Illinois_145135_16_3_1_2016_PA
EPI_ISL_308880_A_canine_Illinois_M16_3048O_3_1_2016_PA
EPI_ISL_308873_A_canine_Illinois_M16_2537H_14_1_2016_PA
EPI_ISL_308888_A_canine_Illinois_M16_16391_2_1_2016_PA
EPI_ISL_329176_A_canine_Indiana_1177_17_1_2017_PA
EPI_ISL_308885_A_canine_Pennsylvania_201701I1_5_2017_PA
EPI_ISL_308887_A_canine_Illinois_M17_0S782_11_1_2017_PA
EPI_ISL_308890_A_canine_Illinois_M17_0S782_4_1_2017_PA
EPI_ISL_308896_A_canine_Illinois_M17_0S782_7_1_2017_PA
EPI_ISL_308883_A_canine_Illinois_M17_0S782_1_1_2017_PA
EPI_ISL_308882_A_canine_Illinois_M17_0S782_8_1_2017_PA
EPI_ISL_308889_A_canine_Illinois_M17_0S782_1_1_2017_PA
EPI_ISL_308874_A_canine_South_Korea_201701I0_1F1_2016_PA
EPI_ISL_379502_A_canine_Nanjing_201704I9_12_2017_PA
EPI_ISL_379497_A_canine_Shanghai_201706Z2_7_2017_PA
EPI_ISL_379503_A_canine_Nanjing_201703Z8_7_2017_PA
EPI_ISL_379499_A_canine_Nanjing_201706Z0_47_2017_PA
EPI_ISL_379516_A_canine_Beijing_201707I9_15_2017_PA
EPI_ISL_379496_A_canine_Shanghai_201707I3_18_2017_PA
EPI_ISL_379526_A_canine_Beijing_201706I4_164_2017_PA
EPI_ISL_406244G_A_canine_China_QiaoKe01_2017_PA
EPI_ISL_379541_A_canine_Beijing_201703I4_73_2017_PA
EPI_ISL_379538_A_canine_Beijing_201704Z4_102_2017_PA
EPI_ISL_379505_A_canine_Beijing_20161228_9_2016_PA
EPI_ISL_379506_A_canine_Beijing_20161216_8_2016_PA
EPI_ISL_379537_A_canine_Beijing_201705O1_108_2017_PA
EPI_ISL_379522_A_canine_Beijing_201706I2_183_2017_PA
EPI_ISL_379539_A_canine_Beijing_201704I7_83_2017_PA
EPI_ISL_379514_A_canine_Beijing_201707I2_18_2017_PA
EPI_ISL_379494_A_canine_Xi_an_201706I1_157_2017_PA
EPI_ISL_379493_A_canine_Xi_an_201706I1_159_2017_PA
EPI_ISL_379527_A_canine_Beijing_201705I8_148_2017_PA
EPI_ISL_379540_A_canine_Beijing_201704I7_81_2017_PA
EPI_ISL_379519_A_canine_Beijing_201706I8_200_2017_PA
EPI_ISL_379517_A_canine_Beijing_201706I8_206_2017_PA
EPI_ISL_379520_A_canine_Beijing_201706I5_195_2017_PA
EPI_ISL_379523_A_canine_Beijing_201706I0_174_2017_PA
EPI_ISL_379525_A_canine_Beijing_201706I0_172_2017_PA
EPI_ISL_379524_A_canine_Beijing_201706I0_173_2017_PA
EPI_ISL_379521_A_canine_Beijing_201706I4_185_2017_PA
EPI_ISL_379518_A_canine_Beijing_201706I6_202_2017_PA
EPI_ISL_379534_A_canine_Beijing_201705I2_121_2017_PA
EPI_ISL_379533_A_canine_Beijing_201705I2_122_2017_PA
EPI_ISL_379495_A_canine_Shanghai_201707J1_36_2017_PA
EPI_ISL_678537G_A_canine_Henan_L03_2018_PA
EPI_ISL_678532_A_canine_Henan_L02_2018_PA
EPI_ISL_162079B3_A_canine_China_HN6_2018_PA
EPI_ISL_162079B0_A_canine_China_HN3_2018_PA
EPI_ISL_678534G_A_canine_Henan_L06_2018_PA
EPI_ISL_372298_A_canine_New_York_CRP034_2018_PA
EPI_ISL_372297_A_canine_New_Jersey_CRP036_2018_PA
EPI_ISL_372285_A_Canis_lupus_familiaris_USA_152386_2018_PA
EPI_ISL_372284_A_Canis_lupus_familiaris_USA_150620_2018_PA
EPI_ISL_372282_A_Canis_lupus_familiaris_USA_007783_2018_PA
EPI_ISL_372296_A_canine_Nevada_BRW018_2018_PA
EPI_ISL_502292_A_canine_Ontario_NCFAD_2018_070_1_2018_PA
EPI_ISL_502303_A_canine_Ontario_NCFAD_2018_070_5_2018_PA
EPI_ISL_372292_A_Canis_lupus_familiaris_USA_218592_2017_PA
EPI_ISL_372293_A_canine_California_BRW002_2018_PA
EPI_ISL_502301_A_canine_Ontario_NCFAD_2018_070_3_2018_PA
EPI_ISL_502300_A_canine_Ontario_NCFAD_2018_070_2_2018_PA
EPI_ISL_502302_A_canine_Ontario_NCFAD_2018_070_4_2018_PA
EPI_ISL_502286_A_canine_Ontario_NCFAD_2018_001_4_2018_PA
EPI_ISL_372295_A_canine_Nevada_BRW015_2018_PA
EPI_ISL_372294_A_canine_California_BRW003_2018_PA
EPI_ISL_372280_A_Canis_lupus_familiaris_USA_006974_2018_PA
EPI_ISL_308877_A_canine_Florida_201706O6_9_2017_PA
EPI_ISL_308876_A_canine_Florida_201706O6_18_2017_PA
EPI_ISL_329161_A_canine_North_Carolina_090487_2017_PA
EPI_ISL_329171_A_canine_Florida_090919_2017_PA
EPI_ISL_328524_A_canine_Florida_88807_2_2017_PA
EPI_ISL_308884_A_canine_Florida_201706O6_8_2017_PA
EPI_ISL_308894_A_canine_Florida_201706O6_2_2017_PA
EPI_ISL_308893_A_canine_Kentucky_201706O6_6_2017_PA
EPI_ISL_308878_A_canine_Florida_201706O6_17_2017_PA
EPI_ISL_308879_A_canine_Kentucky_201706O6_23_2017_PA
EPI_ISL_328523_A_canine_Georgia_89750_1_2017_PA
EPI_ISL_328522_A_canine_Georgia_201706O6_19_2017_PA
EPI_ISL_308892_A_canine_Florida_201706O6_11_2017_PA
EPI_ISL_308875_A_canine_Florida_201706O6_7_2017_PA
EPI_ISL_308881_A_canine_NorthCarolina_201706O6_28_2017_PA
EPI_ISL_329157_A_canine_Indiana_130636_2017_PA
EPI_ISL_329158_A_canine_Kentucky_128656_2017_PA
EPI_ISL_329154_A_canine_South_Carolina_132956_2017_PA
EPI_ISL_329160_A_canine_Minnesota_139684_2017_PA
EPI_ISL_372281_A_Canis_lupus_familiaris_USA_007780_2018_PA
EPI_ISL_372279_A_Canis_lupus_familiaris_USA_000915_2018_PA
EPI_ISL_329153_A_canine_Ohio_A97_2017_PA
EPI_ISL_372290_A_Canis_lupus_familiaris_USA_188297_2017_PA
EPI_ISL_329152_A_canine_Ohio_A91_2017_PA
EPI_ISL_329159_A_canine_Kentucky_174490_2017_PA
EPI_ISL_329172_A_canine_Illinois_A37_2017_PA
EPI_ISL_372287_A_Canis_lupus_familiaris_USA_118191_2017_PA
EPI_ISL_372289_A_Canis_lupus_familiaris_USA_188226_2017_PA
EPI_ISL_372291_A_Canis_lupus_familiaris_USA_188305_2017_PA
EPI_ISL_329162_A_canine_Ohio_174509_2017_PA
EPI_ISL_372288_A_Canis_lupus_familiaris_USA_188203_2017_PA
EPI_ISL_329151_A_canine_Ohio_A82_2017_PA
EPI_ISL_379515_A_canine_Beijing_201707I1_16_2017_PA
EPI_ISL_379501_A_canine_Nanjing_201705I5_25_2017_PA
EPI_ISL_379500_A_canine_Nanjing_201706O7_40_2017_PA
EPI_ISL_379498_A_canine_Nanjing_201707O4_53_2017_PA
EPI_ISL_328526_A_canine_California_84315_2017_PA
EPI_ISL_379535_A_canine_Beijing_201705I2_120_2017_PA
EPI_ISL_379530_A_canine_Beijing_201705I7_146_2017_PA
EPI_ISL_379529_A_canine_Beijing_201705I8_147_2017_PA
EPI_ISL_379536_A_canine_Beijing_201705I0_118_2017_PA
EPI_ISL_379532_A_canine_Beijing_201706I2_133_2017_PA
EPI_ISL_379531_A_canine_Beijing_201706I2_137_2017_PA
EPI_ISL_502296_A_canine_Ontario_NCFAD_2018_070_13_2018_PA
EPI_ISL_502297_A_canine_Ontario_NCFAD_2018_070_14_2018_PA
EPI_ISL_502299_A_canine_Ontario_NCFAD_2018_070_16_2018_PA
EPI_ISL_502298_A_canine_Ontario_NCFAD_2018_070_15_2018_PA
EPI_ISL_502295_A_canine_Ontario_NCFAD_2018_070_12_2018_PA
EPI_ISL_502294_A_canine_Ontario_NCFAD_2018_070_11_2018_PA
EPI_ISL_502306_A_canine_Ontario_NCFAD_2018_070_9_2018_PA
EPI_ISL_502304_A_canine_Ontario_NCFAD_2018_070_7_2018_PA
EPI_ISL_502305_A_canine_Ontario_NCFAD_2018_070_8_2018_PA
EPI_ISL_502293_A_canine_Ontario_NCFAD_2018_070_10_2018_PA
EPI_ISL_379513_A_canine_Beijing_201707I2O_30_2017_PA
EPI_ISL_148746O1_A_canine_Singapore_SG_NParks_CIV_M1030S_2018_PA
EPI_ISL_148746O1_A_canine_Singapore_SG_NParks_CIV_M1040S_2018_PA
EPI_ISL_499084_A_canine_Guangdong_1_2018_PA
EPI_ISL_499085_A_canine_Guangdong_2_2018_PA
EPI_ISL_678535G_A_canine_Guangdong_GY01_2018_PA
EPI_ISL_162078G9_A_canine_China_11071_2019_PA
EPI_ISL_174772I2_A_canine_Beijing_1016_2018_PA
EPI_ISL_366884_A_canine_China_Shanghai_0315_2019_2019_PA
EPI_ISL_187501O8_A_canine_California_CVM_173841I_2019_PA
EPI_ISL_187501O7_A_canine_California_CVM_173841O1_2019_PA
EPI_ISL_502288_A_canine_Ontario_NCFAD_2018_069_1_2018_PA
EPI_ISL_502290_A_canine_Ontario_NCFAD_2018_069_6_2018_PA
EPI_ISL_502291_A_canine_Ontario_NCFAD_2018_069_9_2018_PA
EPI_ISL_502289_A_canine_Ontario_NCFAD_2018_069_10_2018_PA
EPI_ISL_502287_A_canine_Ontario_NCFAD_2018_064_1_2018_PA
EPI_ISL_499086_A_canine_Guangdong_3_2018_PA
EPI_ISL_172772G5_A_canine_China_6068_2018_PA
CN_HLJ_1_18_H3N2__PA
CN_HLJ_1_19_H3N2__PA
EPI_ISL_172772G6_A_canine_China_15001_2019_PA
EPI_ISL_172773G5_A_canine_China_15027_2019_PA
EPI_ISL_172773G7_A_canine_China_15068_2019_PA
EPI_ISL_172773G6_A_canine_China_15066_2019_PA
CN_HLJ_3_19_H3N2__PA
EPI_ISL_406192S_A_canine_Texas_21_011409_001_2021_PA
CN_HLJ_2_19_H3N2__PA
EPI_ISL_403157G_A_canine_Guangdong_DY1_2019_PA
EPI_ISL_186908O2_A_canine_Hainan_CAU_W17_2015_PA
EPI_ISL_187506I6_A_canine_California_CVM_321511O1_2021_PA
EPI_ISL_187507J3_A_canine_Florida_CVM_H1_2021_PA
EPI_ISL_416803G_A_canine_California_LACPHL_INF00006_2021_PA
EPI_ISL_187507J2_A_canine_Florida_CVM_F1_2021_PA
EPI_ISL_186907P9_A_canine_Beijing_CAU_S11_2011_PA
EPI_ISL_186907P8_A_canine_Beijing_CAU_S10_2010_PA
EPI_ISL_187507G8_A_canine_California_CVM_339542G1_2021_PA
EPI_ISL_186907P7_A_canine_Beijing_CAU_S01_2001_PA
EPI_ISL_186907P6_A_canine_Beijing_CAU_118_2018_PA
EPI_ISL_187512S7_A_canine_Texas_CVM_093364I_2023_PA
EPI_ISL_187512S8_A_canine_Texas_CVM_093644I_2023_PA
EPI_ISL_187512S9_A_canine_Texas_CVM_093365I_2023_PA
EPI_ISL_187522O6_A_canine_Pennsylvania_03775G_2023_PA
EPI_ISL_187512S4_A_canine_Texas_CVM_093361I_2023_PA
EPI_ISL_187522O7_A_canine_Tennessee_CVM_231361_2022_PA
EPI_ISL_187512G5_A_canine_Texas_CVM_020417I_2023_PA
EPI_ISL_176932J5_A_canine_Oklahoma_011I24I_2023_PA
EPI_ISL_187512S2_A_canine_Texas_CVM_068804I_2023_PA
EPI_ISL_187512G1_A_canine_West_Virginia_CVM_120S0B_1_2023_PA
EPI_ISL_187512G0_A_canine_Virginia_CVM_00291I1_2023_PA
EPI_ISL_187507J4_A_canine_Pennsylvania_CVM_9B5419_2023_PA
EPI_ISL_187522O9_A_canine_Texas_CVM_042018_3_2023_PA
EPI_ISL_187512G_A_canine_Texas_CVM_020415I_2023_PA
EPI_ISL_187507J5_A_canine_Texas_CVM_37B54I1_2022_PA
EPI_ISL_187512S5_A_canine_Texas_CVM_093362I_2023_PA
EPI_ISL_187508S3_A_canine_Texas_CVM_068802I_2023_PA
EPI_ISL_187522O5_A_canine_Alabama_CVM_194456_1_2022_PA
EPI_ISL_187508S1_A_canine_Texas_CVM_049142I_2023_PA
EPI_ISL_187512I7_A_canine_Texas_CVM_042018Z_2023_PA
EPI_ISL_187508S2_A_canine_Texas_CVM_049143I_2023_PA
EPI_ISL_187512S3_A_canine_Texas_CVM_068807I_2023_PA
EPI_ISL_187512S6_A_canine_Minnesota_012B4_2023_PA
E
1
2
3
4
5

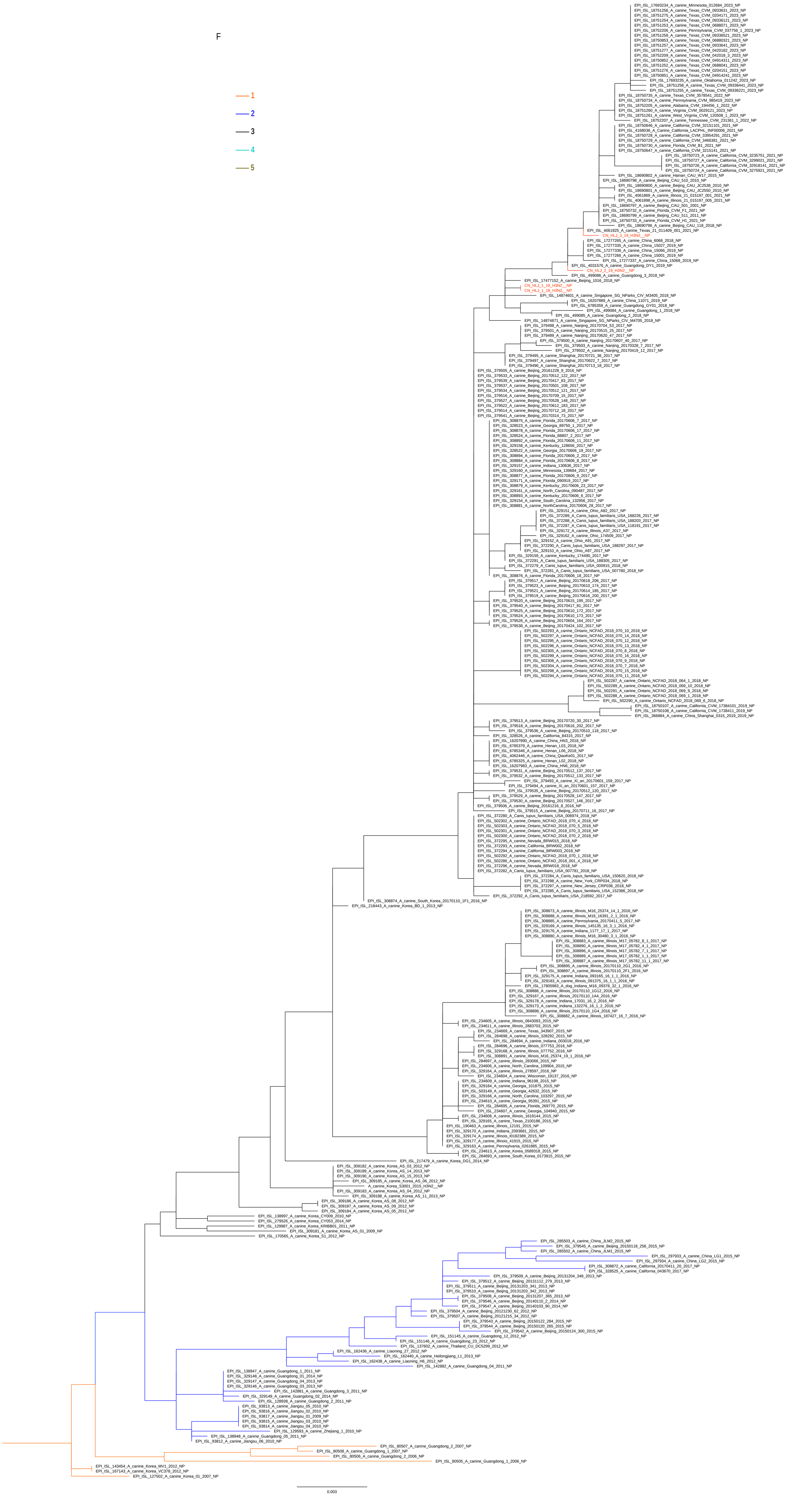
F
1
2
3
4
5
EPI_ISL_17693234_A_canine_Minnesota_012684_2023_NP
EPI_ISL_18751256_A_canine_Texas_CVM_0933631_2023_NP
EPI_ISL_18751275_A_canine_Texas_CVM_0204171_2023_NP
EPI_ISL_18751254_A_canine_Texas_CVM_09336121_2023_NP
EPI_ISL_18751253_A_canine_Texas_CVM_0688071_2023_NP
EPI_ISL_18752206_A_canine_Pennsylvania_CVM_037756_1_2023_NP
EPI_ISL_18751259_A_canine_Texas_CVM_09336521_2023_NP
EPI_ISL_18750853_A_canine_Texas_CVM_0688032I_2023_NP
EPI_ISL_18751257_A_canine_Texas_CVM_0933641_2023_NP
EPI_ISL_18751277_A_canine_Texas_CVM_0420182_2023_NP
EPI_ISL_18752209_A_canine_Texas_CVM_042018_3_2023_NP
EPI_ISL_18750852_A_canine_Texas_CVM_04914311_2023_NP
EPI_ISL_18751252_A_canine_Texas_CVM_0688041_2023_NP
EPI_ISL_18751276_A_canine_Texas_CVM_0204151_2023_NP
EPI_ISL_18750851_A_canine_Texas_CVM_04914241_2023_NP
EPI_ISL_17693235_A_canine_Oklahoma_011242_2023_NP
EPI_ISL_18751258_A_canine_Texas_CVM_09336441_2023_NP
EPI_ISL_18751255_A_canine_Texas_CVM_09336221_2023_NP
EPI_ISL_18750735_A_canine_Texas_CVM_3578541_2022_NP
EPI_ISL_18750734_A_canine_Pennsylvania_CVM_985419_2023_NP
EPI_ISL_18752205_A_canine_Alabama_CVM_194456_1_2022_NP
EPI_ISL_18751260_A_canine_Virginia_CVM_0029121_2023_NP
EPI_ISL_18751261_A_canine_West_Virginia_CVM_1200508_1_2023_NP
EPI_ISL_18752207_A_canine_Tennessee_CVM_231361_1_2022_NP
EPI_ISL_18750646_A_canine_California_CVM_32151101_2021_NP
EPI_ISL_4168036_A_Canine_California_LACPHL_INF00006_2021_NP
EPI_ISL_18750728_A_canine_California_CVM_33954291_2021_NP
EPI_ISL_18750729_A_canine_California_CVM_3466381_2021_NP
EPI_ISL_18750730_A_canine_Florida_CVM_B1_2021_NP
EPI_ISL_18750647_A_canine_California_CVM_3215141_2021_NP
EPI_ISL_18750723_A_canine_California_CVM_3235751_2021_NP
EPI_ISL_18750727_A_canine_California_CVM_3299021_2021_NP
EPI_ISL_18750726_A_canine_California_CVM_32918141_2021_NP
EPI_ISL_18750724_A_canine_California_CVM_3275921_2021_NP
EPI_ISL_18690802_A_canine_Hainan_CAU_W17_2015_NP
EPI_ISL_18690798_A_canine_Beijing_CAU_510_2010_NP
EPI_ISL_18690800_A_canine_Beijing_CAU_JC2538_2010_NP
EPI_ISL_18690801_A_canine_Beijing_CAU_JC2550_2010_NP
EPI_ISL_4061869_A_canine_Illinois_21_015197_001_2021_NP
EPI_ISL_4061898_A_canine_Illinois_21_015197_005_2021_NP
EPI_ISL_18690797_A_canine_Beijing_CAU_501_2001_NP
EPI_ISL_18750732_A_canine_Florida_CVM_F1_2021_NP
EPI_ISL_18690799_A_canine_Beijing_CAU_511_2011_NP
EPI_ISL_18750733_A_canine_Florida_CVM_H1_2021_NP
EPI_ISL_18690796_A_canine_Beijing_CAU_118_2018_NP
EPI_ISL_4061925_A_canine_Texas_21_011409_001_2021_NP
CN_HLJ_3_19_H3N2__NP
EPI_ISL_17277265_A_canine_China_6068_2018_NP
EPI_ISL_17277335_A_canine_China_15027_2019_NP
EPI_ISL_17277336_A_canine_China_15066_2019_NP
EPI_ISL_17277266_A_canine_China_15001_2019_NP
EPI_ISL_17277337_A_canine_China_15068_2019_NP
EPI_ISL_4031576_A_canine_Guangdong_DY1_2019_NP
CN_HLJ_2_19_H3N2__NP
EPI_ISL_499086_A_canine_Guangdong_3_2018_NP
EPI_ISL_17477152_A_canine_Beijing_1016_2018_NP
CN_HLJ_1_19_H3N2__NP
CN_HLJ_1_18_H3N2__NP
EPI_ISL_14874601_A_canine_Singapore_SG_NParks_CIV_M3405_2018_NP
EPI_ISL_16207889_A_canine_China_11071_2019_NP
EPI_ISL_6785359_A_canine_Guangdong_GY01_2018_NP
EPI_ISL_499084_A_canine_Guangdong_1_2018_NP
EPI_ISL_499085_A_canine_Guangdong_2_2018_NP
EPI_ISL_14874671_A_canine_Singapore_SG_NParks_CIV_M4705_2018_NP
EPI_ISL_379498_A_canine_Nanjing_20170704_53_2017_NP
EPI_ISL_379501_A_canine_Nanjing_20170515_25_2017_NP
EPI_ISL_379499_A_canine_Nanjing_20170620_47_2017_NP
EPI_ISL_379500_A_canine_Nanjing_20170607_40_2017_NP
EPI_ISL_379503_A_canine_Nanjing_20170328_7_2017_NP
EPI_ISL_379502_A_canine_Nanjing_20170419_12_2017_NP
EPI_ISL_379495_A_canine_Shanghai_20170721_36_2017_NP
EPI_ISL_379497_A_canine_Shanghai_20170622_7_2017_NP
EPI_ISL_379496_A_canine_Shanghai_20170713_18_2017_NP
EPI_ISL_379505_A_canine_Beijing_20161228_9_2016_NP
EPI_ISL_379533_A_canine_Beijing_20170512_122_2017_NP
EPI_ISL_379539_A_canine_Beijing_20170417_83_2017_NP
EPI_ISL_379537_A_canine_Beijing_20170501_108_2017_NP
EPI_ISL_379534_A_canine_Beijing_20170512_121_2017_NP
EPI_ISL_379516_A_canine_Beijing_20170709_15_2017_NP
EPI_ISL_379527_A_canine_Beijing_20170528_148_2017_NP
EPI_ISL_379522_A_canine_Beijing_20170612_183_2017_NP
EPI_ISL_379514_A_canine_Beijing_20170712_18_2017_NP
EPI_ISL_379541_A_canine_Beijing_20170314_73_2017_NP
EPI_ISL_308875_A_canine_Florida_20170606_7_2017_NP
EPI_ISL_328523_A_canine_Georgia_89750_1_2017_NP
EPI_ISL_308878_A_canine_Florida_20170606_17_2017_NP
EPI_ISL_328524_A_canine_Florida_88807_2_2017_NP
EPI_ISL_308892_A_canine_Florida_20170606_11_2017_NP
EPI_ISL_329158_A_canine_Kentucky_128656_2017_NP
EPI_ISL_328522_A_canine_Georgia_20170606_19_2017_NP
EPI_ISL_308894_A_canine_Florida_20170606_2_2017_NP
EPI_ISL_308884_A_canine_Florida_20170606_8_2017_NP
EPI_ISL_329157_A_canine_Indiana_130636_2017_NP
EPI_ISL_329160_A_canine_Minnesota_139684_2017_NP
EPI_ISL_308877_A_canine_Florida_20170606_9_2017_NP
EPI_ISL_329171_A_canine_Florida_090919_2017_NP
EPI_ISL_308879_A_canine_Kentucky_20170606_23_2017_NP
EPI_ISL_329161_A_canine_North_Carolina_090487_2017_NP
EPI_ISL_308893_A_canine_Kentucky_20170606_6_2017_NP
EPI_ISL_329154_A_canine_South_Carolina_132956_2017_NP
EPI_ISL_308881_A_canine_NorthCarolina_20170606_28_2017_NP
EPI_ISL_329151_A_canine_Ohio_A82_2017_NP
EPI_ISL_372289_A_Canis_lupus_familiaris_USA_188226_2017_NP
EPI_ISL_372288_A_Canis_lupus_familiaris_USA_188203_2017_NP
EPI_ISL_372287_A_Canis_lupus_familiaris_USA_118191_2017_NP
EPI_ISL_329172_A_canine_Illinois_A37_2017_NP
EPI_ISL_329162_A_canine_Ohio_174509_2017_NP
EPI_ISL_329152_A_canine_Ohio_A91_2017_NP
EPI_ISL_372290_A_Canis_lupus_familiaris_USA_188297_2017_NP
EPI_ISL_329153_A_canine_Ohio_A97_2017_NP
EPI_ISL_329159_A_canine_Kentucky_174490_2017_NP
EPI_ISL_372291_A_Canis_lupus_familiaris_USA_188305_2017_NP
EPI_ISL_372279_A_Canis_lupus_familiaris_USA_000915_2018_NP
EPI_ISL_372281_A_Canis_lupus_familiaris_USA_007780_2018_NP
EPI_ISL_308876_A_canine_Florida_20170606_18_2017_NP
EPI_ISL_379517_A_canine_Beijing_20170618_206_2017_NP
EPI_ISL_379523_A_canine_Beijing_20170610_174_2017_NP
EPI_ISL_379521_A_canine_Beijing_20170614_185_2017_NP
EPI_ISL_379519_A_canine_Beijing_20170616_200_2017_NP
EPI_ISL_379520_A_canine_Beijing_20170615_195_2017_NP
EPI_ISL_379540_A_canine_Beijing_20170417_81_2017_NP
EPI_ISL_379525_A_canine_Beijing_20170610_172_2017_NP
EPI_ISL_379524_A_canine_Beijing_20170610_173_2017_NP
EPI_ISL_379526_A_canine_Beijing_20170604_164_2017_NP
EPI_ISL_379538_A_canine_Beijing_20170424_102_2017_NP
EPI_ISL_502293_A_canine_Ontario_NCFAD_2018_070_10_2018_NP
EPI_ISL_502297_A_canine_Ontario_NCFAD_2018_070_14_2018_NP
EPI_ISL_502295_A_canine_Ontario_NCFAD_2018_070_12_2018_NP
EPI_ISL_502296_A_canine_Ontario_NCFAD_2018_070_13_2018_NP
EPI_ISL_502305_A_canine_Ontario_NCFAD_2018_070_8_2018_NP
EPI_ISL_502299_A_canine_Ontario_NCFAD_2018_070_16_2018_NP
EPI_ISL_502306_A_canine_Ontario_NCFAD_2018_070_9_2018_NP
EPI_ISL_502304_A_canine_Ontario_NCFAD_2018_070_7_2018_NP
EPI_ISL_502298_A_canine_Ontario_NCFAD_2018_070_15_2018_NP
EPI_ISL_502294_A_canine_Ontario_NCFAD_2018_070_11_2018_NP
EPI_ISL_502287_A_canine_Ontario_NCFAD_2018_064_1_2018_NP
EPI_ISL_502289_A_canine_Ontario_NCFAD_2018_069_10_2018_NP
EPI_ISL_502291_A_canine_Ontario_NCFAD_2018_069_9_2018_NP
EPI_ISL_502288_A_canine_Ontario_NCFAD_2018_069_1_2018_NP
EPI_ISL_502290_A_canine_Ontario_NCFAD_2018_069_6_2018_NP
EPI_ISL_18750107_A_canine_California_CVM_17384101_2019_NP
EPI_ISL_18750108_A_canine_California_CVM_1738411_2019_NP
EPI_ISL_366884_A_canine_China_Shanghai_0315_2019_NP
EPI_ISL_379513_A_canine_Beijing_20170720_30_2017_NP
EPI_ISL_379518_A_canine_Beijing_20170616_202_2017_NP
EPI_ISL_379536_A_canine_Beijing_20170510_118_2017_NP
EPI_ISL_328526_A_canine_California_84315_2017_NP
EPI_ISL_16207890_A_canine_China_HN3_2018_NP
EPI_ISL_6785379_A_canine_Henan_L03_2018_NP
EPI_ISL_6785346_A_canine_Henan_L06_2018_NP
EPI_ISL_4062446_A_canine_ChaoKe401_2017_NP
EPI_ISL_6785325_A_canine_Henan_L02_2018_NP
EPI_ISL_16207983_A_canine_China_HN6_2018_NP
EPI_ISL_379531_A_canine_Beijing_20170512_137_2017_NP
EPI_ISL_379532_A_canine_Beijing_20170512_133_2017_NP
EPI_ISL_379493_A_canine_Xi_an_20170601_159_2017_NP
EPI_ISL_379494_A_canine_Xi_an_20170601_157_2017_NP
EPI_ISL_379535_A_canine_Beijing_20170512_120_2017_NP
EPI_ISL_379529_A_canine_Beijing_20170528_147_2017_NP
EPI_ISL_379530_A_canine_Beijing_20170527_146_2017_NP
EPI_ISL_379506_A_canine_Beijing_20161216_8_2016_NP
EPI_ISL_379511_A_canine_Beijing_20170711_16_2017_NP
EPI_ISL_372280_A_Canis_lupus_familiaris_USA_006974_2018_NP
EPI_ISL_502302_A_canine_Ontario_NCFAD_2018_070_4_2018_NP
EPI_ISL_502303_A_canine_Ontario_NCFAD_2018_070_5_2018_NP
EPI_ISL_502301_A_canine_Ontario_NCFAD_2018_070_3_2018_NP
EPI_ISL_502300_A_canine_Ontario_NCFAD_2018_070_2_2018_NP
EPI_ISL_372295_A_canine_Nevada_BRW015_2018_NP
EPI_ISL_372293_A_canine_California_BRW002_2018_NP
EPI_ISL_372294_A_canine_California_BRW003_2018_NP
EPI_ISL_502292_A_canine_Ontario_NCFAD_2018_070_1_2018_NP
EPI_ISL_502286_A_canine_Ontario_NCFAD_2018_001_4_2018_NP
EPI_ISL_372296_A_canine_Nevada_BRW018_2018_NP
EPI_ISL_372282_A_Canis_lupus_familiaris_USA_007781_2018_NP
EPI_ISL_372284_A_Canis_lupus_familiaris_USA_150620_2018_NP
EPI_ISL_372298_A_canine_New_York_CRP034_2018_NP
EPI_ISL_372297_A_canine_New_Jersey_CRP036_2018_NP
EPI_ISL_372285_A_Canis_lupus_familiaris_USA_152386_2018_NP
EPI_ISL_372292_A_Canis_lupus_familiaris_USA_218592_2017_NP
EPI_ISL_308874_A_canine_South_Korea_20170110_1F1_2016_NP
EPI_ISL_218443_A_canine_Korea_BD_1_2013_NP
EPI_ISL_308873_A_canine_Illinois_M16_25374_14_1_2016_NP
EPI_ISL_308888_A_canine_Illinois_M16_16391_2_1_2016_NP
EPI_ISL_308885_A_canine_Pennsylvania_20170411_5_2017_NP
EPI_ISL_329169_A_canine_Illinois_145135_16_3_1_2016_NP
EPI_ISL_329176_A_canine_Indiana_1177_17_1_2017_NP
EPI_ISL_308880_A_canine_Illinois_M16_30480_3_1_2016_NP
EPI_ISL_308883_A_canine_Illinois_M17_05782_8_1_2017_NP
EPI_ISL_308890_A_canine_Illinois_M17_05782_4_1_2017_NP
EPI_ISL_308896_A_canine_Illinois_M17_05782_7_1_2017_NP
EPI_ISL_308889_A_canine_Illinois_M17_05782_1_1_2017_NP
EPI_ISL_308887_A_canine_Illinois_M17_05782_11_1_2017_NP
EPI_ISL_308895_A_canine_Illinois_20170110_2G1_2016_NP
EPI_ISL_308897_A_canine_Illinois_20170110_2F1_2016_NP
EPI_ISL_329175_A_canine_Indiana_093165_16_1_1_2016_NP
EPI_ISL_329183_A_canine_Illinois_091375_16_1_1_2016_NP
EPI_ISL_17805983_A_dog_Indiana_M16_09378_32_1_2016_NP
EPI_ISL_308886_A_canine_Illinois_20170110_1G12_2016_NP
EPI_ISL_329167_A_canine_Illinois_20170110_1A4_2016_NP
EPI_ISL_329178_A_canine_Indiana_17031_16_2_2016_NP
EPI_ISL_329173_A_canine_Indiana_132276_16_1_2_2016_NP
EPI_ISL_308898_A_canine_Illinois_20170110_1G4_2016_NP
EPI_ISL_308882_A_canine_Illinois_187427_16_7_2016_NP
EPI_ISL_234605_A_canine_Illinois_0843093_2015_NP
EPI_ISL_234611_A_canine_Illinois_2883703_2015_NP
EPI_ISL_234669_A_canine_Texas_343907_2015_NP
EPI_ISL_284698_A_canine_Illinois_328292_2015_NP
EPI_ISL_284694_A_canine_Indiana_003018_2016_NP
EPI_ISL_284696_A_canine_Illinois_077753_2016_NP
EPI_ISL_329168_A_canine_Illinois_077752_2016_NP
EPI_ISL_308891_A_canine_Illinois_M16_25374_19_1_2016_NP
EPI_ISL_284697_A_canine_Illinois_283066_2015_NP
EPI_ISL_234606_A_canine_North_Carolina_1099904_2015_NP
EPI_ISL_329164_A_canine_Illinois_278597_2016_NP
EPI_ISL_234604_A_canine_Wisconsin_19137_2016_NP
EPI_ISL_234609_A_canine_Indiana_96198_2015_NP
EPI_ISL_329184_A_canine_Georgia_101875_2015_NP
EPI_ISL_503149_A_canine_Georgia_42632_2015_NP
EPI_ISL_329166_A_canine_North_Carolina_103297_2015_NP
EPI_ISL_234610_A_canine_Georgia_95391_2015_NP
EPI_ISL_284695_A_canine_Florida_269770_2015_NP
EPI_ISL_234607_A_canine_Georgia_104940_2015_NP
EPI_ISL_234608_A_canine_Illinois_1619144_2015_NP
EPI_ISL_329165_A_canine_Texas_2100186_2015_NP
EPI_ISL_190463_A_canine_Illinois_12191_2015_NP
EPI_ISL_329170_A_canine_Indiana_2093681_2015_NP
EPI_ISL_329174_A_canine_Illinois_01828389_2015_NP
EPI_ISL_329177_A_canine_Illinois_41915_2015_NP
EPI_ISL_329163_A_canine_Pennsylvania_02618805_2015_NP
EPI_ISL_234613_A_canine_Korea_0589318_2015_NP
EPI_ISL_284693_A_canine_South_Korea_0173915_2015_NP
EPI_ISL_217479_A_canine_Korea_DG1_2014_NP
EPI_ISL_309182_A_canine_Korea_AS_03_2012_NP
EPI_ISL_309189_A_canine_Korea_AS_14_2013_NP
EPI_ISL_309190_A_canine_Korea_AS_15_2013_NP
EPI_ISL_309185_A_canine_Korea_AS_06_2012_NP
A_canine_Korea_S3001_2015_H3N2__NP
EPI_ISL_309183_A_canine_Korea_AS_04_2012_NP
EPI_ISL_309188_A_canine_Korea_AS_11_2013_NP
EPI_ISL_309186_A_canine_Korea_AS_08_2012_NP
EPI_ISL_309187_A_canine_Korea_AS_09_2012_NP
EPI_ISL_309184_A_canine_Korea_AS_05_2012_NP
EPI_ISL_138997_A_canine_Korea_CY009_2010_NP
EPI_ISL_279526_A_canine_Korea_CY053_2014_NP
EPI_ISL_129987_A_canine_Korea_KR8801_2011_NP
EPI_ISL_309181_A_canine_Korea_AS_01_2009_NP
EPI_ISL_170565_A_canine_Korea_S1_2012_NP
EPI_ISL_285503_A_canine_China_JLM2_2015_NP
EPI_ISL_379545_A_canine_Beijing_20150118_256_2015_NP
EPI_ISL_285502_A_canine_China_JLM1_2015_NP
EPI_ISL_297933_A_canine_China_LG1_2015_NP
EPI_ISL_297934_A_canine_China_LG2_2015_NP
EPI_ISL_308872_A_canine_California_20170411_20_2017_NP
EPI_ISL_328525_A_canine_California_043670_2017_NP
EPI_ISL_379509_A_canine_Beijing_20131204_349_2013_NP
EPI_ISL_379512_A_canine_Beijing_20131112_279_2013_NP
EPI_ISL_379511_A_canine_Beijing_20131203_341_2013_NP
EPI_ISL_379510_A_canine_Beijing_20131203_342_2013_NP
EPI_ISL_379508_A_canine_Beijing_20131207_365_2013_NP
EPI_ISL_379546_A_canine_Beijing_20140110_2_2014_NP
EPI_ISL_379547_A_canine_Beijing_20140103_90_2014_NP
EPI_ISL_379504_A_canine_Beijing_20121230_62_2012_NP
EPI_ISL_379507_A_canine_Beijing_20121215_34_2012_NP
EPI_ISL_379543_A_canine_Beijing_20150122_284_2015_NP
EPI_ISL_379544_A_canine_Beijing_20150120_265_2015_NP
EPI_ISL_379542_A_canine_Beijing_20150124_300_2015_NP
EPI_ISL_151146_A_canine_Guangdong_23_2012_NP
EPI_ISL_151145_A_canine_Guangdong_12_2012_NP
EPI_ISL_137602_A_canine_Thailand_CU_DC5299_2012_NP
EPI_ISL_162436_A_canine_Liaoning_27_2012_NP
EPI_ISL_162440_A_canine_Heilongjiang_L1_2013_NP
EPI_ISL_162438_A_canine_Liaoning_H6_2012_NP
EPI_ISL_142882_A_canine_Guangdong_04_2011_NP
EPI_ISL_138947_A_canine_Guangdong_1_2011_NP
EPI_ISL_329148_A_canine_Guangdong_01_2014_NP
EPI_ISL_329147_A_canine_Guangdong_04_2013_NP
EPI_ISL_329146_A_canine_Guangdong_03_2013_NP
EPI_ISL_142881_A_canine_Guangdong_3_2011_NP
EPI_ISL_329149_A_canine_Guangdong_02_2014_NP
EPI_ISL_128938_A_canine_Guangdong_2_2011_NP
EPI_ISL_93813_A_canine_Jiangsu_05_2010_NP
EPI_ISL_93816_A_canine_Jiangsu_02_2010_NP
EPI_ISL_93817_A_canine_Jiangsu_01_2009_NP
EPI_ISL_93815_A_canine_Jiangsu_03_2010_NP
EPI_ISL_93814_A_canine_Jiangsu_04_2010_NP
EPI_ISL_129593_A_canine_Zhejiang_1_2010_NP
EPI_ISL_138948_A_canine_Guangdong_05_2011_NP
EPI_ISL_93812_A_canine_Jiangsu_06_2010_NP
EPI_ISL_80507_A_canine_Guangdong_2_2007_NP
EPI_ISL_80508_A_canine_Guangdong_1_2007_NP
EPI_ISL_80506_A_canine_Guangdong_2_2006_NP
EPI_ISL_80505_A_canine_Guangdong_1_2006_NP
EPI_ISL_143454_A_canine_Korea_MV1_2012_NP
EPI_ISL_167143_A_canine_Korea_YC378_2012_NP
EPI_ISL_127502_A_canine_Korea_01_2007_NP
0.003

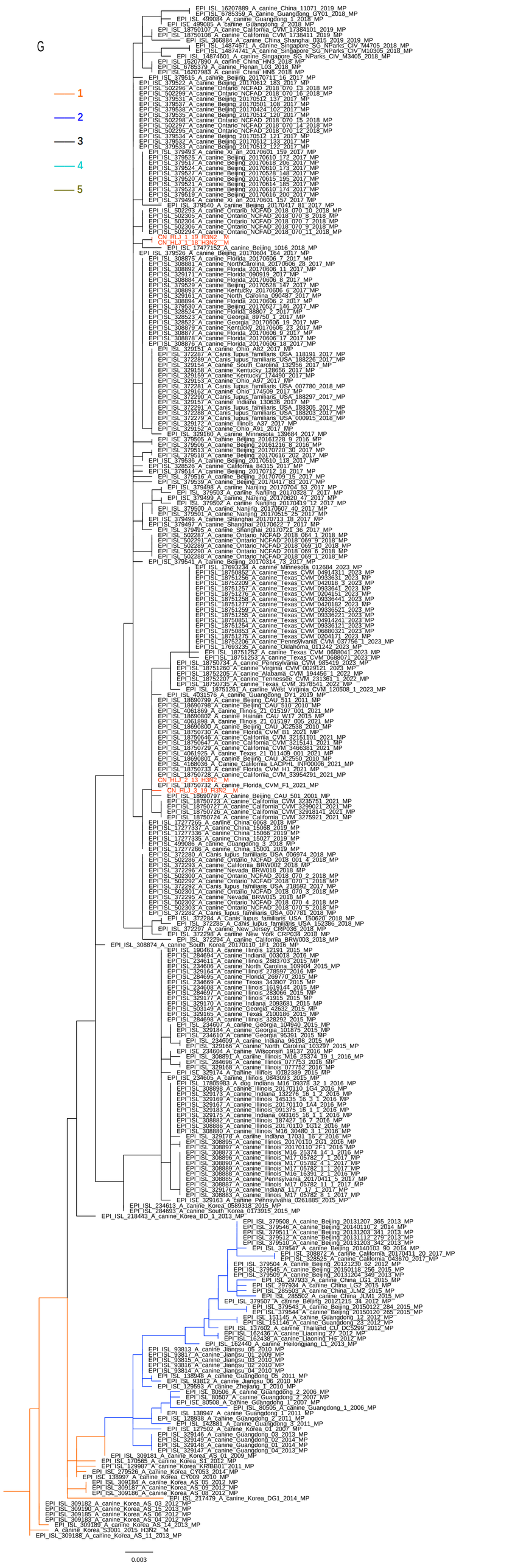
G
1
2
3
4
5
EPI_ISL_16207889_A_canine_China_11071_2019_MP
EPI_ISL_6785359_A_canine_Guangdong_GY01_2018_MP
EPI_ISL_499083_A_canine_Guangdong_1_2018_MP
EPI_ISL_499085_A_canine_Guangdong_2_2018_MP
EPI_ISL_18750107_A_canine_California_CVM_17384101_2019_MP
EPI_ISL_18750108_A_canine_California_CVM_17384111_2019_MP
EPI_ISL_16208844_A_canine_China_Shanghai_03115_2019_2019_MP
EPI_ISL_14874671_A_canine_Singapore_SG_NParks_CIV_M4705_2018_MP
EPI_ISL_14874741_A_canine_Singapore_SG_NParks_CIV_M10305_2018_MP
EPI_ISL_14874601_A_canine_Singapore_SG_NParks_CIV_M3405_2018_MP
EPI_ISL_16207890_A_canine_China_HN3_2018_MP
EPI_ISL_6785379_A_canine_Henan_L03_2018_MP
EPI_ISL_16207983_A_canine_China_HN6_2018_MP
EPI_ISL_379515_A_canine_Beijing_20170711_16_2017_MP
EPI_ISL_379522_A_canine_Beijing_20170612_183_2017_MP
EPI_ISL_502296_A_canine_Ontario_NCFAD_2018_070_13_2018_MP
EPI_ISL_502299_A_canine_Ontario_NCFAD_2018_070_16_2018_MP
EPI_ISL_379531_A_canine_Beijing_20170512_137_2017_MP
EPI_ISL_379537_A_canine_Beijing_20170501_108_2017_MP
EPI_ISL_379538_A_canine_Beijing_20170424_102_2017_MP
EPI_ISL_379535_A_canine_Beijing_20170512_120_2017_MP
EPI_ISL_502298_A_canine_Ontario_NCFAD_2018_070_15_2018_MP
EPI_ISL_502297_A_canine_Ontario_NCFAD_2018_070_14_2018_MP
EPI_ISL_502295_A_canine_Ontario_NCFAD_2018_070_12_2018_MP
EPI_ISL_379534_A_canine_Beijing_20170512_121_2017_MP
EPI_ISL_379532_A_canine_Beijing_20170512_133_2017_MP
EPI_ISL_379533_A_canine_Beijing_20170512_122_2017_MP
EPI_ISL_379493_A_canine_Xi_an_20170601_159_2017_MP
EPI_ISL_379525_A_canine_Beijing_20170610_172_2017_MP
EPI_ISL_379517_A_canine_Beijing_20170618_206_2017_MP
EPI_ISL_379524_A_canine_Beijing_20170610_173_2017_MP
EPI_ISL_379527_A_canine_Beijing_20170528_148_2017_MP
EPI_ISL_379520_A_canine_Beijing_20170615_195_2017_MP
EPI_ISL_379521_A_canine_Beijing_20170614_185_2017_MP
EPI_ISL_379523_A_canine_Beijing_20170610_174_2017_MP
EPI_ISL_379519_A_canine_Beijing_20170616_200_2017_MP
EPI_ISL_379494_A_canine_Xi_an_20170601_157_2017_MP
EPI_ISL_379540_A_canine_Beijing_20170417_81_2017_MP
EPI_ISL_502293_A_canine_Ontario_NCFAD_2018_070_10_2018_MP
EPI_ISL_502305_A_canine_Ontario_NCFAD_2018_070_8_2018_MP
EPI_ISL_502304_A_canine_Ontario_NCFAD_2018_070_7_2018_MP
EPI_ISL_502306_A_canine_Ontario_NCFAD_2018_070_9_2018_MP
EPI_ISL_502294_A_canine_Ontario_NCFAD_2018_070_11_2018_MP
CN_RLJ_1_13_H3N2__M
CN_HLJ_1_18_H3N2__M
EPI_ISL_17477152_A_canine_Beijing_1016_2018_MP
EPI_ISL_379526_A_canine_Beijing_20170604_164_2017_MP
EPI_ISL_308875_A_canine_Florida_20170606_7_2017_MP
EPI_ISL_308881_A_canine_NorthCarolina_20170606_28_2017_MP
EPI_ISL_308892_A_canine_Florida_20170606_11_2017_MP
EPI_ISL_329171_A_canine_Florida_090919_2017_MP
EPI_ISL_308884_A_canine_Florida_20170606_8_2017_MP
EPI_ISL_379529_A_canine_Beijing_20170528_147_2017_MP
EPI_ISL_308893_A_canine_Kentucky_20170606_6_2017_MP
EPI_ISL_329161_A_canine_North_Carolina_090487_2017_MP
EPI_ISL_308894_A_canine_Florida_20170606_2_2017_MP
EPI_ISL_379530_A_canine_Beijing_20170527_146_2017_MP
EPI_ISL_328524_A_canine_Florida_88807_2_2017_MP
EPI_ISL_328523_A_canine_Georgia_89750_1_2017_MP
EPI_ISL_328522_A_canine_Georgia_20170606_19_2017_MP
EPI_ISL_308879_A_canine_Kentucky_20170606_23_2017_MP
EPI_ISL_308877_A_canine_Florida_20170606_9_2017_MP
EPI_ISL_308878_A_canine_Florida_20170606_17_2017_MP
EPI_ISL_308876_A_canine_Florida_20170606_18_2017_MP
EPI_ISL_329151_A_canine_Ohio_A82_2017_MP
EPI_ISL_372287_A_Canis_lupus_familiaris_USA_118191_2017_MP
EPI_ISL_372289_A_Canis_lupus_familiaris_USA_188226_2017_MP
EPI_ISL_329154_A_canine_South_Carolina_132956_2017_MP
EPI_ISL_329158_A_canine_Kentucky_128656_2017_MP
EPI_ISL_329159_A_canine_Kentucky_174490_2017_MP
EPI_ISL_329153_A_canine_Ohio_A97_2017_MP
EPI_ISL_372281_A_Canis_lupus_familiaris_USA_007780_2018_MP
EPI_ISL_329162_A_canine_Ohio_174509_2017_MP
EPI_ISL_372290_A_Canis_lupus_familiaris_USA_188297_2017_MP
EPI_ISL_329157_A_canine_Indiana_130636_2017_MP
EPI_ISL_372291_A_Canis_lupus_familiaris_USA_188305_2017_MP
EPI_ISL_372288_A_Canis_lupus_familiaris_USA_188203_2017_MP
EPI_ISL_372279_A_Canis_lupus_familiaris_USA_000915_2018_MP
EPI_ISL_329172_A_canine_Illinois_A37_2017_MP
EPI_ISL_329152_A_canine_Ohio_A91_2017_MP
EPI_ISL_329160_A_canine_Minnesota_139684_2017_MP
EPI_ISL_379505_A_canine_Beijing_20161228_9_2016_MP
EPI_ISL_379506_A_canine_Beijing_20161216_8_2016_MP
EPI_ISL_379513_A_canine_Beijing_20170720_30_2017_MP
EPI_ISL_379518_A_canine_Beijing_20170616_202_2017_MP
EPI_ISL_379536_A_canine_Beijing_20170510_118_2017_MP
EPI_ISL_328526_A_canine_California_84315_2017_MP
EPI_ISL_379514_A_canine_Beijing_20170712_18_2017_MP
EPI_ISL_379516_A_canine_Beijing_20170709_15_2017_MP
EPI_ISL_379539_A_canine_Beijing_20170417_83_2017_MP
EPI_ISL_379498_A_canine_Nanjing_20170704_53_2017_MP
EPI_ISL_379503_A_canine_Nanjing_20170328_7_2017_MP
EPI_ISL_379499_A_canine_Nanjing_20170620_47_2017_MP
EPI_ISL_379502_A_canine_Nanjing_20170419_12_2017_MP
EPI_ISL_379500_A_canine_Nanjing_20170607_40_2017_MP
EPI_ISL_379501_A_canine_Nanjing_20170515_25_2017_MP
EPI_ISL_379496_A_canine_Shanghai_20170713_19_2017_MP
EPI_ISL_379497_A_canine_Shanghai_20170622_7_2017_MP
EPI_ISL_379495_A_canine_Shanghai_20170721_36_2017_MP
EPI_ISL_502287_A_canine_Ontario_NCFAD_2018_064_1_2018_MP
EPI_ISL_502291_A_canine_Ontario_NCFAD_2018_069_9_2018_MP
EPI_ISL_502289_A_canine_Ontario_NCFAD_2018_069_10_2018_MP
EPI_ISL_502290_A_canine_Ontario_NCFAD_2018_069_6_2018_MP
EPI_ISL_502288_A_canine_Ontario_NCFAD_2018_069_1_2018_MP
EPI_ISL_379541_A_canine_Beijing_20170314_73_2017_MP
EPI_ISL_17693234_A_canine_Minnesota_012684_2023_MP
EPI_ISL_18750852_A_canine_Texas_CVM_04914311_2023_MP
EPI_ISL_18751256_A_canine_Texas_CVM_09336311_2023_MP
EPI_ISL_18752209_A_canine_Texas_CVM_042018_3_2023_MP
EPI_ISL_18751257_A_canine_Texas_CVM_09336411_2023_MP
EPI_ISL_18751276_A_canine_Texas_CVM_09204151_2023_MP
EPI_ISL_18751258_A_canine_Texas_CVM_09336441_2023_MP
EPI_ISL_18751277_A_canine_Texas_CVM_09201821_2023_MP
EPI_ISL_18751259_A_canine_Texas_CVM_09336521_2023_MP
EPI_ISL_18751255_A_canine_Texas_CVM_09336221_2023_MP
EPI_ISL_18750851_A_canine_Texas_CVM_04914241_2023_MP
EPI_ISL_18751254_A_canine_Texas_CVM_09336121_2023_MP
EPI_ISL_18750853_A_canine_Texas_CVM_06880321_2023_MP
EPI_ISL_18751275_A_canine_Texas_CVM_09204171_2023_MP
EPI_ISL_18752206_A_canine_Pennsylvania_CVM_037756_1_2023_MP
EPI_ISL_17693235_A_canine_Oklahoma_011242_2023_MP
EPI_ISL_18751252_A_canine_Texas_CVM_0688041_2023_MP
EPI_ISL_18751253_A_canine_Texas_CVM_0688071_2023_MP
EPI_ISL_18750734_A_canine_Pennsylvania_CVM_985419_2022_MP
EPI_ISL_18751260_A_canine_Virginia_CVM_00291221_2023_MP
EPI_ISL_18752205_A_canine_Alabama_CVM_194456_1_2022_MP
EPI_ISL_18752207_A_canine_Tennessee_CVM_231361_1_2022_MP
EPI_ISL_18750735_A_canine_Texas_CVM_3578541_2022_MP
EPI_ISL_18751261_A_canine_West_Virginia_CVM_120508_1_2023_MP
EPI_ISL_4031576_A_canine_Guangdong_DY1_2019_MP
EPI_ISL_18690799_A_canine_Beijing_CAU_511_2011_MP
EPI_ISL_18690798_A_canine_Beijing_CAU_510_2010_MP
EPI_ISL_4061869_A_canine_Illinois_21_015197_001_2021_MP
EPI_ISL_18690802_A_canine_Hainan_CAU_W17_2015_MP
EPI_ISL_4061898_A_canine_Illinois_21_015197_005_2021_MP
EPI_ISL_18690800_A_canine_Beijing_CAU_JC2538_2010_MP
EPI_ISL_18750730_A_canine_Florida_CVM_81_2021_MP
EPI_ISL_18750646_A_canine_California_CVM_32151I01_2021_MP
EPI_ISL_18750647_A_canine_California_CVM_32151I41_2021_MP
EPI_ISL_18750729_A_canine_California_CVM_3466381_2021_MP
EPI_ISL_4061925_A_canine_Texas_21_011409_001_2021_MP
EPI_ISL_18690801_A_canine_Beijing_CAU_JC2551_2010_MP
EPI_ISL_4168036_A_canine_California_LACPHL_INF00006_2021_MP
EPI_ISL_18750733_A_canine_Florida_CVM_H1_2021_MP
EPI_ISL_18750728_A_canine_California_CVM_33954291_2021_MP
CN_HLJ_2_13_H3N2__M
CN_RLJ_3_19_H3N2__M
EPI_ISL_18750732_A_canine_Florida_CVM_F1_2021_MP
EPI_ISL_18690797_A_canine_Beijing_CAU_501_2001_MP
EPI_ISL_18750723_A_canine_California_CVM_3235751_2021_MP
EPI_ISL_18750727_A_canine_California_CVM_3235921_2021_MP
EPI_ISL_18750726_A_canine_California_CVM_32918141_2021_MP
EPI_ISL_18750724_A_canine_California_CVM_3275921_2021_MP
EPI_ISL_17277265_A_canine_China_6068_2018_MP
EPI_ISL_17277337_A_canine_China_15068_2019_MP
EPI_ISL_17277336_A_canine_China_15066_2019_MP
EPI_ISL_17277335_A_canine_China_15027_2019_MP
EPI_ISL_499086_A_canine_Guangdong_2_2018_MP
EPI_ISL_17277266_A_canine_China_15001_2019_MP
EPI_ISL_372280_A_Canis_lupus_familiaris_USA_006974_2018_MP
EPI_ISL_502286_A_canine_Ontario_NCFAD_2018_001_4_2018_MP
EPI_ISL_372293_A_canine_California_BRW002_2018_MP
EPI_ISL_372296_A_canine_Nevada_BRW018_2018_MP
EPI_ISL_502300_A_canine_Ontario_NCFAD_2018_070_2_2018_MP
EPI_ISL_502292_A_canine_Ontario_NCFAD_2018_070_1_2018_MP
EPI_ISL_372297_A_Canis_lupus_familiaris_USA_218592_2017_MP
EPI_ISL_502301_A_canine_Ontario_NCFAD_2018_070_3_2018_MP
EPI_ISL_372295_A_canine_Nevada_BRW015_2018_MP
EPI_ISL_502302_A_canine_Ontario_NCFAD_2018_070_4_2018_MP
EPI_ISL_502303_A_canine_Ontario_NCFAD_2018_070_5_2018_MP
EPI_ISL_372282_A_Canis_lupus_familiaris_USA_007781_2018_MP
EPI_ISL_372284_A_Canis_lupus_familiaris_USA_150620_2018_MP
EPI_ISL_372285_A_Canis_lupus_familiaris_USA_152366_2018_MP
EPI_ISL_372297_A_canine_New_Jersey_CRP036_2018_MP
EPI_ISL_372298_A_canine_New_York_CRP034_2018_MP
EPI_ISL_372294_A_canine_California_BRW003_2018_MP
EPI_ISL_308874_A_canine_South_Korea_20170110_1F1_2018_MP
EPI_ISL_190463_A_canine_Illinois_12191_2015_MP
EPI_ISL_284694_A_canine_Indiana_003018_2016_MP
EPI_ISL_234611_A_canine_Illinois_2883703_2015_MP
EPI_ISL_234606_A_canine_North_Carolina_109904_2015_MP
EPI_ISL_329164_A_canine_Illinois_278507_2016_MP
EPI_ISL_284695_A_canine_Florida_29977O_2015_MP
EPI_ISL_234669_A_canine_Texas_343907_2015_MP
EPI_ISL_234608_A_canine_Illinois_1619144_2015_MP
EPI_ISL_284697_A_canine_Illinois_283066_2015_MP
EPI_ISL_329177_A_canine_Illinois_41915_2015_MP
EPI_ISL_329170_A_canine_Indiana_2093881_2015_MP
EPI_ISL_503149_A_canine_Georgia_42632_2015_MP
EPI_ISL_329165_A_canine_Texas_2100186_2015_MP
EPI_ISL_284698_A_canine_Illinois_328292_2015_MP
EPI_ISL_234607_A_canine_Georgia_104940_2015_MP
EPI_ISL_329184_A_canine_Georgia_101875_2015_MP
EPI_ISL_234610_A_canine_Georgia_95391_2015_MP
EPI_ISL_234609_A_canine_Indiana_96198_2015_MP
EPI_ISL_329166_A_canine_North_Carolina_103257_2015_MP
EPI_ISL_234604_A_canine_Wisconsin_19137_2016_MP
EPI_ISL_308891_A_canine_Illinois_M16_25374_19_1_2016_MP
EPI_ISL_284696_A_canine_Illinois_077753_2016_MP
EPI_ISL_329168_A_canine_Illinois_077752_2016_MP
EPI_ISL_329174_A_canine_Illinois_10352389_2015_MP
EPI_ISL_234605_A_canine_Illinois_0843093_2015_MP
EPI_ISL_17805983_A_dog_Indiana_M16_09378_32_1_2016_MP
EPI_ISL_308898_A_canine_Illinois_20170110_1C4_2016_MP
EPI_ISL_329173_A_canine_Indiana_132276_16_1_2_2016_MP
EPI_ISL_329169_A_canine_Illinois_145135_16_1_1_2016_MP
EPI_ISL_329167_A_canine_Illinois_20170110_1A4_2016_MP
EPI_ISL_329183_A_canine_Illinois_091375_16_1_1_2016_MP
EPI_ISL_329175_A_canine_Indiana_093165_16_1_1_2016_MP
EPI_ISL_308882_A_canine_Illinois_187427_16_7_2016_MP
EPI_ISL_308886_A_canine_Illinois_20170110_1G12_2016_MP
EPI_ISL_308880_A_canine_Illinois_M16_30480_3_1_2016_MP
EPI_ISL_329178_A_canine_Indiana_17031_16_2_2016_MP
EPI_ISL_308895_A_canine_Illinois_20170110_2G1_2016_MP
EPI_ISL_308897_A_canine_Illinois_20170110_2F1_2016_MP
EPI_ISL_308873_A_canine_Illinois_M16_25374_14_1_2016_MP
EPI_ISL_308896_A_canine_Illinois_M17_05782_7_1_2017_MP
EPI_ISL_308890_A_canine_Illinois_M17_05782_4_1_2017_MP
EPI_ISL_308889_A_canine_Illinois_M17_05782_1_1_2017_MP
EPI_ISL_308888_A_canine_Illinois_M16_16391_2_1_2016_MP
EPI_ISL_308885_A_canine_Pennsylvania_20170411_5_2017_MP
EPI_ISL_308887_A_canine_Illinois_M17_05782_11_1_2017_MP
EPI_ISL_329176_A_canine_Indiana_1177_17_1_2017_MP
EPI_ISL_308883_A_canine_Illinois_M17_05782_8_1_2017_MP
EPI_ISL_329163_A_canine_Pennsylvania_0261885_2015_MP
EPI_ISL_234613_A_canine_Korea_0589318_2015_MP
EPI_ISL_284693_A_canine_South_Korea_0173915_2015_MP
EPI_ISL_218443_A_canine_Korea_BD_1_2013_MP
EPI_ISL_379508_A_canine_Beijing_20131207_365_2013_MP
EPI_ISL_379546_A_canine_Beijing_20140111_2014_MP
EPI_ISL_379511_A_canine_Beijing_20131203_341_2013_MP
EPI_ISL_379512_A_canine_Beijing_20131112_279_2013_MP
EPI_ISL_379510_A_canine_Beijing_20131203_342_2013_MP
EPI_ISL_379547_A_canine_Beijing_20140103_90_2014_MP
EPI_ISL_308872_A_canine_California_20170411_20_2017_MP
EPI_ISL_328525_A_canine_California_043670_2017_MP
EPI_ISL_379504_A_canine_Beijing_20121230_62_2012_MP
EPI_ISL_379545_A_canine_Beijing_20150118_256_2015_MP
EPI_ISL_379509_A_canine_Beijing_20131204_349_2013_MP
EPI_ISL_297933_A_canine_China_LG1_2015_MP
EPI_ISL_297934_A_canine_China_LG2_2015_MP
EPI_ISL_285503_A_canine_China_JLM2_2015_MP
EPI_ISL_285502_A_canine_China_JLM1_2015_MP
EPI_ISL_379507_A_canine_Beijing_20121215_34_2012_MP
EPI_ISL_379543_A_canine_Beijing_20150122_284_2015_MP
EPI_ISL_379544_A_canine_Beijing_20150120_265_2015_MP
EPI_ISL_151145_A_canine_Guangdong_12_2012_MP
EPI_ISL_151146_A_canine_Guangdong_23_2012_MP
EPI_ISL_137602_A_canine_Thailand_CU_DC5299_2012_MP
EPI_ISL_162436_A_canine_Liaoning_27_2012_MP
EPI_ISL_162438_A_canine_Liaoning_H6_2012_MP
EPI_ISL_162440_A_canine_Heilongjiang_11_2013_MP
EPI_ISL_93813_A_canine_Jiangsu_05_2010_MP
EPI_ISL_93817_A_canine_Jiangsu_01_2009_MP
EPI_ISL_93815_A_canine_Jiangsu_03_2010_MP
EPI_ISL_93816_A_canine_Jiangsu_02_2010_MP
EPI_ISL_93814_A_canine_Jiangsu_04_2010_MP
EPI_ISL_138948_A_canine_Guangdong_05_2011_MP
EPI_ISL_93812_A_canine_Jiangsu_06_2010_MP
EPI_ISL_129993_A_canine_Zhejiang_1_2010_MP
EPI_ISL_80506_A_canine_Guangdong_2_2006_MP
EPI_ISL_80507_A_canine_Guangdong_2_2007_MP
EPI_ISL_80508_A_canine_Guangdong_1_2007_MP
EPI_ISL_80505_A_canine_Guangdong_1_2006_MP
EPI_ISL_138947_A_canine_Guangdong_1_2011_MP
EPI_ISL_128938_A_canine_Guangdong_2_2011_MP
EPI_ISL_142881_A_canine_Guangdong_3_2011_MP
EPI_ISL_127502_A_canine_Korea_01_2007_MP
EPI_ISL_329146_A_canine_Guangdong_03_2013_MP
EPI_ISL_329149_A_canine_Guangdong_02_2014_MP
EPI_ISL_329148_A_canine_Guangdong_01_2014_MP
EPI_ISL_329147_A_canine_Guangdong_04_2013_MP
EPI_ISL_309181_A_canine_Korea_AS_01_2009_MP
EPI_ISL_170565_A_canine_Korea_S1_2012_MP
EPI_ISL_129987_A_canine_Korea_KRNBB01_2011_MP
EPI_ISL_279526_A_canine_Korea_CY053_2014_MP
EPI_ISL_138997_A_canine_Korea_CY009_2010_MP
EPI_ISL_309184_A_canine_Korea_AS_09_2012_MP
EPI_ISL_309187_A_canine_Korea_AS_09_2012_MP
EPI_ISL_309186_A_canine_Korea_AS_08_2012_MP
EPI_ISL_217479_A_canine_Korea_DG1_2014_MP
EPI_ISL_309182_A_canine_Korea_AS_03_2012_MP
EPI_ISL_309190_A_canine_Korea_AS_15_2013_MP
EPI_ISL_309185_A_canine_Korea_AS_06_2012_MP
EPI_ISL_309183_A_canine_Korea_AS_04_2012_MP
EPI_ISL_309189_A_canine_Korea_AS_14_2013_MP
A_canine_Korea_S3001_2015_H3N2__M
EPI_ISL_309188_A_canine_Korea_AS_11_2013_MP
0.003

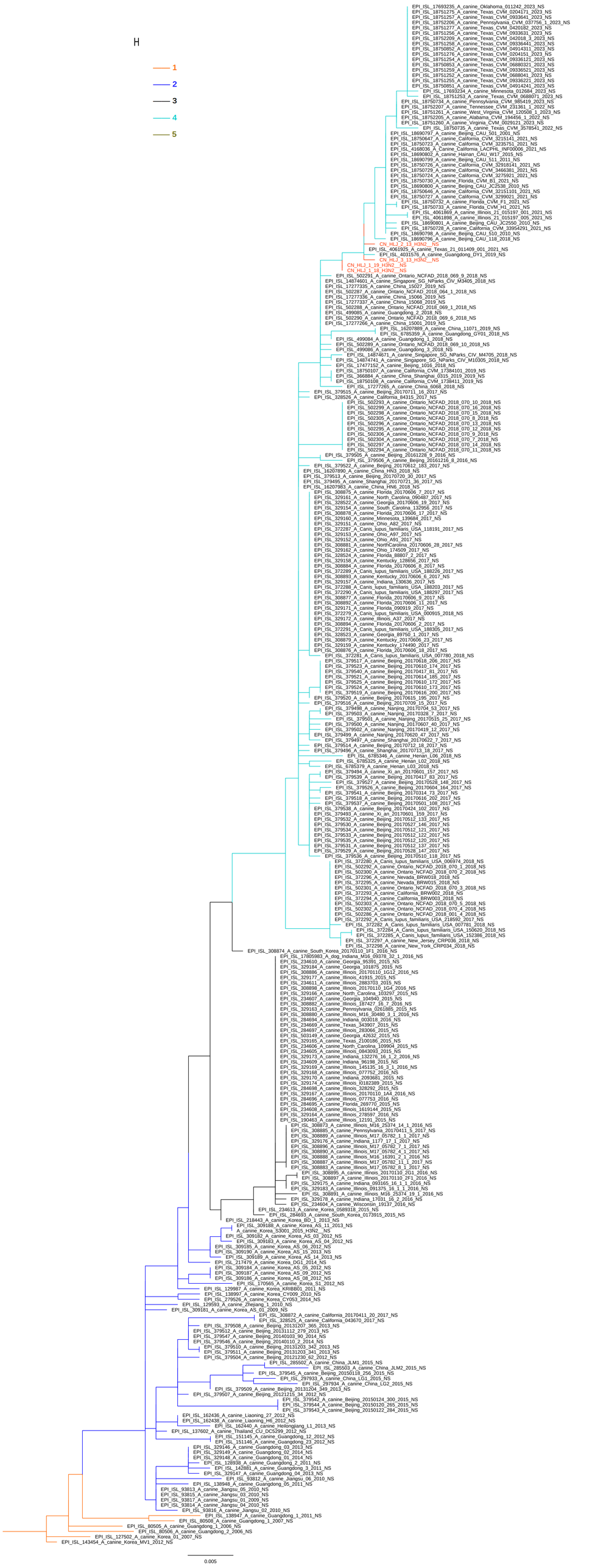
H
1
2
3
4
5
EPI_ISL_17693235_A_canine_Oklahoma_011242_2023_NS
EPI_ISL_18751275_A_canine_Texas_CVM_0204171_2023_NS
EPI_ISL_18751257_A_canine_Texas_CVM_0933641_2023_NS
EPI_ISL_18752206_A_canine_Pennsylvania_CVM_037756_1_2023_NS
EPI_ISL_18751277_A_canine_Texas_CVM_0420182_2023_NS
EPI_ISL_18751256_A_canine_Texas_CVM_0933631_2023_NS
EPI_ISL_18752209_A_canine_Texas_CVM_042018_3_2023_NS
EPI_ISL_18751258_A_canine_Texas_CVM_09336441_2023_NS
EPI_ISL_18750852_A_canine_Pennsylvania_CVM_04914311_2023_NS
EPI_ISL_18751276_A_canine_Texas_CVM_0204151_2023_NS
EPI_ISL_18751254_A_canine_Texas_CVM_09336121_2023_NS
EPI_ISL_18750853_A_canine_Texas_CVM_06880321_2023_NS
EPI_ISL_18751259_A_canine_Texas_CVM_09336521_2023_NS
EPI_ISL_18751252_A_canine_Texas_CVM_0688041_2023_NS
EPI_ISL_18751255_A_canine_Texas_CVM_09336221_2023_NS
EPI_ISL_18750851_A_canine_Texas_CVM_04914241_2023_NS
EPI_ISL_17693234_A_canine_Minnesota_012684_2023_NS
EPI_ISL_18751253_A_canine_Texas_CVM_0688071_2023_NS
EPI_ISL_18750734_A_canine_Pennsylvania_CVM_985419_2023_NS
EPI_ISL_18752207_A_canine_Tennessee_CVM_231361_1_2022_NS
EPI_ISL_18751261_A_canine_West_Virginia_CVM_120508_1_2023_NS
EPI_ISL_18752205_A_canine_Alabama_CVM_194456_1_2022_NS
EPI_ISL_18751260_A_canine_Virginia_CVM_0029121_2023_NS
EPI_ISL_18750735_A_canine_Texas_CVM_3578541_2022_NS
EPI_ISL_18690797_A_canine_Beijing_CAU_501_2001_NS
EPI_ISL_18750647_A_canine_California_CVM_3215141_2021_NS
EPI_ISL_18750723_A_canine_California_CVM_3235751_2021_NS
EPI_ISL_4168036_A_Canine_California_LACPHL_INF00006_2021_NS
EPI_ISL_18690802_A_canine_Hainan_CAU_W17_2015_NS
EPI_ISL_18690799_A_canine_Beijing_CAU_511_2011_NS
EPI_ISL_18750726_A_canine_California_CVM_329181441_2021_NS
EPI_ISL_18750729_A_canine_California_CVM_3466381_2021_NS
EPI_ISL_18750724_A_canine_California_CVM_3275921_2021_NS
EPI_ISL_18750730_A_canine_Florida_CVM_B1_2021_NS
EPI_ISL_18690800_A_canine_Beijing_CAU_JC2539_2010_NS
EPI_ISL_18750646_A_canine_California_CVM_321511O1_2021_NS
EPI_ISL_18750727_A_canine_California_CVM_3299021_2021_NS
EPI_ISL_18750732_A_canine_Florida_CVM_F1_2021_NS
EPI_ISL_18750733_A_canine_Florida_CVM_H1_2021_NS
EPI_ISL_4061869_A_canine_Illinois_21_015197_001_2021_NS
EPI_ISL_4061898_A_canine_Illinois_21_015197_005_2021_NS
EPI_ISL_18690801_A_canine_Beijing_CAU_JC2550_2010_NS
EPI_ISL_18750728_A_canine_California_CVM_33954291_2021_NS
EPI_ISL_18690798_A_canine_Beijing_CAU_510_2010_NS
EPI_ISL_18690796_A_canine_Beijing_CAU_118_2018_NS
CN_HLJ_2_13_H3N2__NS
EPI_ISL_4061925_A_canine_Texas_21_011409_001_2021_NS
EPI_ISL_4031576_A_canine_Guangdong_DY1_2019_NS
CN_HLJ_3_13_H3N2__NS
CN_HLJ_1_19_H3N2__NS
CN_HLJ_1_18_H3N2__NS
EPI_ISL_502291_A_canine_Ontario_NCFAD_2018_069_9_2018_NS
EPI_ISL_14874601_A_canine_Singapore_SG_NParks_CIV_M3405_2018_NS
EPI_ISL_17277335_A_canine_China_15027_2019_NS
EPI_ISL_502287_A_canine_Ontario_NCFAD_2018_064_1_2018_NS
EPI_ISL_17277336_A_canine_China_15066_2019_NS
EPI_ISL_17277337_A_canine_China_15068_2019_NS
EPI_ISL_502288_A_canine_Ontario_NCFAD_2018_069_1_2018_NS
EPI_ISL_499085_A_canine_Guangdong_2_2018_NS
EPI_ISL_502290_A_canine_Ontario_NCFAD_2018_069_6_2018_NS
EPI_ISL_17277266_A_canine_China_15001_2019_NS
EPI_ISL_16207889_A_canine_China_11071_2019_NS
EPI_ISL_6785359_A_canine_Guangdong_GY01_2018_NS
EPI_ISL_499084_A_canine_Guangdong_1_2018_NS
EPI_ISL_502289_A_canine_Ontario_NCFAD_2018_069_10_2018_NS
EPI_ISL_499086_A_canine_Guangdong_3_2018_NS
EPI_ISL_14874671_A_canine_Singapore_SG_NParks_CIV_M4705_2018_NS
EPI_ISL_14874741_A_canine_Singapore_SG_NParks_CIV_M10305_2018_NS
EPI_ISL_17477152_A_canine_Beijing_1016_2018_NS
EPI_ISL_18750107_A_canine_California_CVM_17384101_2019_NS
EPI_ISL_366884_A_canine_China_Shanghai_0315_2019_2019_NS
EPI_ISL_18750108_A_canine_California_CVM_17384111_2019_NS
EPI_ISL_17277265_A_canine_China_6068_2018_NS
EPI_ISL_379515_A_canine_Beijing_20170711_16_2017_NS
EPI_ISL_328526_A_canine_California_84315_2017_NS
EPI_ISL_502293_A_canine_Ontario_NCFAD_2018_070_10_2018_NS
EPI_ISL_502299_A_canine_Ontario_NCFAD_2018_070_16_2018_NS
EPI_ISL_502298_A_canine_Ontario_NCFAD_2018_070_15_2018_NS
EPI_ISL_502305_A_canine_Ontario_NCFAD_2018_070_8_2018_NS
EPI_ISL_502296_A_canine_Ontario_NCFAD_2018_070_13_2018_NS
EPI_ISL_502295_A_canine_Ontario_NCFAD_2018_070_12_2018_NS
EPI_ISL_502306_A_canine_Ontario_NCFAD_2018_070_9_2018_NS
EPI_ISL_502304_A_canine_Ontario_NCFAD_2018_070_7_2018_NS
EPI_ISL_502297_A_canine_Ontario_NCFAD_2018_070_14_2018_NS
EPI_ISL_502294_A_canine_Ontario_NCFAD_2018_070_11_2018_NS
EPI_ISL_379505_A_canine_Beijing_20161228_9_2016_NS
EPI_ISL_379506_A_canine_Beijing_20161216_8_2016_NS
EPI_ISL_379522_A_canine_Beijing_20170612_183_2017_NS
EPI_ISL_16207890_A_canine_China_HN3_2018_NS
EPI_ISL_379513_A_canine_Beijing_20170720_30_2017_NS
EPI_ISL_379495_A_canine_Shanghai_20170721_36_2017_NS
EPI_ISL_16207983_A_canine_China_HN6_2018_NS
EPI_ISL_308875_A_canine_Florida_20170606_7_2017_NS
EPI_ISL_329161_A_canine_North_Carolina_090487_2017_NS
EPI_ISL_328522_A_canine_Georgia_20170606_19_2017_NS
EPI_ISL_329154_A_canine_South_Carolina_132956_2017_NS
EPI_ISL_308878_A_canine_Florida_20170606_17_2017_NS
EPI_ISL_329160_A_canine_Minnesota_139684_2017_NS
EPI_ISL_329151_A_canine_Ohio_A82_2017_NS
EPI_ISL_372287_A_Canis_lupus_familiaris_USA_118191_2017_NS
EPI_ISL_329153_A_canine_Ohio_A97_2017_NS
EPI_ISL_329152_A_canine_Ohio_A91_2017_NS
EPI_ISL_308881_A_canine_NorthCarolina_20170606_28_2017_NS
EPI_ISL_329162_A_canine_Ohio_174509_2017_NS
EPI_ISL_328524_A_canine_Florida_88807_2_2017_NS
EPI_ISL_329158_A_canine_Kentucky_128856_2017_NS
EPI_ISL_308884_A_canine_Florida_20170606_8_2017_NS
EPI_ISL_372289_A_Canis_lupus_familiaris_USA_188226_2017_NS
EPI_ISL_308893_A_canine_Kentucky_20170606_6_2017_NS
EPI_ISL_329157_A_canine_Indiana_130636_2017_NS
EPI_ISL_372288_A_Canis_lupus_familiaris_USA_188203_2017_NS
EPI_ISL_372290_A_Canis_lupus_familiaris_USA_188297_2017_NS
EPI_ISL_308877_A_canine_Florida_20170606_9_2017_NS
EPI_ISL_308892_A_canine_Florida_20170606_11_2017_NS
EPI_ISL_329171_A_canine_Florida_090919_2017_NS
EPI_ISL_372279_A_Canis_lupus_familiaris_USA_000915_2018_NS
EPI_ISL_329172_A_canine_Illinois_A37_2017_NS
EPI_ISL_308894_A_canine_Florida_20170606_2_2017_NS
EPI_ISL_372291_A_Canis_lupus_familiaris_USA_188305_2017_NS
EPI_ISL_328523_A_canine_Georgia_89750_1_2017_NS
EPI_ISL_308879_A_canine_Kentucky_20170606_23_2017_NS
EPI_ISL_329159_A_canine_Kentucky_174490_2017_NS
EPI_ISL_308876_A_canine_Florida_20170606_18_2017_NS
EPI_ISL_372281_A_Canis_lupus_familiaris_USA_007780_2018_NS
EPI_ISL_379517_A_canine_Beijing_20170618_206_2017_NS
EPI_ISL_379523_A_canine_Beijing_20170610_174_2017_NS
EPI_ISL_379540_A_canine_Beijing_20170417_81_2017_NS
EPI_ISL_379521_A_canine_Beijing_20170614_185_2017_NS
EPI_ISL_379525_A_canine_Beijing_20170610_172_2017_NS
EPI_ISL_379524_A_canine_Beijing_20170610_173_2017_NS
EPI_ISL_379519_A_canine_Beijing_20170616_200_2017_NS
EPI_ISL_379520_A_canine_Beijing_20170615_195_2017_NS
EPI_ISL_379516_A_canine_Beijing_20170709_15_2017_NS
EPI_ISL_379498_A_canine_Nanjing_20170704_53_2017_NS
EPI_ISL_379503_A_canine_Nanjing_20170328_7_2017_NS
EPI_ISL_379501_A_canine_Nanjing_20170515_25_2017_NS
EPI_ISL_379500_A_canine_Nanjing_20170607_40_2017_NS
EPI_ISL_379502_A_canine_Nanjing_20170419_12_2017_NS
EPI_ISL_379499_A_canine_Nanjing_20170620_47_2017_NS
EPI_ISL_379497_A_canine_Shanghai_20170622_7_2017_NS
EPI_ISL_379514_A_canine_Beijing_20170712_18_2017_NS
EPI_ISL_379496_A_canine_Shanghai_20170713_18_2017_NS
EPI_ISL_6785346_A_canine_Henan_L06_2018_NS
EPI_ISL_6785325_A_canine_Henan_L02_2018_NS
EPI_ISL_6785379_A_canine_Henan_L03_2018_NS
EPI_ISL_379494_A_canine_Xi_an_20170601_157_2017_NS
EPI_ISL_379539_A_canine_Beijing_20170417_83_2017_NS
EPI_ISL_379527_A_canine_Beijing_20170528_148_2017_NS
EPI_ISL_379526_A_canine_Beijing_20170604_164_2017_NS
EPI_ISL_379541_A_canine_Beijing_20170314_73_2017_NS
EPI_ISL_379518_A_canine_Beijing_20170616_202_2017_NS
EPI_ISL_379537_A_canine_Beijing_20170501_108_2017_NS
EPI_ISL_379538_A_canine_Beijing_20170424_102_2017_NS
EPI_ISL_379493_A_canine_Xi_an_20170601_159_2017_NS
EPI_ISL_379532_A_canine_Beijing_20170512_133_2017_NS
EPI_ISL_379530_A_canine_Beijing_20170527_146_2017_NS
EPI_ISL_379534_A_canine_Beijing_20170512_121_2017_NS
EPI_ISL_379533_A_canine_Beijing_20170512_122_2017_NS
EPI_ISL_379535_A_canine_Beijing_20170512_120_2017_NS
EPI_ISL_379531_A_canine_Beijing_20170512_137_2017_NS
EPI_ISL_379529_A_canine_Beijing_20170528_147_2017_NS
EPI_ISL_379536_A_canine_Beijing_20170510_118_2017_NS
EPI_ISL_372280_A_Canis_lupus_familiaris_USA_006974_2018_NS
EPI_ISL_502292_A_canine_Ontario_NCFAD_2018_070_1_2018_NS
EPI_ISL_502300_A_canine_Ontario_NCFAD_2018_070_2_2018_NS
EPI_ISL_372296_A_canine_Nevada_BRW018_2018_NS
EPI_ISL_372295_A_canine_Nevada_BRW015_2018_NS
EPI_ISL_502301_A_canine_Ontario_NCFAD_2018_070_3_2018_NS
EPI_ISL_372293_A_canine_California_BRW002_2018_NS
EPI_ISL_372294_A_canine_California_BRW003_2018_NS
EPI_ISL_502303_A_canine_Ontario_NCFAD_2018_070_5_2018_NS
EPI_ISL_502302_A_canine_Ontario_NCFAD_2018_070_4_2018_NS
EPI_ISL_502286_A_canine_Ontario_NCFAD_2018_001_4_2018_NS
EPI_ISL_372292_A_Canis_lupus_familiaris_USA_218592_2017_NS
EPI_ISL_372282_A_Canis_lupus_familiaris_USA_007781_2018_NS
EPI_ISL_372284_A_Canis_lupus_familiaris_USA_150620_2018_NS
EPI_ISL_372285_A_Canis_lupus_familiaris_USA_152386_2018_NS
EPI_ISL_372297_A_canine_New_Jersey_CRP036_2018_NS
EPI_ISL_372298_A_canine_New_York_CRP034_2018_NS
EPI_ISL_308874_A_canine_South_Korea_20170110_1F1_2016_NS
EPI_ISL_17805983_A_dog_Indiana_M16_09378_32_1_2016_NS
EPI_ISL_234610_A_canine_Georgia_95391_2015_NS
EPI_ISL_329184_A_canine_Georgia_101875_2015_NS
EPI_ISL_308886_A_canine_Illinois_20170110_1G12_2016_NS
EPI_ISL_329177_A_canine_Illinois_41915_2015_NS
EPI_ISL_234611_A_canine_Illinois_2883703_2015_NS
EPI_ISL_308898_A_canine_Illinois_20170110_1G4_2016_NS
EPI_ISL_329166_A_canine_North_Carolina_103297_2015_NS
EPI_ISL_234607_A_canine_Georgia_104940_2015_NS
EPI_ISL_308882_A_canine_Illinois_187427_16_7_2016_NS
EPI_ISL_329163_A_canine_Pennsylvania_0261885_2015_NS
EPI_ISL_308880_A_canine_Illinois_M16_30480_3_1_2016_NS
EPI_ISL_284694_A_canine_Indiana_003018_2016_NS
EPI_ISL_234669_A_canine_Texas_343907_2015_NS
EPI_ISL_284697_A_canine_Illinois_283066_2015_NS
EPI_ISL_503149_A_canine_Georgia_42632_2015_NS
EPI_ISL_329165_A_canine_Texas_2100186_2015_NS
EPI_ISL_234606_A_canine_North_Carolina_109904_2015_NS
EPI_ISL_234605_A_canine_Illinois_0843093_2015_NS
EPI_ISL_329173_A_canine_Indiana_132276_16_1_2_2016_NS
EPI_ISL_234609_A_canine_Indiana_96198_2015_NS
EPI_ISL_329169_A_canine_Illinois_145135_16_3_1_2016_NS
EPI_ISL_329168_A_canine_Illinois_077752_2016_NS
EPI_ISL_329170_A_canine_Indiana_2093681_2015_NS
EPI_ISL_329174_A_canine_Illinois_I0182389_2015_NS
EPI_ISL_284698_A_canine_Illinois_328292_2015_NS
EPI_ISL_329167_A_canine_Illinois_20170110_1A4_2016_NS
EPI_ISL_284696_A_canine_Illinois_077753_2016_NS
EPI_ISL_284695_A_canine_Florida_269770_2015_NS
EPI_ISL_234608_A_canine_Illinois_1619144_2015_NS
EPI_ISL_329164_A_canine_Illinois_278597_2016_NS
EPI_ISL_190463_A_canine_Illinois_12191_2015_NS
EPI_ISL_308873_A_canine_Illinois_M16_25374_14_1_2016_NS
EPI_ISL_308885_A_canine_Pennsylvania_20170411_5_2017_NS
EPI_ISL_308889_A_canine_Illinois_M17_05782_1_1_2017_NS
EPI_ISL_329176_A_canine_Indiana_1177_17_1_2017_NS
EPI_ISL_308896_A_canine_Illinois_M17_05782_7_1_2017_NS
EPI_ISL_308890_A_canine_Illinois_M17_05782_4_1_2017_NS
EPI_ISL_308888_A_canine_Illinois_M16_16391_2_1_2016_NS
EPI_ISL_308887_A_canine_Illinois_M17_05782_11_1_2017_NS
EPI_ISL_308883_A_canine_Illinois_M17_05782_9_1_2017_NS
EPI_ISL_308895_A_canine_Illinois_20170110_2G1_2016_NS
EPI_ISL_308897_A_canine_Illinois_20170110_2F1_2016_NS
EPI_ISL_329175_A_canine_Indiana_093165_16_1_1_2016_NS
EPI_ISL_329183_A_canine_Illinois_091375_16_1_1_2016_NS
EPI_ISL_308891_A_canine_Illinois_M16_25374_19_1_2016_NS
EPI_ISL_329178_A_canine_Indiana_17091_16_2_2016_NS
EPI_ISL_234604_A_canine_Wisconsin_19137_2016_NS
EPI_ISL_234613_A_canine_Korea_0589318_2015_NS
EPI_ISL_284693_A_canine_South_Korea_0173915_2015_NS
EPI_ISL_218443_A_canine_Korea_BD_1_2013_NS
EPI_ISL_309188_A_canine_Korea_AS_11_2013_NS
A_canine_Korea_S3001_2015_H3N2__NS
EPI_ISL_309182_A_canine_Korea_AS_03_2012_NS
EPI_ISL_309183_A_canine_Korea_AS_04_2012_NS
EPI_ISL_309185_A_canine_Korea_AS_06_2012_NS
EPI_ISL_309190_A_canine_Korea_AS_15_2013_NS
EPI_ISL_309189_A_canine_Korea_AS_14_2013_NS
EPI_ISL_217479_A_canine_Korea_DG1_2014_NS
EPI_ISL_309184_A_canine_Korea_AS_05_2012_NS
EPI_ISL_309187_A_canine_Korea_AS_09_2012_NS
EPI_ISL_309186_A_canine_Korea_AS_08_2012_NS
EPI_ISL_170565_A_canine_Korea_S1_2012_NS
EPI_ISL_129987_A_canine_Korea_KRIBB01_2011_NS
EPI_ISL_138997_A_canine_Korea_CY009_2010_NS
EPI_ISL_279526_A_canine_Korea_CY053_2014_NS
EPI_ISL_129593_A_canine_Zhejang_1_2010_NS
EPI_ISL_309181_A_canine_Korea_AS_01_2009_NS
EPI_ISL_308872_A_canine_California_20170411_20_2017_NS
EPI_ISL_328525_A_canine_California_043670_2017_NS
EPI_ISL_379508_A_canine_Beijing_20131207_365_2013_NS
EPI_ISL_379512_A_canine_Beijing_20131112_279_2013_NS
EPI_ISL_379547_A_canine_Beijing_20140103_90_2014_NS
EPI_ISL_379546_A_canine_Beijing_20140110_2_2014_NS
EPI_ISL_379510_A_canine_Beijing_20131203_342_2013_NS
EPI_ISL_379511_A_canine_Beijing_20131203_341_2013_NS
EPI_ISL_379504_A_canine_Beijing_20121230_62_2012_NS
EPI_ISL_285502_A_canine_China_JLM1_2015_NS
EPI_ISL_285503_A_canine_China_JLM2_2015_NS
EPI_ISL_379545_A_canine_Beijing_20150118_256_2015_NS
EPI_ISL_297933_A_canine_China_LG1_2015_NS
EPI_ISL_297934_A_canine_China_LG2_2015_NS
EPI_ISL_379509_A_canine_Beijing_20131204_349_2013_NS
EPI_ISL_379507_A_canine_Beijing_20121215_34_2012_NS
EPI_ISL_379542_A_canine_Beijing_20150124_300_2015_NS
EPI_ISL_379544_A_canine_Beijing_20150120_265_2015_NS
EPI_ISL_379543_A_canine_Beijing_20150122_284_2015_NS
EPI_ISL_162436_A_canine_Liaoning_27_2012_NS
EPI_ISL_162438_A_canine_Liaoning_H6_2012_NS
EPI_ISL_162440_A_canine_Heilongjiang_L1_2013_NS
EPI_ISL_137602_A_canine_Thailand_CU_DC5299_2012_NS
EPI_ISL_151145_A_canine_Guangdong_12_2012_NS
EPI_ISL_151146_A_canine_Guangdong_23_2012_NS
EPI_ISL_329146_A_canine_Guangdong_03_2013_NS
EPI_ISL_329149_A_canine_Guangdong_02_2014_NS
EPI_ISL_329148_A_canine_Guangdong_01_2014_NS
EPI_ISL_128938_A_canine_Guangdong_2_2011_NS
EPI_ISL_142881_A_canine_Guangdong_3_2011_NS
EPI_ISL_329147_A_canine_Guangdong_04_2013_NS
EPI_ISL_93812_A_canine_Jiangsu_06_2010_NS
EPI_ISL_138948_A_canine_Guangdong_05_2011_NS
EPI_ISL_93813_A_canine_Jiangsu_05_2010_NS
EPI_ISL_93815_A_canine_Jiangsu_03_2010_NS
EPI_ISL_93817_A_canine_Jiangsu_01_2009_NS
EPI_ISL_93814_A_canine_Jiangsu_04_2010_NS
EPI_ISL_93816_A_canine_Jiangsu_02_2010_NS
EPI_ISL_138947_A_canine_Guangdong_1_2011_NS
EPI_ISL_80508_A_canine_Guangdong_1_2007_NS
EPI_ISL_80505_A_canine_Guangdong_1_2006_NS
EPI_ISL_80506_A_canine_Guangdong_2_2006_NS
EPI_ISL_127502_A_canine_Korea_01_2007_NS
EPI_ISL_143454_A_canine_Korea_MV1_2012_NS
0.005
